# Endogenous molecular network reveals two mechanisms of heterogeneity within gastric cancer

**DOI:** 10.18632/oncotarget.3633

**Published:** 2015-04-24

**Authors:** Site Li, Xiaomei Zhu, Bingya Liu, Gaowei Wang, Ping Ao

**Affiliations:** ^1^ Shanghai Center for Systems Biomedicine, Ministry of Education Key Laboratory of Systems Biomedicine, Collaborative Innovation Center of Systems Biomedicine, Shanghai Jiao Tong University, Shanghai 200240, China; ^2^ GenMath, Seattle, WA 98105, USA; ^3^ Shanghai Key Laboratory of Gastric Neoplasms, Shanghai Institute of Digestive Surgery, Ruijin Hospital, Shanghai Jiao Tong University School of Medicine, Shanghai 200025, China; ^4^ State Key Laboratory for Oncogenes and Related Genes, Shanghai Cancer Institute, Shanghai Jiao Tong University School of Medicine, Shanghai 200032, China; ^5^ Department of Physics, Shanghai Jiao Tong University, Shanghai 200240, China

**Keywords:** gastric cancer, endogenous molecular network, intratumor heterogeneity, attractor, systems biology

## Abstract

Intratumor heterogeneity is a common phenomenon and impedes cancer therapy and research. Gastric cancer (GC) cells have generally been classified into two heterogeneous cellular phenotypes, the gastric and intestinal types, yet the mechanisms of maintaining two phenotypes and controlling phenotypic transition are largely unknown. A qualitative systematic framework, the endogenous molecular network hypothesis, has recently been proposed to understand cancer genesis and progression. Here, a minimal network corresponding to such framework was found for GC and was quantified via a stochastic nonlinear dynamical system. We then further extended the framework to address the important question of intratumor heterogeneity quantitatively. The working network characterized main known features of normal gastric epithelial and GC cell phenotypes. Our results demonstrated that four positive feedback loops in the network are critical for GC cell phenotypes. Moreover, two mechanisms that contribute to GC cell heterogeneity were identified: particular positive feedback loops are responsible for the maintenance of intestinal and gastric phenotypes; GC cell progression routes that were revealed by the dynamical behaviors of individual key components are heterogeneous. In this work, we constructed an endogenous molecular network of GC that can be expanded in the future and would broaden the known mechanisms of intratumor heterogeneity.

## INTRODUCTION

Intratumor phenotypic heterogeneity is a defining characteristic of human tumors. Cancer cell populations within tumors manifest various traits, including growth rates [1, 2], metastatic capacity [3], and therapy resistance [4, 5]. Heterogeneity complicates the study and treatment of tumors, because small tumor samples may not be representative of the whole tumor [6], and because cancers often become refractory to treatments [7]. Two models have been proposed to explain how heterogeneity arises and contributes to cancer progression. Clonal evolution theory emphasizes that tumor heterogeneity is the result of heritable genetic and epigenetic variation [8], whereas cancer stem cell (CSC) theory proposes that cancer cells stay in different differentiated states and exhibit tumor heterogeneity [9]. Three sources, genetic mutation, epigenetic variations and tumor microenvironment, which contribute to tumor heterogeneity have been discussed and reviewed [10–14]. Evolving evidence implicates other mechanism in intratumor heterogeneity. For example, considerable plasticity exists between cancer cells [10], tumors without driver mutations and/or epigenetic mutations are found [15–17], genetically divergent cells exhibit similar phenotypes [18], and cells harbor critical genetic mutations while no manifesting cancerous phenotypes [19]. In addition, abundant mosaic copy number variation (CNV) is found in normal human neurons, despite not contributing to the functional heterogeneity of the human brain [20]. Moreover, studies suggest that the relationship between genotype and phenotype is more complex than a one-to-one relation, and a complicated regulatory network may control heterogeneous functional phenotypes [11, 21–25]. Therefore, a systematic understanding of the specific driving forces behind heterogeneous subtypes of cancer via an integrated network is required.

GC is the second-most common cause of global cancer mortality, with an overall 5-year survival rate of approximately 20% [26, 27]. Histologically, human GC has been classified into intestinal and diffuse types by Lauren [28]. It is thought that intestinal-type GC develops from intestinalized mucosae, whereas diffuse-type GC arises in gastric mucosae [28, 29]. At a cellular level, human GC cells can also be classified into two heterogeneous phenotypes based on their phenotypic expression: the gastric epithelial cell type (including surface mucous cells and pyloric gland cells) and the intestinal epithelial cell type (including goblet and intestinal absorptive cell types) [30–32]. The two heterogeneous GC cell phenotypes are found in both intestinal- and diffuse-type GC. In the present work, the intratumor heterogeneity of GC is referred as to the two GC cell phenotypes, gastric and intestinal, which will be the focus of our modeling analysis. Phenotypic transition from gastric- to intestinal-type epithelial cells has been observed in human GC and precancerous lesions [33]. The transcription factor Cdx2 is the key mediator of intestinal differentiation in both normal and aberrant locations [34]. Sox2 and Shh are indicated as essential regulators of gastric differentiation [35, 36]. Increased expression of intestinal transcription factor Cdx2 and decreased expression of gastric transcription factors Sox2 are observed in intestinal metaplasia, a precancerous lesion of intestinal type GC [37]. However, the regulatory mechanism of maintaining two heterogeneous GC phenotypes and phenotypic transition is still largely unknown.

Previously, we have proposed an endogenous molecular network hypothesis to understand the genesis and progression of cancer at a systematic level [25, 38, 39]. The endogenous molecular network is formed by endogenous molecular factors, such as transcription factors, growth factors and cytokines, and the interactions among them through signal transduction pathways and transcriptional interactions. We assume that cell types can be characterized by the activities or concentration of these endogenous factors. The dynamics of biochemical factors representing the endogenous network can be quantitatively described by a set of nonlinear and coupled differential equations. Local attractors, including stable equilibrium or limit cycles, are generated by the nonlinear interactions among endogenous factors. Biologically, these attractors may represent cell phenotypes with obvious or non-obvious biological functions, for example, cell cycling and cell arrest. We also assume that normal tissue cells and heterogeneous cancer cells are endogenous attractors in the endogenous molecular network.

In this work we study the heterogeneity of GC via the endogenous molecular network. In the following section, we first discussed the assumption and procedure of the endogenous network construction using GC as an example. Then, the working network is quantified by a nonlinear dynamic system, and the modeling results are compared with experimental data. The working network reproduces the principal known features of normal gastric epithelium and GC at both module and molecular levels, which suggests that the working model is valid for studying GC. For example, normal gastric epithelial cells mainly exhibited cell cycle arrest and differentiated phenotype, while gastric cancer cells showed proliferative, apoptosis resistant, inflammatory and abnormal differentiated phenotype. Finally, two mechanisms that contribute to the intratumor heterogeneity of GC are identified. One is that particular positive feedback loops are found to be responsible for the maintenance of intestinal and gastric phenotypes of GC cells. Second, 16 transition routes of GC cell progression from a normal attractor to GC cell attractors are identified. Different transition routes are characterized by the dynamic behaviors of individual key molecular components, suggesting that GC cells progression may be heterogeneous.

## MATERIALS AND METHODS

### Biological assumptions and endogenous network construction

**Figure 1:**
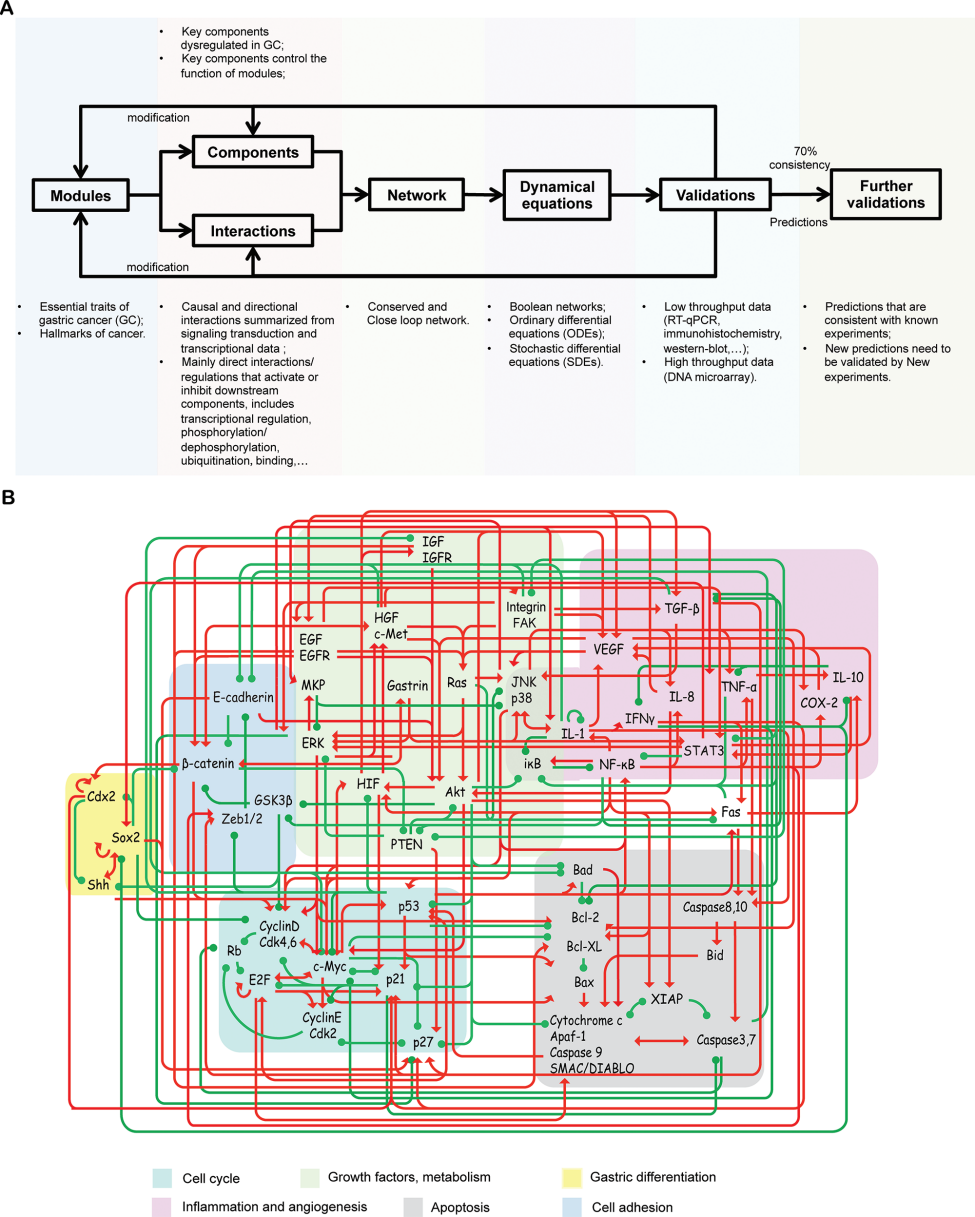
Work flow and the core endogenous network of GC **A.** Work flow of the core endogenous network of GC construction and modeling. **B.** The core network included 8 functional modules, 48 functional components and 215 connections. Differently colored areas represent different functional modules. The interactions include both direct and indirect effects. The interactions can be interpreted as follows: the red arrows and green hammerheads indicate activation and inhibition processes, respectively.

The endogenous molecular network of GC was constructed according to the endogenous molecular network hypothesis [38, 39]. The endogenous molecular network of GC aimed to describe the core regulatory mechanism of GC from a mechanistic perspective; thus we started from the conservative part: core network, which included important functional modules, molecular components and interactions (activation and inhibition). The work flow of network construction and modeling are summarized in Figure 1A. The main assumptions underlying and approaches of constructing the network are listed as follow. First, the endogenous network of GC was assumed to be constructed by a group of functional modules and crosstalk among them. Hanahan and Weinberg [14, 40] have proposed general cancer hallmarks, which provide an organizing principle for understanding the biology of cancer. Moreover, the essential traits of GC have also been reviewed over the past decades [41–43], which are similar to general cancer hallmarks. These essential traits include self-stimulating proliferative signaling, abnormal cell cycling, evading cell death, activating angiogenesis, inducing invasion and metastasis, reprogramming of energy metabolism, inflammation and abnormal gastric differentiation. These distinctive and complimentary traits may represent the main features of GC and provide a logical framework for modeling GC from a mechanistic perspective. Thus, based on general cancer hallmarks, particular dysregulations of signaling pathways and gene signatures for GC, GC was described by a group of essential functional modules, including cell cycle, apoptosis, inflammation, angiogenesis, growth factor signaling pathways, metabolism, cell adhesion and gastric differentiation.

For the same reason, the key components that are dysregulated in GC and control the functional status of modules and their interactions were included in the core network [40–43]. The endogenous factors were assumed to be proteins or protein complexes, because proteins are main executors of biological processes via the role they play in regulating signalling transduction and gene expression. The interactions of endogenous factors are mainly direct activation/upregulation and inhibition/downregulation relationships based on current knowledge of signal transduction pathways and the gene transcription network. The PubMed literature database was used to search for those interaction relations, and a total of 275 publications from 1987 to 2013 were referenced.

Taking the cell cycle module as an example, normal tissues carefully control the cell cycle, thereby ensuring a homeostasis of cell number and thus maintenance of normal tissue architecture and function. In contrast, cancer cells are able to deregulate growth-promoting signaling as well as some negative feedback mechanisms. Thus, in our network, critical kinases and transcription factors (e.g., cyclin D-Cdk4/6, cyclin E-Cdk2, E2F) were included to describe the core molecular mechanisms that control the restriction (R) point of the cell cycle. The activated cyclin D-Cdk4 complex phosphorylates the retinoblastoma protein (Rb). The hyperphosphorylated Rb dissociates from the E2F/Rb complex, activating E2F. Activated E2F then induces the transcription of various genes, such as cyclin E and Cdk2. Cyclin E and Cdk2 form the cyclin E-Cdk2 complex, which pushes the cell go through the R point and to enter the S phase. At a module level, the growth factors module, which involves essential growth factor signals (e.g., EGF, HGF, IGF), instruct cells to enter and process through the cell cycle. For example, the EGF and its receptor activate the transcription of cell-cycle-related genes, such as *myc* and *cyclinD*, by inducing activation of the Ras/MAPK pathway and the PI3K/Akt pathway. In addition, the negative-feedback mechanisms that attenuate proliferative signaling (e.g., PTEN, p21, p27 and p53) were included to characterize the core regulatory mechanism of cancer proliferation. For example, PTEN can dephosphorylate phosphatidylinositol (3,4,5)-trisphosphate (PtdIns (3,4,5)P3 or PIP3) and negatively regulate the Akt/PKB-signaling pathway. The detailed molecular mechanisms of constructing the core endogenous molecular network are summarized in the text under Text S1. Supporting method.

Based on studies of feedback control of intercellular signaling and the transcriptional network [44, 45], another rational assumption was that these endogenous factors and their interactions constitute an autonomous and closed-loop network. The closed-loop network introduces positive or negative feedback at each node in the network: process input has an effect on the process output; the output is fed back as input to the process, closing the loop. Based on the above hypothesis, a core endogenous molecular network for GC has been constructed (Figure 1). These interactions and literature references are summarized in Figure 1A and Supplementary Table S1.

### Quantification of the network

The endogenous molecular network was constructed based on a group of endogenous factors and their interactions. The activity/concentration of each endogenous factor was modulated by other endogenous factors via signal transduction and transcriptional relations. This process can be described by a set of typical chemical rate equations that includes production rate and degradation rate [46]. We assumed that molecular interactions among endogenous factors show a switch-like behavior that can be modeled by the sigmoid-shaped Hill function. Thus, the production rate was quantitatively represented by the Hill functions which have been used to model many biological processes [22, 47, 48]. We take cyclin D-Cdk4/6 complex as an example to illustrate how to construct differential equations. cyclin D-Cdk4/6 can be activated by Myc, NF-κB, ERK, p38, and JNK and inhibited by GSK3β, Sox2 and p21. To re-illustrate the underlying biology of this network, the deterministic description for the cyclin D-Cdk4/6 complex activity/concentration under the influence of Myc, NF-κB, ERK, p38/JNK, GSK3β, Sox2 and p21 takes the form in equation (1): [22, 47–50]

$$\frac{dx_{cyclinD-Cdk4/6}}{dt}=f(Myc,NF-\kappa B,ERK,p38/JNK,Sox2,p21-\frac{x_{cyclinD-Cdk4/6}}{\tau_{cyclinD-Cdk4/z}}$$ (1),

where $x_{cyclinD-Cdk4/6}$ represents the activity/concentration of cyclin D-Cdk4/6 and $\frac{x_{cyclinD-Cdk4/6}}{\tau_{cyclinD-Cdk4/6}}$ represents the degradation rate of cyclin D-Cdk4/6. $\tau_{cyclinD-Cdk4/6}$ is the degradation constant of cyclin D-Cdk4/6, which is normalized to 1. f(Myc, NF−κB, ERK, p38/JNK, Sox2, p21) is the integrated production rate that is modeled by the sigmoid-shaped Hill function in equation (2):

$$\begin{array}{l} f(Myc,NF-\kappa B,ERK,p38/JNK,Sox2,p21)=\frac{a_{1}*x_{Myc}^{n_{1}}+a_{2}*x_{NF-\kappa B}^{n_{2}}+a_{3}*x_{ERK}^{n_{3}}*a_{4}*x_{p38/JNK}^{n_{4}}}{1+a_{1}*x_{Myc}^{n_{1}}+a_{2}*x_{NF-\kappa B}^{n_{2}}+a_{3}*x_{ERK}^{n_{3}}*a_{4}*x_{p38/JNK}^{n_{4}}}*\\ \frac{1}{1+a_{5}*x_{GSK3\beta }^{n_{5}}+a_{6}*x_{Sox2}^{n_{6}}+a_{7}*x_{p21}^{n_{7}}} \end{array}$$ (2),

where $n_{i}$ is the Hill coefficient and $a_{i}$ describes the kinetic properties of each component in regulating the production of the cyclin D-Cdk4/6 complex. The relative activity/concentration of the component was considered with these formulations, and the component activity or concentration was permitted to vary between 0 and 1, which indicates the maximal and minimal activity/concentration, respectively. This assumption will not influence validations, because many experimental data, such as gene expression data, are also measured in a framework of relative concentration. Other components in the network were quantified in the same approach. With the above quantitative assumptions, the network was transformed into a set of coupled ordinary differential equations (Text S1. Supporting method), which represents a nonlinear dynamic system and implies some attractors underlying the endogenous network.

The total numbers of dynamic variables is 48. As the total numbers of interactions is 215 and $n_{i}$ define the cooperativity of each interaction and $a_{i}$ corresponding to $n_{i}$, thus the total number of $n_{i}$ and $a_{i}$ are 215 and 215, respectively. Because the exact values of the parameter $n_{i}$ and $a_{i}$ in the function are not known, several assumptions were made based on biological literature. First, the activating (red lines) and inhibiting (green lines) relations were assumed to be quantified by the following Hill functions, respectively,

$$f_{activation}=\frac{a{\left[L\right]}^{n}}{1+a{\left[L\right]}^{n}},f_{inhibition}=\frac{1}{1+a{\left[L\right]}^{n}}$$.

The Hill coefficient *n* mathematically determines the slope of the sigmoid curve (Supplementary Figure 1), and biologically defines the interactional cooperativity. Quantitative studies on signal transduction systems have revealed that switch-like and sigmoidal input/output relationships are common in cell signaling [51]. For example, the multistep binding of oxygen to hemoglobin [52], the binding of transcriptional factors to multiple DNA binding sites and priming in multisite phosphorylation [53] are known to exhibit switch-like and sigmoidal input-output relationships. Cooperativity has been demonstrated to account for the sigmoidal curve, and the mechanisms capable of creating a switch-like response have been discussed [51]. The Hill coefficient *n* that determines the slope of the sigmoid curve (Supplementary Figure 1), can quantitatively define the cooperativity. If n<1, the system exhibits negative cooperativity; if n=1, the system exhibits no cooperativity; if n>1, the system exhibits positive cooperativity; if 1<n<3, the sigmoidal curve can barely represent a switch-like behavior; if n≥3, the sigmoidal curve represent a switch-like behavior; if n≥10, the equations correspond to Boolean functions (Supplementary Figure 1). Moreover, quantitative studies of signal transduction systems—for example, cell cycle regulation [51], MAPK pathways [51], Ras activation [54] and Notch signaling [55]—have shown that Hill coefficient n≥3. Thus, the parameter range 3≤n≤10 was used in the working model.

As for parameter *a*, it biologically describes the kinetic properties of each component *L* in regulating the production of *x*. Mathematically, *a* defines a threshold at which the activation/concentration of *x* is half its maximal value. The deductive process of *a* is listed below.

Because the concentration/activity of each component *x* was normalized to the range from 0 to 1, we assumed that when [L] 0, then $f{\left(x\right)}_{activation}\approx 0,f{\left(x\right)}_{inhibition}\approx 0$; when [L]≈1, then $f{\left(x\right)}_{activation}\approx 1,f{\left(x\right)}_{inhibition}\approx 0$; and when [L]≈1/2, then $f{\left(x\right)}_{activation}\approx f{\left(x\right)}_{inhibition}\approx 1/2$. Thus, we deduced that $a\approx 2^{n}$.

Some studies have showed that Hill coefficients might vary in different cell types [51]. Thus, the Hill coefficients $n_{i}(n_{i}=3,4,5,10;i=1,2,\ldots ,215)$ and $a_{i}\approx 2^{n_{i}}$ were used in the current model. The invariant 8 attractors (Supplementary Table S4–S9) and 14 saddle points (Supplementary Table S10–S13) under parameter $n_{i}(n_{i}=3,4,5,10;i=1,2,\ldots ,215)$ and $a_{i}\approx 2^{n_{i}}$ were found. In addition, the random parameter $n_{i}(3\leq n_{i}\leq 10;i=1,2,\ldots ,215)$ and $a_{i}=2^{n_{i}}$ were checked, and the 8 attractors and 14 saddle points (Supplementary Table S14–S15) were still invariant under random parameter $n_{i}(3\leq n_{i}\leq 10;i=1,2,\ldots ,215)$ and $a_{i}=2^{n_{i}}$.

In some cases, the signal transduction process may exhibit a hyperbolic response or gradual activation/inhibition rather than a switch-like response. The hyperbolic response indicats that there is no or weak cooperativity, suggesting the Hill coefficient 1<n<3. Thus, the random parameters $n_{i}(1\leq n_{i}\leq 10;i=1,2,\ldots ,215)$ and $a_{i}=2^{n_{i}}$ were also checked in the working model. The 8 attractors and 13 saddle points (Supplementary Table S16–S17) were found still to be invariant under the random parameter $n_{i}(1\leq n_{i}\leq 10;i=1,2,\ldots ,215)$ and $a_{i}=2^{n_{i}}$. Several theoretical [56, 57] and experimental [58, 59] studies also have found that, in variations of biochemical parameters, the key processes of specific intracellular networks still exhibit a robust behavior.

Moreover, similar attractors were obtained via a Boolean approach within the same network (Supplementary Table S18). As shown in these results, the primary properties of the working network are mainly constrained by the network structure rather than by specific parameters. The parameters used to produce the figures in this study are as follows: $n_{i}=3,a_{i}=10,\tau_{j}=1,i=1,\ldots ,215,j=1,\ldots ,48$.

### Attractor caculation

First, through normalization, all biologically possible values of variables were confined to the interval [0,1]. Then, we chose random initial conditions and used the following two independent algorithms, the fixed-point iteration algorithm and the Newton iteration algorithm, to calculate attractors for the nonlinear dynamical system.

$$\frac{dx(t)}{dt}=f(x)$$

(1) Fixed-point iteration algorithm:

Firstly, we generated a random vector $x_{0}$; then, according to the dynamical systems, we iterated $x_{0}$ using $x_{i+1}=x_{i}+\Delta t\times f(x_{i})$; after a considerable number of iterations, we judged its convergence by $\left|x_{i+1}-x_{i}\right|<\varepsilon ,\left|f(x_{i})\right|<\delta$. Last, we recorded $x_{0}$, which meet the definition of being an attractor. The above calculations were repeated to find other attractors.

(2) Newton iteration algorithm:

We solved the nonlinear function f(x)=0 using Newton's method. We obtained fixed points using this algorithm. Eigenvalues linearized around a fixed point were used to determine whether the fixed points were stable or unstable. If the set of real eigenvalues for the system were uniformly negative, the fixed point was regarded as a stable point/point attractor. If the set of real eigenvalues for the system had both positive and negative eigenvalues, the fixed point was regarded as an unstable saddle point. Point attractors obtained by the Newton iteration algorithm were consistent with the results obtained by the fixed-point iteration algorithm.

It is practically impossible to enumerate all of the possible attractors of such a high-dimensional nonlinear dynamic system. We sampled enough times to confirm that there were at least 8 attractors. As shown in Supplementary Table S6, when the calculations were repeated 40000 times, we obtained 8 attractors (point attractors and cyclic attractors). When the calculations were repeated 40,000,000 or more times, we still obtained 8 attractors. Thus, we concluded that there were at least 8 attractors.

### Saddle point caculation

Using Newton's method to solve the nonlinear function f(x)=0, we obtain fixed points for the system $\frac{dx(t)}{dt}=f(x)$, which included both unstable and stable saddle points. We used two methods to determine unstable saddle points. First, as mentioned above, eigenvalues can be used to determine whether a fixed point is stable or unstable. The second method is that we perturbed the fixed point in the system. A stable point can be perturbed in any dimension yet eventually return to its original location and remain there. A saddle point will roll away from its original location and roll into its connecting stable points/attractors under perturbation. Saddle points calculated by the two independent methods are the same. In addition, through perturbation on saddle points, we were able to determine their connecting attractors and identify possible transition routes among attractors.

## RESULTS

### Construction of the core endogenous network of gastric cancer (GC)

The core endogenous network of GC was constructed according to the endogenous network hypothesis. (The biological assumptions and methods are provided in the Methods section and Text S1. Supporting method.) In this network, GC was described by a group of essential functional modules, and the functional status of each module was regulated by a group of key endogenous factors (Table 1). The endogenous factors and their activation/up-regulation and inhibition/down-regulation relations were drawn from documented signalling transduction pathways and gene transcription network in the PubMed literature database (including 275 references; details are provided in Text S1. Supporting method and Supplementary Table S1). These endogenous factors and their interactions constituted a closed-loop network, encompassing 8 functional modules, 48 endogenous factors and 215 connections. The working network aimed to present the core endogenous regulatory mechanism of gastric epithelium and analyze how these endogenous factors cooperate and collectively function at a systematic level. Because the key endogenous factors and their interaction relations have been demonstrated to be conserved, the core endogenous network of GC can be reproduced according to the hypothesis.

**Table 1:** Modules and components in the core endogenous molecular network for gastric epithelium

Essential modules	Key components
**Cell cycle**	Rb, Cyclin D-CDK4/6, Cyclin E-CDK2, Myc, E2F, p21, p27,
**Apoptosis**	Caspase 3/7, Cytochrome *c*/Caspase 9/Apaf-1, Caspase 8/10, XIAP, Bcl-2, Bcl-*X_L_*, Bid, Bad, Bax/Bak
**Growth factors**	EGF/EGFR, IGF/IGFR, HGF/Met, VEGF/VEGFR, Gastrin, Ras, PI3K-AKT, ERK, JNK, p38, PTEN, MKP
**Cell adhesion**	Integrin, E-cadherin, Zeb1/2
**Inflammation**	p53, HIF, NF-κB, iκB, TNF-α, IL-10, IL-1, IL-8, Fas, TGF-β, IFN-γ, STAT3
**Angiogenesis**	VEGF/VEGFR, COX-2
**Metabolism**	GSK3β, HIF, Akt
**Gastric differentiation**	Sox2, Shh, β-catenin, Cdx2

It should be noted that the current endogenous network of GC is vastly simplified under our framework. A detailed molecular endogenous network will contain a vast number of endogenous factors; at present, the modeling of all interactions remains beyond our capabilities [60]. Therefore, only essential functional modules and key components were included in the network. Functional modules or components that were not presented in the current work may exert influence or be influenced by the working network. The working network is one of the simplest versions and can be expanded or revised to accommodate those effects. Nevertheless, we will demonstrate that the simplest version may characterize the main features of normal gastric epithelium and GC at the modular and molecular levels. In addition, we provide new predictions that need to be tested by new experimental data.

### Eight attractors were identified

**Figure 2:**
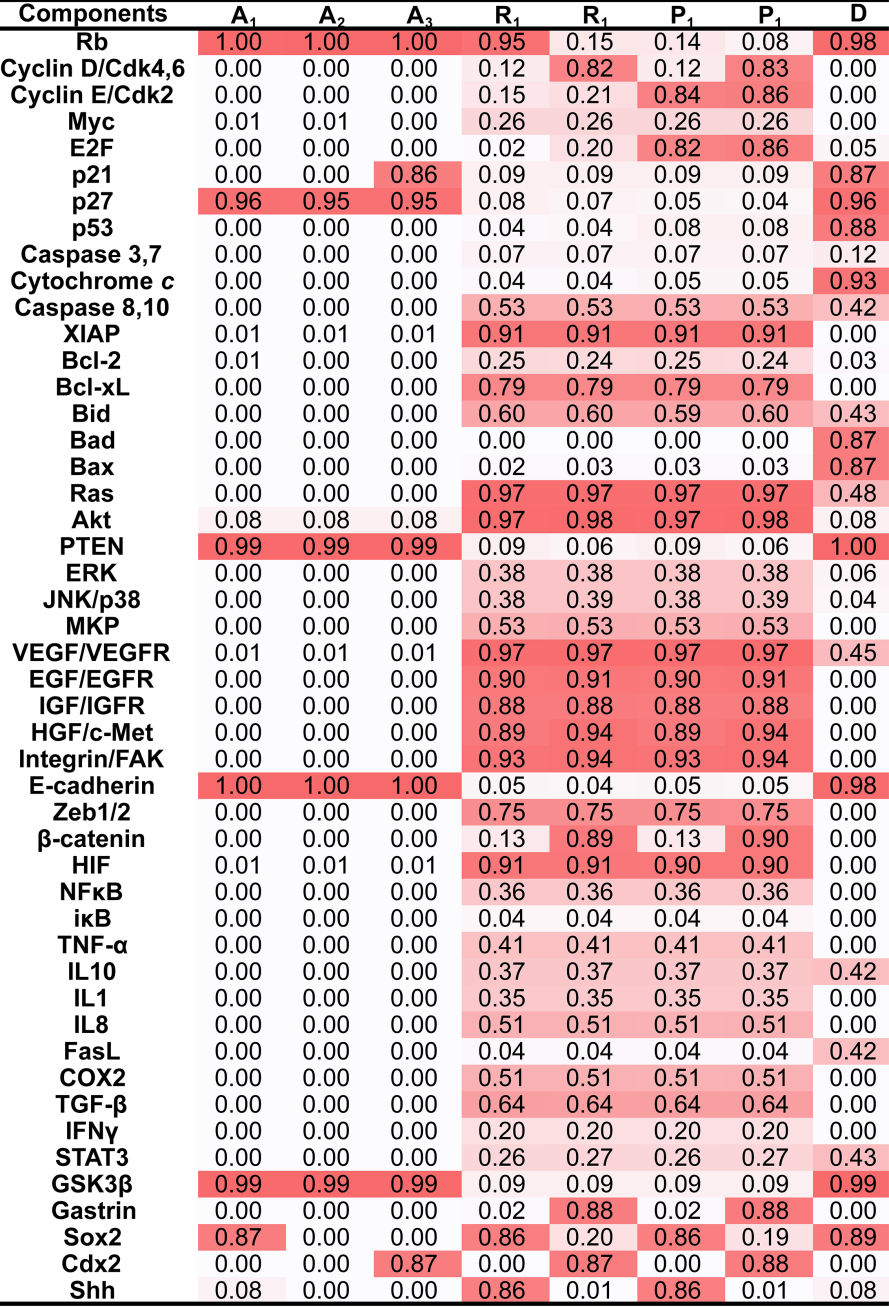
Attractors in the working network Eight attractors were identified in the endogenous molecular network for GC. Each attractor was characterized by the relative activities of all components, which were normalized to the range from 0 to 1, with 0 (white) denoting minimal activity and 1 (Red) denoting maximal activity. According to the component activity level in different functional modules, the eight attractors were classified into 4 cell fates including cell cycle arrest (A_1_, A_2_ and A_3_), cell death D., stress response (R_1_ and R_2_) and proliferation (P_1_ and P_2_). Each column represents a component and each row represents an attractor. A_1_, A_2_, A_3_, R_1_, R_2_, P_1_ and P_2_ are point attractors, whereas D is a cyclic attractor. The dynamical trajectories of caspase 8/10, Bid, Fas, VEGF/VEGFR, IL10, Ras and STAT3 are shown in Supplementary Figure 2.

We have provided a general framework to quantify the core endogenous molecular network [22, 47, 48] (the complete list of model ODEs being provided in Supplementary Table S1) and transformed it into a nonlinear dynamic system, which contained some attractors underlying the endogenous molecular network. Two independent algorithms were used to calculate attractors; these algorithms yield consistent results: at least 8 local attractors were found in the network (Figure 2).

**Figure 3:**
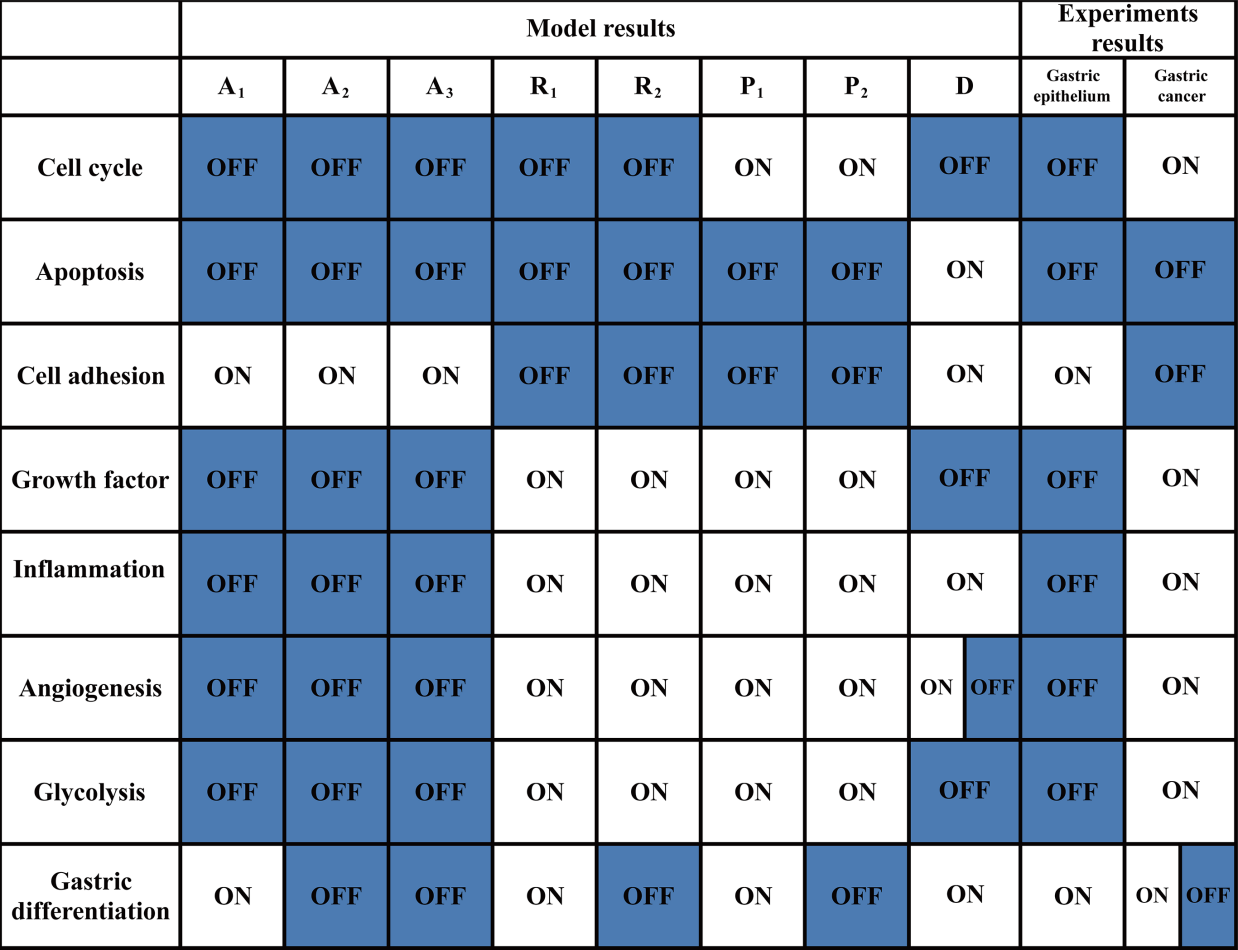
Functional status of modules from the model and experimental results The functional statuses of the modules for each attractor from the endogenous network are listed in column 2—9. A_1_–A_3_: cell cycle arrest attractor; P_1_–P_2_: proliferation attractor; D: cell death attractor; R_1_–R_2_: stress response attractor. D is a cyclic attractor from which some components activities, such as VEGF/VEGFR, oscillated (Supplementary Figure 1). Thus, the functional status of angiogenesis module could oscillate between ON and OFF. The functional statuses of modules in normal gastric epithelium and GC based on experimental and clinical data are listed in column 10–11. [14, 61] N/A indicates that the functional module status is experimentally inconclusive. The clinical samples of GC show either gastric or non-gastric (mainly intestinal) phenotype at both cellular and histological level. Thus, the functional statuses of the gastric differentiation module in GC are either ON or OFF in GC.

Each attractor potentially corresponded to a specific cellular phenotype with significant biological functions. Attractors are characterized by the relative activities/concentrations of components. According to the components activation or expression level of a component, the functional status (i.e., “ON” or “OFF”) of the modules in each attractor can be determined. We then summarized the status of functional modules in each attractor (Figure 3). The 8 attractors were classified into the following 4 unique cell fates based on the status of function modules: cell cycle arrest (A_1_–A_3_), proliferation (P_1_ and P_2_), cell death (D), and stress response (R_1_ and R_2_) (Figure 2). Point attractors A_1_–A_3_ with relatively high Rb and p27 activities manifested an “OFF” cell cycle and were defined as possessing a cell fate of cell cycle arrest. Cyclic attractor D with relatively high activities of pro-apoptosis factors and low activities of anti-apoptosis factors indicated that apoptosis is “ON”, and the cell fate was defined to be cell death. Point attractors R_1_ and R_2_ manifested relatively high HIF, NF-κB and inflammatory factor/receptor activities, suggesting that the stress response is “ON.” The cell fate of this class was defined as stress response. Point attractors P_1_ and P_2_ manifested relatively high cyclin E/Cdk2 and E2F activities and low Rb and p27 activities, indicating an “ON” cell cycle. The cell fate of this class was defined as proliferation. In summary, these attractors may represent specific cellular phenotypes.

### Validation of the endogenous network at the modular and molecular levels

The working network was validated by the main features of gastric epithelium and GC at the modular and molecular levels. Normal gastric epithelium was assumed mainly to consist of normal differentiated epithelial cells, and GC tissue was assumed mainly to include cancer cells. The statuses of the functional modules were summarized, including cell cycle, apoptosis, inflammation, angiogenesis, metabolism, cell adhesion and gastric differentiation, in normal gastric epithelium and GC using clinical and experimental data (Figure 3) [14, 61]. The normal gastric epithelium and GC exhibited opposing statuses for some essential modules. The attractors A_1_ and P_1_–P_2_ were specifically consistent with normal gastric epithelium and GC, respectively, at modular level. In summary, we preliminarily concluded that A_1_ and P_1_–P_2_ characterize normal gastric epithelium and GC, respectively.

The preliminary conclusion was then validated with both low- and high-throughput data at the molecular level in normal gastric epithelium and GC. First, we collected relative changes in the expression or activity of each component from GC and adjacent, noncancerous gastric tissues from low-throughput experimental data (Supplementary Table S2). For the low-throughput data, gene expression was detected by real-time reverse transcription quantitative PCR (RT-qPCR), whereas protein concentration and activity status were detected by immunohistochemistry and western blotting. For example, the activation statuses of cytokines, such as EGF, TNF-α and gastrin, were mainly regulated at the transcription level, which were checked by RT-qPCR data; whereas the activation statuses of signaling proteins, such as PI3K/Akt, ERK, JNK and p38, were mainly regulated by phosphorylation, which were checked by immunohistochemistry and western blot data.

**Figure 4:**
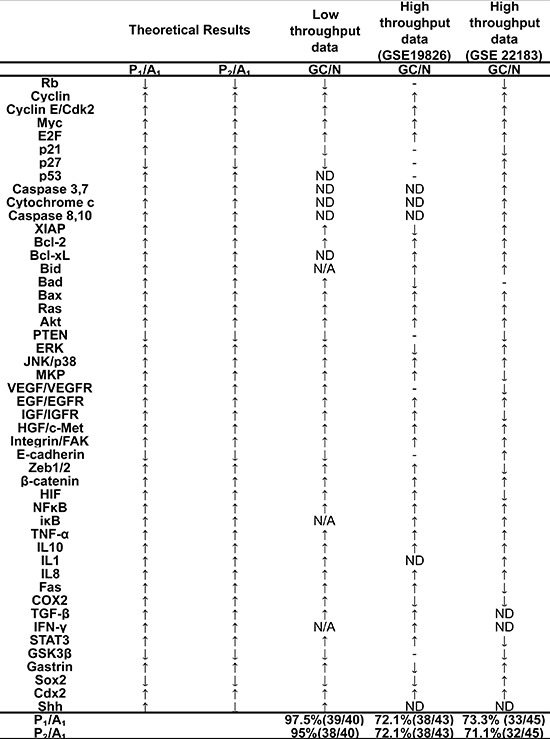
Comparing the modeling results and experimental data of the relative change of components If the ratios of one component between two attractors (for example, P_1_ to A_1_) are greater than 1, we define the component activity as increased; otherwise, the component activity is decreased. Relative changes in expression or activities of components from normal gastric epithelium to GC are summarized from low and high throughput data (in column 3–5, detail references in Supporting Supplementary Table S2 and S3). The comparison indicates that 97.5% of modeling results P_1_/A_1_ are consistent with low-throughput experimental data and that 72.1% and 73.3% are consistent with high-throughput data, respectively; and for modeling results P_2_/A_1_, that 95%, 72.1% and 71.1% are consistent with low and high-throughput data, respectively. GC, gastric cancer; N, normal gastric epithelium; ↑, increased component activity or expression; ↓, decreased component activity or expression; ↓, non-significant or uncertain variation in component activity or expression; N/A, no experimental data; ND, non-determined variation in component expression.

Next, we summarized the relative transcriptional changes of each component from sporadic gastric adenocarcinoma samples and normal gastric mucosa samples from high-throughput data (Supplementary Table S3). The relative changes of the components in P_1_ and P_2_ were compared with A_1_ (Supplementary Table S2 and S3). The comparison indicated that 97.5% of modeling results P_1_/A_1_ were consistent with low-throughput experimental data (including 74 references, Figure 4 and Supplementary Table S2) and that 72.1% and 73.3% were consistent with high-throughput data, respectively (Figure 4 and Supplementary Table S3); and for modeling results P_2_/A_1_, that 95%, 72.1% and 71.1% were consistent with low and high-throughput data (Figure 4, Supplementary Table S2 and S3, GEO ID: GSE 19826, GSE 22183), respectively. For high throughput data, with the exception of checking the gene expression of the components in the working network, we also checked the gene expression of downstream nodes of some signaling pathways, including Ras/MAPK, VEGF, NF-κB and Wnt/β-catenin signaling (presented in Supplementary Table S19). The comparison indicated that 67% (GSE 19826) and 66.7% (GSE 22183) of the relative gene expression changes of downstream nodes predicted by the modeling results were consistent with high-throughput data. Considering the heterogeneity of cancer [10] and the intrinsic experimental error [62], the accuracy rates suggested good consistency.

The consistency of the model results and the experimental data at the modular and molecular levels support the notion that the working network may characterize the main features of the normal gastric epithelial and GC phenotypes, respectively. The result is quite interesting because the molecular components and interactions in the network were obtained independent from microarray data and because the attractors closely agreed with those data. It should be noted that, given the heterogeneity characteristic of cancer, intrinsic experimental error, assumption about parameters and incomplete working network, it was neither sufficient nor necessary to adjust the model to exactly fit existing experimental data, such as fold changes.

### Specific positive feedback loops maintain the intestinal and gastric types of GC cell attractors

One important property of a nonlinear dynamic system is that the multistability of the system is produced and maintained by key components and their interactions [63, 64]. Because the endogenous molecular network is quantified by a nonlinear dynamic system, its properties can be discussed here. The working model revealed that underlying positive feedback loops (PFLs) are responsible for maintaining the heterogeneous phenotypes of GC.

**Figure 5:**
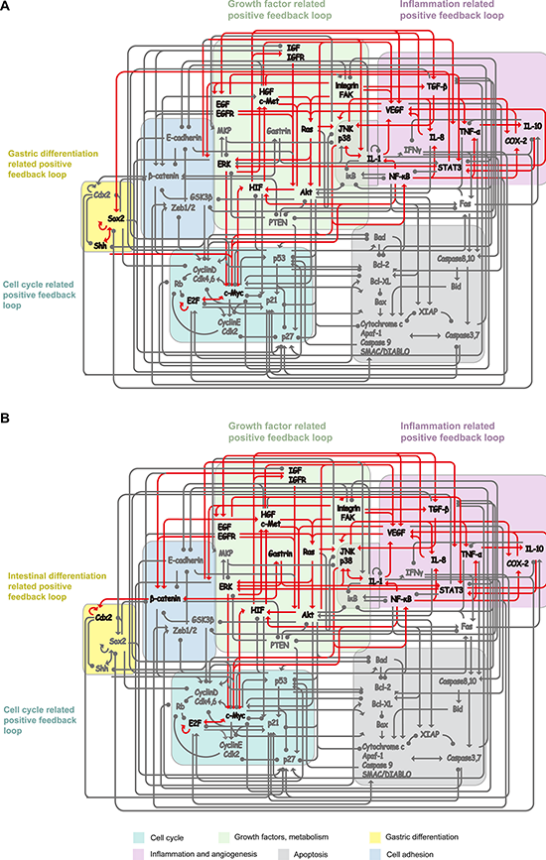
Positive feedback loops (PFLs) maintain heterogeneous GC cell attractors **A.** Gastric type GC cell attractor is maintained by four positive feedback loops: growth factor related PFL (RTKs, Ras, Akt, MAPKs and HIF); cell cycle related PFL (Myc and E2F); inflammation related PFL (NF-κB, TNF-α, IL-1, IL-8 and IL-10)); gastric differentiation related PFL (Sox2 and Shh). **B.** Intestinal type GC cell attractor was maintained by growth factor related PFL, cell cycle related PFL; inflammation related PFL and intestinal differentiation related PFL (Gastrin, β-catenin and Cdx2).

The working network revealed that the GC cell attractor P_1_ is maintained by four positive feedback loops: growth-factor-related, cell-cycle-related, inflammation-related and gastric-differentiation-related PFLs (Figure 5A). In contrast, the GC cell attractor P_2_ was maintained by growth-factor-related, cell-cycle-related, inflammation-related and intestinal-differentiation-related PFLs (Figure 5B).

Both P_1_ and P_2_ were maintained by the growth-factor-related, cell-cycle-related and inflammation-related PFL. The growth-factor-related PFL included receptor tyrosine kinases (RTKs)-Ras, Akt and mitogen-activated protein kinases (MAPK, including ERK, JNK and p38). RTKs are cell surface receptors for many growth factors, including EGFR, IGFR, Met and VEGFR [65]. RTK activation may lead to the activation of downstream signal transduction pathways, such as the MAPK signaling cascade, and the activation of signal transduction pathways can change gene expression and activate RTKs and, subsequently, establish a growth-factor-related PFL [65, 66]. Inflammatory cytokines (IL-1, IL-8, TNF-α) and NF-κB establish an inflammation-related PFL [67, 68]. Inflammatory cytokines, IL-1, IL-8 and TNF-α, can activate NF-κB through IKK-iκB-signaling or JNK-signaling pathways. Activated NF-κB in epithelial cells can produce cytokines, such as IL-1 and IL-8, thus constituting an inflammation-related PFL. The transcription factors Myc and E2F can induce the expression of each other, and E2F also can transcriptionally activate its own genes E2F-1 and E2F-2, thus establishing a cell-cycle-related PFL [69, 70].

The difference between the P_1_ and P_2_ attractors is that P_1_ is also maintained by the Sox2-Shh PFL, whereas P_2_ is also maintained by the gastrin-β-catenin and the Cdx2 autoregulatory PFLs. Expression of the Sox2 gene can be regulated by the Gli2 transcription factor, a downstream effector of Shh signaling, and Shh is also a target of Sox2, thus establishing a PFL [71]. Gastrin was identified as a target of the β-catenin/TCF-4 growth-signaling pathway, and gastrin also stabilizes β-catenin protein and activates β-catenin-mediated transcription, thus establishing a PFL [72]. The gastrin-β-catenin positive loop can activate the Cdx2 autoregulatory loop, which plays a critical role in intestinal differentiation [34].

The working network revealed that different positive loops are responsible for maintaining heterogeneous GC cell attractors. The Sox2-Shh PFL may be responsible for maintaining the gastric-type GC cell attractor P_1_. Biologically, Sox2 exerted a dominant effect on the maintenance of the gastric epithelial phenotype [73] and is involved in the regulation of gastric-specific genes, such as pepsinogen and Muc5ac [74, 75]. Shh target genes modulate gastric parietal cell functions, such as acid secretion [76]. The activated Sox2-Shh PFL may influence other functional module statuses, such as modulating the cell cycle and inducing differentiation of gastric epithelial cells [73]. The working network also showed that the Gastrin-Wnt/β-catenin and Cdx2 autoregulatory PFLs might be responsible for maintaining intestinal-type GC cell attractor P_2_. Cdx2 and Wnt/β-catenin signaling play pivotal roles in intestinal development [77, 78] and are abnormally expressed/activated in intestinalized gastric mucosa and intestinal-type GC [79, 80]. Clinical data also revealed that Sox2 was up regulated in gastric-type GC, whereas Cdx2 was up regulated in intestinal-type GC [81]. These experimental data indicate that gastric- and intestinal-type GC cell phenotypes are maintained by the Sox2-Shh and Gastrin-Wnt/β-catenin-Cdx2 loops, respectively. The activated Gastrin-Wnt/β-catenin-Cdx2 loops may also affect the statuses of other functional modules, such as modulating the cell cycle and inducing the transdifferentiation of gastric epithelial cells [34, 79, 82].

Regulating the two distinct positive feedback loops may control the transition between the two heterogeneous cancer attractors. The working model indicated that intestinal-type attractor can be induced to the gastric-type by consistently expressing Sox2 (Supplementary Figure 3A). However, gastric-type GC can be induced to the intestinal-type merely by consistently activating Cdx2 and inhibiting Sox2 simultaneously (Supplementary Figure 3B). Biologically, PFLs can be activated/inhibited by activating/inhibiting proteins in the loops directly, or by modulating other regulatory molecules that affect the PFL indirectly. Small interfering RNA (siRNA) transfection and Cre-Lox recombination technologies can be used to inhibit and induce the expression of proteins in the loops; thus, we can use these technologies to testify these predictions. In short, the network posits a potential regulatory mechanism that controls the transition between two heterogeneous cancer cell phenotypes transition.

### Heterogeneous dynamic routes of GC progression

**Figure 6:**
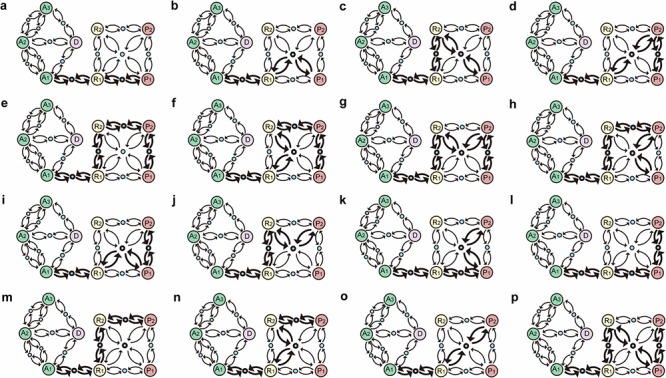
Inherent evolutional routes from normal gastric epithelial cell attractor A_1_ to GC cell attractors P_1_ and P_2_ Sixteen routes (a–p) from A_1_ to P_1_ or P_2_ were identified in the functional landscape of the core endogenous molecular network for gastric epithelium. Large circles represent attractors indicating 8 cellular phenotypes. Blue circles represent saddle points, which are unstable equilibrium states. Arrows denote where a transition likely occurs between 2 attractors. A_1_, A_2_ and A_3_, cell cycle arrest; D, cell death; R_1_ and R_2_, stress response; P_1_ and P_2_, proliferation.

Another property of nonlinear dynamic system is that the multistable system is thought to be involved in the generation of switch-like biological process. In response to a transient perturbation, the system may flip back and forth between attractors. The saddle point, a particular unstable equilibrium, of a multistable system is highly relevant to understand the global behavior of the transition process between attractors. One feature of a saddle point is that it is like a mountain pass connecting neighboring valleys and reveals the possible transition routes between attractors [83]. Fourteen saddle points were obtained in the model, which were robust against reasonable parameters as well as random parameters. The attractors and saddle points depicted a schematic functional landscape of the working network (Supplementary Figure 4). The functional landscape has provided a useful framework to consider non-genetic changes that occur during cancer progression [11]. From this perspective, non-genetic perturbations, such as fluctuations in gene expression or environmental stimuli, may drive transition between distinct attractors in the given landscape. The functional landscape of the working network revealed 16 transition routes from the normal gastric epithelial cell attractor A_1_ to either of the GC cell attractors P_1_ or P_2_ (Figure 6).

Another feature of saddle point is that it captures the intermediate phase of the transition process. Transition routes that connected the normal gastric epithelial cell attractor A_1_, stress response attractors R_1_ or R_2_, the GC cell attractors P_1_ or P_2_ and the saddle points among them may reveal definite dynamic routes of GC cell progression. According to the component activation pattern in these transition routes, these transition routes characterized the main features of GC cell progression, including increased cell proliferation, enhanced inflammation, evasion of apoptosis, induced angiogenesis and activated invasiveness [41, 84].

**Figure 7:**
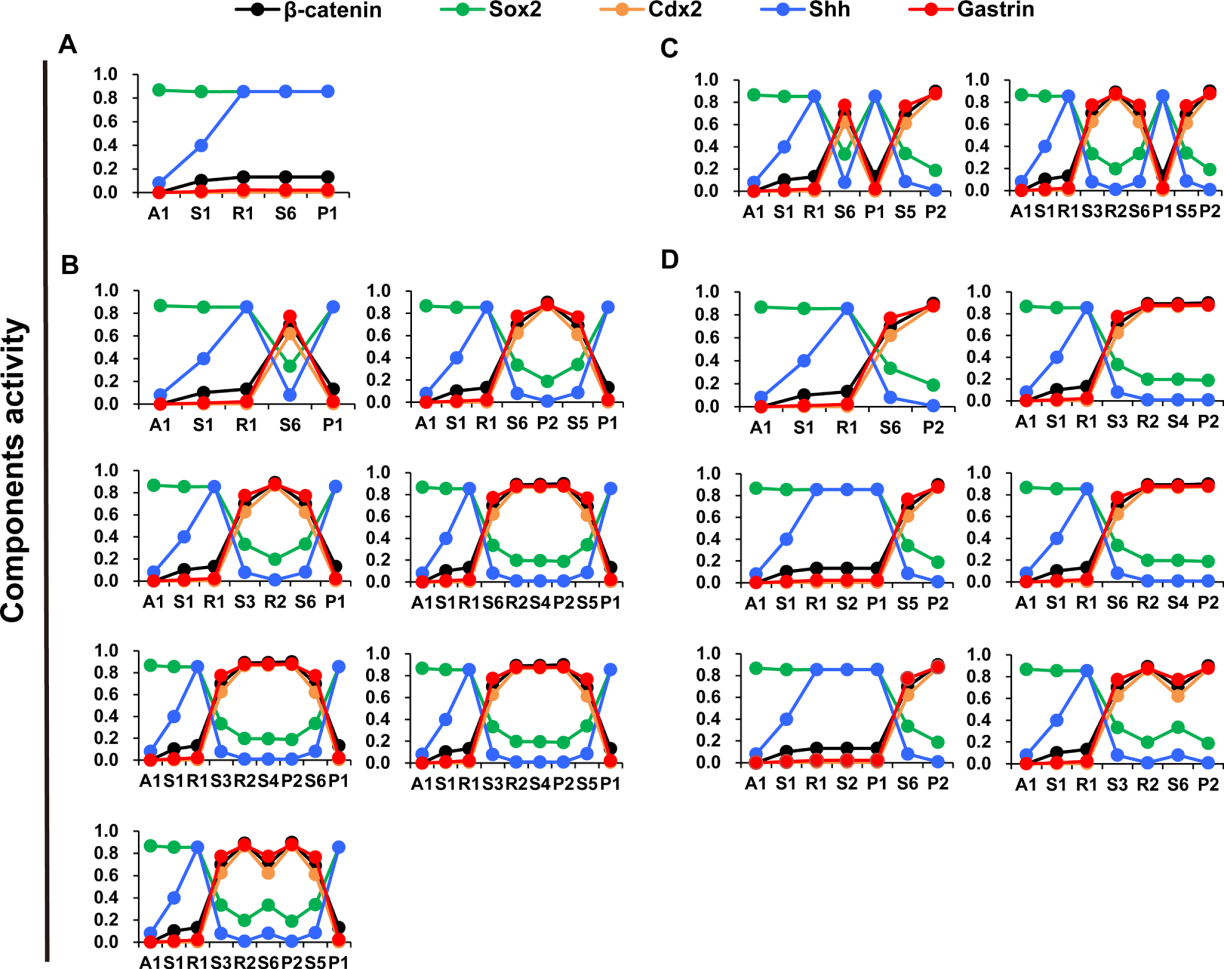
Dynamics of differentiation components in the routes of GC progression **A–D.** shows components activity dynamics in 16 possible inherent evolutional routes from normal gastric epithelial cell attractor A_1_ to GC cell attractors P_1_ and P_2_. The dynamic process of certain critical regulators exhibit 4 major patterns of GC progression: **A–D**. A_1_, A_2_ and A_3_, cell cycle arrest; D, cell death; R_1_ and R_2_, stress response; P_1_ and P_2_, proliferation; S1–S14, saddle points.

The 16 transition routes were determined by the connections of attractors and saddle points, which revealed heterogeneous activation patterns of particular components. According to the combined activation pattern of β-catenin, Cdx2, gastrin, Sox2 and Shh, the 16 transition routes could be classified into 4 definite heterogeneous routes of GC cell progression: (1) Sox2 and Shh activities are high, whereas β-catenin, Cdx2 and gastrin activities are low (Figure 7A); (2) Sox2 and Shh activities decrease and then increase, while β-catenin, Cdx2 and gastrin activities increase and then decrease (Figure 7B); (3) Sox2 and Shh activities oscillate and then decrease, whereas β-catenin, Cdx2 and gastrin activities oscillate and then increase (Figure 7C); and (4) Sox2 and Shh activities gradually decrease, whereas β-catenin, Cdx2 and gastrin activities increase (Figure 7D). The heterogeneous activation patterns resulted from the mutually inhibitory natures of the Sox2-Shh and Gastrin-Wnt/β-catenin-Cdx2 PFLs (Supplementary Figure 3). When components in the two PFL are expressed at appropriate levels, the Sox2-Shh or Gastrin-Wnt/β-catenin-Cdx2 PFL may switch on/off; then the system may tend to settle upon either gastric- or intestinal-type attractors.

The Sox2-Shh and Gastrin-Wnt/β-catenin-Cdx2 PFLs not only influenced gastric differentiation but also mutually interacted with other functional modules, such as cell cycle, apoptosis, metabolism and angiogenesis. Some components of the cell cycle module also exhibited heterogeneous patterns during GC cell progression (Supplementary Figure 5). The cell state transitions between R_1_, R_2_, P_1_ and P_2_ may affect the cellular phenotype during GC cells progression, such as the proliferative capacity and morphology of cancer cells. Thus, the 16 transition routes indicated that GC cell progression is heterogeneous, which is revealed by the dynamic behaviors of individual key molecular components. In addition, the functional landscape indicates that cells in GC can be in cancer attractors P_1_ and P_2_ and saddle points. Thus, they may exhibit gastric, intestinal, or even intermediate cancer phenotypes, respectively, thereby providing a potential mechanism for then co-occurrence of intestinal- and gastric-type cancer cells in clinical samples [85].

## DISCUSSION

### Specific positive feedback loops contribute to heterogeneity within GC

Our work suggests that two mechanisms can contribute to the heterogeneous phenotypes of GC cells. First, the nonlinear biochemical interactions among endogenous factors formed two kinds of PFLs, Sox2-Shh and Gastrin-Wnt/β-catenin-Cdx2, that generate and maintain the gastric and intestinal types of GC cells, respectively. The PFLs confer stability and robustness of heterogeneous phenotypes upon perturbations. For example, *Helicobacter pylori* is a strong stimuli of inducing intestinal transformation of gastric epithelial cells [86]. Following *H. pylori* eradiation in patients, intestinal-type epithelial cells do not readily revert because these intestinalized cells may be maintained by PFLs activated by the stimulation of *H. pylori*. Without exogenous stimulation, the PFLs are still activated.

In addition, some experiments have indicated that the knockdown, knockout or overexpression of critical components of positive feedback loops change cell phenotypes. For example, the *Shh* mutant mice have been shown to exhibit intestinal transformation of the gastric epithelium [87], which indicates that breaking the gastric-phenotype-related PFL Sox2-Shh changes the gastric phenotype. Moreover, ectopic Sox2 expression in the primitive gut has been shown to redirect the developmental fate of the intestinal epithelium toward a gastric-like phenotype [73], suggesting that the gastric-differentiation-related regulatory network may be activated by activating Sox2. This experimental result was also consistent with the modeling result that consistently activating Sox2 can activate Sox2-Shh PFL and then induce transformation of the intestinal-type attractor to gastric-type attractor (Supplementary Figure 3A).

As regards the intestinal-differentiation-related Cdx2-Wnt/β-catenin-gastrin feedback loops, there are evidences indicating that *H. pylori* can deregulate Wnt/β-catenin signal [88], induce Cdx2 expression and inhibit Sox2 expression in gastric epithelial cells [35]. These studies indicate that *H. pylori* infiltration may activate intestinal-differentiation-related PFL and inhibit gastric-differentiation-related PFL of GC cells, thus inducing the intestinal transformation of the gastric epithelium. These are consistent with our modeling results, which also indicated that the gastric-type attractor can be transformed to the intestinal-type attractor by consistently activating the Wnt/β-catenin or Cdx2 and inhibiting the Sox2 simultaneously (Supplementary Figure 3B). Additionally, frequent somatic mutations of the *APC* and β-catenin genes were found only in intestinal-type GC [89]. Increased β-catenin mRNA levels were also significantly more frequently occurring in intestinal-type GC compared to the diffuse-type GC [90]. These studies indicate that abnormal activation of the intestinal-differentiation-related PFL via genetic mutation and aberrant expression of related proteins may be the cause of the intestinal transformation of the gastric epithelium.

The working model also indicated that perturbation of cancer related PFLs can influence the cancer-like phenotype. The modeling results showed that persistently inhibiting phosphatidylinositol 3-kinase (PI3K)/Akt activity induce the switch of GC cell attractors to cell-cycle-arrest and apoptosis-like attractor (Supplementary Figure 6), indicating that PI3K/Akt is a critical component of cancer-related PFLs and persistently inhibiting the PI3K/Akt signaling pathway may increase the susceptibility of cancer cells to apoptosis. Intriguingly, using PI3K/Akt pathway inhibitors in gastric cancer cell lines caused cell cycle arrest, enhanced sensitivity to apoptosis and attenuated the chemotherapeutic resistance of GC cells [91–94].

### Different transition routes contribute to heterogeneity within GC

The properties of the nonlinear dynamic system revealed that the transition between normal gastric epithelial cell attractor and GC cell attractors can occur along no fewer than 16 transition routes characterized by the dynamic behaviors of individual key molecular components, thereby suggesting that the GC cells progression routes are heterogeneous. The working model identified that the normal gastric epithelial cell attractor A_1_ needs to go through at least one intermediate attractor to arrive at the GC cell attractors P_1_ or P_2_. For example, A_1_ may go through attractor R_1_ to arrive at P_1_, but A_1_ may also go through R_1_ and then R_2_ to arrive at P_1_. Moreover, in some cases, more than one saddle point was found between two attractors, which indicated that there were different transition routes or mechanisms between two attractors. Thus, there were 16 total different potential transition routes from the normal gastric epithelial cell attractor A_1_ to the GC cell attractor P_1_ or P_2_ were found.

The 16 transition routes of GC cell progression can be classified into 4 patterns according to the dynamic behaviors of individual key molecular components. One pattern of GC cell progression was found consistent with the pathogenic process of intestinal-type GC. Pelayo Correa proposed that intestinal-type GC passes through a sequence precancerous lesions including inflammation, atrophy, intestinal metaplasia (IM) and dysplasia [27, 29]. The expression levels of some key molecules of intestinal-type GC at different carcinogenesis stages were studied. For example, Cdx2 expression was activated in cells of IM, dysplasia and intestinal-type GC [95], whereas the gastric transcriptional factor Sox2 was down regulated in IM cells and intestinal-type GC cells [37, 96]. Moreover, the cytoplasmic and nuclear accumulation of β-catenin could be observed in premalignant lesions (atrophic gastritis and IM) [97], and increased β-catenin mRNA levels and mutational alterations of the APC and β-catenin gene are present in intestinal-type GC [90]. These independent clinical trajectory data were consistent with our modeling predictions and corresponded to one dynamic pattern of key molecules during intestinal-type GC cell progression (Figure 7D).

The routes also showed that activation of Cdx2 and β-catenin may not occur in the intermediate attractor R during intestinal-type GC cell progression, suggesting that IM may be not a necessary step for intestinal-type GC cell progression (Figure 7D). By contrast, diffuse-type GC arises without identifiable IM precursor lesions. Interestingly, some experiments have shown that diffuse-type GC includes both gastric- and intestinal-type GC cells, which suggested that IM might be independent with induction of intestinal-type GC cells [33]. Further experiments are required to validate this prediction.

The working model also predicted three other patterns of GC cell progression. For example, the gastrin-Wnt/β-catenin-Cdx2 PFL may not activate during some GC cells progressions (Figure 7A). Moreover, GC cells may switch between gastric- (P_1_) and intestinal-type (P_2_) attractors (Figure 7B and 7C), suggesting that under proper perturbation, intestinal-type GC cells may transform into gastric-type GC cells, and vice versa. In addition, because gastric epithelial cells may stay in different GC attractors, intermediate attractors or saddle points during GC cell progression, whole cancer tissues may exhibit different proportions of heterogeneous cancer cells and precancerous cells among patients. To further validate these predictions and clarify the dynamic molecular process of GC cells, new technology and experiments at the cellular level are required.

The adaptive landscape is useful to graphically depict the dynamic system and transition between attractors [98]. Fluctuation/noise in gene expression or other cellular processes as well as transient environmental stimuli/exogenous factors may shift the cell state in a given landscape, causing changes in cellular phenotypes [11]. Phenotypic switch via stochastic fluctuation has been observed and modeled in prokaryotic [78, 99] and eukaryotic [100] systems. Although the dynamics of biochemical factors representing the endogenous network can be described via a set of stochastic differential equations [99, 101], recent progress allows us to ignore the stochastic effect during first step of studying cancer cell progression [102, 103]. For example, theoretically, adding a stochastic term will not influence the deterministic results, such as the attractors and the saddle points. Moreover, recent progress in dynamic stochastic systems has provided a methodology to constructing the adaptive landscape and quantitatively analyzing the transition process based endogenous molecular network [103, 104].

### The endogenous molecular network hypothesis and current models of tumor heterogeneity

The endogenous molecular network includes both genetic information and biochemical interactions among endogenous factors. The nonlinear biochemical interactions among endogenous factors that establish particular structures, such as PFLs, generate and maintain heterogeneous tumor attractors. According to clonal evolution theory and CSC theory, three sources—genetic diversity, epigenetic modification, and the tumor microenvironment—contribute to tumor heterogeneity [10, 105]. It is affirmed that these three sources can be incorporated in the endogenous molecular network theory. Genetic mutation can be represented by removing or adding endogenous factors and the interactions among them to the endogenous network [39]. Epigenetic modification is commonly used to describe chromatin-based events, such as DNA methylation, histone modification, noncoding RNA and nucleosome location, which regulate DNA-templated processes, such ad gene expression [106]. Epigenetic variations are assumed to subtly enhance or weaken the interactions strengths of endogenous factors. For example, DNA methylation and histone modification may regulate gene expression via affect the kinetics of transcription-factor binding to DNA [107]. Microenvironmental stimuli correspond to the exogenous factors referenced in this article, which include biological, physical and chemical exogenous factors. Microenvironmental stimuli can directly or indirectly influence the endogenous network by causing genetic mutation and epigenetic variation and by activating/inhibiting signaling transduction or metabolic pathways. These three facets may strengthen or impair the loops that maintain distinct attractors. The CSC concept can also be discussed in the framework of endogenous networks and adaptive landscapes [38, 108]. The “CSC” and “non-CSC” phenotypes could serve as attractors in the endogenous network, and bidirectional conversion between the CSC and non-CSC phenotypes can occur under the control of endogenous and exogenous factors.

We can also explain some phenomena that are difficult to describe using current mechanisms posited for heterogeneity. For example, some studies have found that breast cancer adjacent cells are genetically divergent but phenotypically similar [18], which indicate that cells with different genetic mutations can exhibit similar phenotypes. On the other hand, in some cases, no critical mutations or epigenetic variations are found in cancer cells, despite their exhibiting cancer cell morphology and immunology [15–17]. Moreover, one study suggests that a small number of cells in patients with prolonged remissions of APL still harbor PML-RARα fusion genes but do not exhibit the APL phenotype [19]. These data suggest that cells with similar genetic mutations are nevertheless able to exhibit different phenotypes. According to our results, the working network generates attractors with different biological functions, which indicate that the same genome type with the same epigenetic modification and microenvironment can also generate heterogeneous cell phenotypes. Moreover, if the genetic mutation, epigenetic modification and microenvironment variation do not impair the feedback loops that maintain a specific phenotype in the network, they may only influence the stability of attractors; they do not cause attractors to disappear or induce cellular phenotype changes.

### The endogenous molecular network and other literature based networks

Other cancer networks based on signaling-pathway and transcriptional-regulation databases have also been proposed. Small-scale networks, such as the p53 network [109], MAPK network [110] and death receptor-mediated signaling network [111], have been proposed to study detailed key pathways and their dynamic behaviors related to human cancer. These pathways and functional modules do not work independently to promote carcinogenesis; rather, they closely cooperate through signaling crosstalk, transcriptional regulation and other forms of feedback mechanisms [112].

Some large-scale cancer networks were also constructed by data mining of comprehensive databases [113, 114]. Wang et al. [115] provided a cancer hallmark network framework that combined a literature-based cellular signaling and transcriptional network and genome sequencing data to predict clinical phenotype and better design patient treatment. The literature-based data also has been used in our network modeling. Some differences between the cancer hallmark network framework and the endogenous molecular network theory nevertheless remains. First, the cancer hallmark network is an open-loop network that considers comprehensive signaling pathways and interactions. However, based on studies of feedback control of intercellular signalling and transcriptional network [18, 19], one assumption of the working model is that the endogenous network is an autonomous and closed-loop network. The closed-loop network introduces feedback on each node in the network: the process input has an effect on the process output; the output is fed back as input to the process, thereby closing the loop. Second, the PFL of the cancer hallmark network is the loop formed by mutual activation of the mutation network and the survival network. In contrast, the PFLs in the endogenous molecular network are the loops formed by the signaling pathways and transcriptional relations among endogenous factors. The endogenous molecular network also can consider mutation effects. Genetic mutation can be represented by removing or adding endogenous factors and interactions among them in the endogenous network [39], thereby attenuating the loops that maintain the normal phenotype and strengthening the loops that maintain the malignant phenotype in the network, which may then result in phenotype change.

Interestingly, the endogenous molecular network showed some consistency with the cancer hallmark network. For example, the two networks showed good agreement with respect to signaling pathways and functional modules, such as cell proliferation/cell cycle, cell death, angiogenesis, differentiation, EMT/cell adhesion and metabolism. Moreover, Wang proposed that the cancer hallmark network could be quantified by optimized network scoring functions and the nodes and links in network could be weighted. We are also trying to quantify the working network and transform it into a dynamic system. Our work provided a preliminary method to quantify the concentrations/activities of components and their interaction strengths in working network, and helped to integrate data to elucidate mechanism of intratumor heterogeneity.

In conclusion, we constructed an endogenous molecular network of GC that can be expanded in future and our work suggested that particular PFLs are responsible for producing and maintaining heterogeneous phenotypes of GC cells. Furthermore, we characterized different GC progression routes and corresponding dynamic behaviors of key molecular components. During cancer progression, cancer cells may follow different progression routes. Moreover, it is possible for cells in GC to stay in different cancer attractors or saddle points, indicating that they may manifest gastric and intestinal or even intermediate cancer phenotypes, respectively, a phenomenon that also contributes to intratumor heterogeneity. Our integrated approach combining the cooperative effects of signaling transduction and transcriptional interactions may broaden the mechanism of intratumor heterogeneity.

## SUPPORTING INFORMATION




