# Supplementary material for: Endogenous molecular network reveals two mechanisms of heterogeneity within gastric cancer
Source: Oncotarget. 2015 Apr 24;6(15):13607–27. doi: 10.18632/oncotarget.3633 (PMC4537037; doi:10.18632/oncotarget.3633)
Supplement: Supplementary file 1 [file oncotarget-06-13607-s001.pdf]

TEXT S1: SUPPORTING INFORMATION

DETAIL DESCRIPTION OF ESSENTIAL MODULES AND THEIR MOLECULAR BASIS

The core endogenous network of gastric epithelium is composed of eight core modules. References supporting the relations between the molecular-cellular endogenous agents in the network are listed in Supporting Table S1.

Cell cycle module

Cell cycle is a fundamental biological process of which is conserved in eukaryote. The gastric epithelium continuously renew through the life of animal due to proliferation of multipotent stem cells [1]. Cell cycle is divided into four phases: G_1, S, G_2, M. G_1-S phase was considered in this model, which is composed of cyclin D-Cdk4/6, cyclin E, Rb, E2F, p21 and p27[2]. Cyclin and cyclin-dependent kinase (CDK) constitute the core components of cell cycle process. Cyclin D-CDK4/6 complex initiates phosphorylation of Rb [2], which then dissociates from E2F resulting in activation of target genes transcription, like Cyclin E [3]. After the R point in late G1 phase, the Cyclin E-CDK2 complex to enable the phosphorylation of appropriate substrates required for entry into S phase. The level of Cyclin D was also regulated by transcription factors and extracellular signals, like c-Myc [4], β-catenin [5–7], NF-κB [8, 9], ERK [10], JNK/p38 [11], SHH [12]. On the other hand, the activities of Cyclin -CDK complexes were inhibited by CDK inhibitors, like p21 [13] and p27 [3, 14]. TGF-β elicited a number of downstream responses to antagonize cell proliferation, which can act throughout induce p21[15, 16]. The Myc could deregulate control of cell cycle progression through induce expression of Cyclin D, CDK4 and Cyclin E [4], and repressing expression of p21 [17, 18], p27 [19, 20]. Activation of p53 by DNA damage could induce cell cycle arrest, partially mediated by transcriptional activation of p21 [18, 21] and transcriptional inhibition of Myc [22, 23]. Gastric differentiation factors Sox2 exhibited cell cycle inhibition effect [24], while Shh exerted positive effects on cell cycle by induce expression of Cyclin D [12].

Cell death module

Cell death plays an essential role during the animal development and tissue homeostasis [25]. Apoptosis, an important form of programmed cell death, mediates physiological gastric epithelial cell loss in the normal gastric mucosa and chronic gastritis [26, 27]. Apoptosis is tightly controlled by either extracellular or intracellular signals. The center executor of apoptosis is caspases, of which activation is under dual control by both apoptosis activators and inhibitors such as Bcl-2, Bcl-XL, BAD, BAK-BAX, Bid and XIAP. The relatives of Bcl-2, such as Bad, Bax, Bak, and Bid, strive to pry open channels in the outer membrane of the mitochondria and thereby release cytochrome *c* into the surrounding cytosol [28, 29]. Yet Bcl-2 and Bcl-XL work oppositely to keep channels closed [30]. Once present in the cytosol, cytochrome *c* molecules associate with the Apaf-1 protein and form apoptosome and then proceeds to activate procaspase 9. The active caspase 9 then proceeds to activate procaspase 3, which executes apoptotic process [31–36]. Except the intrinsic apoptotic pathway, apoptosis can also be triggered through activated pro-apoptotic cell surface receptors, such as Fas and TNFR. These receptors act via the FAS-associated death domain (FADD) protein to assemble a death-inducing signaling complex (DISC), and the latter proceeds to activate caspases 8 and 10 [28, 37]. These then converge on the intrinsic apoptotic cascade by activating the caspases 3 [31–35], the latter cleaves and activates Bid [28], which initiates opening of the outer mitochondrial membrane channel, further activating the apoptotic cascade. Studies had revealed that a variety of stresses could cause a rapid increase in p53 levels. After p53 concentrations increase, the p53 bind to the promoters of a large of target genes and induce their transcription. Among the induced proteins are p21 [18, 21], Bax [38, 39], Fas [38, 39], Bcl-2 [38, 39] and IGF1-R [40, 41]. The survival signaling pathways through activation of the PI3kinase (PI3K)-Akt/PKB kinase pathway leads to Mdm2 phosphorylation and to the resulting p53 activation [42, 43]. The XIAPs bind to and inhibit caspases [44, 45]. XIAPs are bound and inhibited by a protein named SMAC/DIABLO, which is released from mitochondria along with cytochrome c during apoptosis [44]. In response to certain physiologic signals, NF-κB activates a large constituency of target genes involved in apoptosis; included among these are p53 [46], XIAP [46, 47], Bcl-2 [48] and $Bcl\text{-}X_L$ [46, 47].

Inflammation

Cells are capable of sensing various deleterious conditions and respond by mounting a variety of stress responses. Normal signals like cytokines can induce inflammatory responses in cells [49]. Inflammation is a part of normal host protective attempt to eradicate injurious agents and to initiate the process of healing. However, inflammation also exerts influence on tumorigenesis [50]. Gastric cancer involves a transition from normal mucosa to gastritis, which eventually leads to adenocarcinoma [51]. *Helicobacter pylori*-induced gastritis is associated with increased risk of developing gastric adenocarcinoma. In this model, we focused on key inflammatory cytokines TNF-α, IL-1, IL-8, IL-10 and IFN-γ, which are associated with gastritis, atrophy response and development of gastric adenocarcinoma [52, 53]. IL-1 signaling activates the

c-Jun N-terminal kinase (JNK) and p38 MAPK pathway [54–56]. JNK phosphorylates proteins that are part of AP-1, in particularly c-Jun and ATF-2. It has been shown that JNK and c-Jun play a major role in IL-1-induced expression of genes encoding IL-8 [57–61], COX-2 [62–64] and IκB [65]. The JNK and p38 MAPK cascades [57–61] and NF-κB [46, 57, 65, 66] contribute to IL-8 gene expression. TNF signaling activates NF-κB through degradating IκB. NF-κB, a key transcriptional factor of inflammatory response and a critical bridge between inflammation and cancer, are also included [67]. Usually, NF-κB is sequestered in the cytoplasm by IκB (inhibitor of NF-κB). However, in response to diverse signals, IκB become phosphorylated and rapid degraded. As a result, NF-κB is liberated, and proceeds migrates into the nucleus and activates the expression of cohort of target genes, such as Bcl-2 [48], XIAP [46, 47], Myc [46], Cyclin D [8, 9] and COX-2 [46, 68, 69].

Angiogenesis

Angiogenesis is the physiological process beneficial for normal tissue growth and regeneration. However, it also represents a malignant transformation of tumor progression [70]. Under conditions of hypoxia, the HIF transcription factor accumulate, which then drives the expression of a number of genes whose products encourage angiogenesis. Prominent among these is VEGF [71]. VEGF/VEGFR could also be activated by IL-8 [72], IL-1 [73], HGF [74], Integrin [75] and PI3K/Akt signaling [76].

Metabolism

During cell growth and division, adjustment of energy metabolism is observed in neoplastic diseases. Cancer cells carry out increased aerobic glycolysis, a phenomenon known as the Warburg effect [77]. Some proteins, such as Myc, HIF, p53, AKT, Ras and β-catenin, are intimately linked to metabolic pathways through transcriptional or post-transcriptional regulation of metabolic enzymes [78].

Cell adhesion

Cell-to-cell interactions and their underlying extracellular matrix (ECM) present critical signals that regulate and maintain gastric epithelial differentiation, Those interaction mediator including E-cadherin and Integrins [79, 80]. In addition, cancer cells that participated in invasion and metastasis developed alterations in their attachment to other cells and to the extracellular matrix (ECM). The alteration best characterized by losing of E-cadherin, a key cell-to-cell adhesion molecule [81]. Those regulators include TGF-β[79] and a set of transcriptional factors like Zeb1/2 [82].

Gastric differentiation

The gastric epithelial associated factors that participate in gastric epithelium development and differentiation have been indentified as growth factors and transcription factors, such as Sox2, sonic hedgehog (Shh), Indian hedgehog (Ihh), gastrin, FGF10, Gata4, Gata6, BMP4, Runx3, Bapx1, Foxa1/2 and HoxA5. Recent data indicate that the most critical reprogramming factor, Cdx1/2, which inhibit expression of differentiated genes in gastric epithelium, is sufficient to direct the reprogramming of gastric epithelium cells into intestinal cells. On the other hand, the direct reprogramming factor of intestinal cells into gastric-liked epithelial cells has been demonstrated in several models, such as Sox2 and Shh. Therefore, the gastric cells state and intestinal cell state should be maintained by a set of master regulatory factors respectively.

Cdx2 is believed to be critical for maintaining intestinal epithelial cell phenotype [83, 84] and there have been several reports of it expression in intestinal metaplasia and intestinal type gastric carcinomas [85, 86]. Gastric expression of Cdx2 alone was sufficient to induce intestinal metaplasia in mice [87–89]. Beside, increased β-catenin expressions were significantly more frequent in intestinal-type gastric cancer compared with diffuse-type gastric carcinomas [90]. On the other hand, since, in adults, Sox2 expression is found in stomach and absent from the intestine [91]. Moreover, Sox2 participates in the development of foregut-derived organs, such as esophagus and stomach [92, 93], and are involved in regulation of stomach specific genes, pepsinogen and Muc5ac [94, 95]. Ectopic Sox2 expression is sufficient to redirect development fate of intestinal epithelium towards a premature gastric phenotype [96]. In addition, the loss of Sox2 expression and aberrant Cdx2 expression occur in intestinal metaplasia.

Growth factors

Various growth factors and hormones are expressed by mesenchymal cells but act on the epithelial compartment: for example, hepatocyte growth factor (HGF) is known to influence proliferation and differentiation of intestinal epithelial cells. The presence of EGF, HGF, IGF and KGF receptors in all glandular compartments clearly suggests the potential role of these growth factors either in the development or in the maintenance of specific gastric epithelial functions [3]. When the gastric mucosa is injured by inflammation or other insults, expression of growth factors and their receptors, such as EGF, IGF, EGFR family, and VEGFR

subtypes, are upregulated in epithelial or mesenchymal cells, and subsequently mucosal repair is promoted [97]. Ras can be activated by a number of signaling cascades radiating from growth factor receptors, such as EGF[98], HGF[99], VEGF [100] and Integrins [42, 43, 101, 102]. Ras activates Erk1 and Erk2 (MAPKs) via the Raf kinase [103]. The latter can then phosphorylate kinases in the cytoplasm that regulate transcription as well as transcription factors (Ets, Elk-1, SAP-1), which can then proceed to stimulate the expression of growth-regulating genes, such as Cyclin D [10], Myc [104], p21 [105] and HGF [106]. Ras protein is also capable to phosphorylate and activate phosphatidylinositol 3-kinase (PI3K) and AKT/PKB pathway. Once activated, Akt/PKB proceeds to phosphorylate and activate/inhibite a series of protein substrates, such as Bad [107], caspase-9 [108, 109], p21 [110] and GSK3β [111]. The PI3K-AKT pathway mainly aid cell survival and stimulate cell proliferation. The activation of PI3K and Akt/PKB is negatively control by PTEN [112].

DIFFERENTIAL EQUATIONS TO QUANTIFY THE WORKING ENDOGENOUS NETWORK

We translated the endogenous molecular network for gastric epithelium Figure 1 into mathematical format by employing sigmoidal functions. The quantitative descriptions are a set of coupled ordinary differential functions as follow. The meanings of parameters are in Methods. The computational codes are available upon request.

Supporting Table S1: Supporting information

The supporting information includes 3 parts: supporting methods, supporting tables and supporting figures.

$x(1) = Rb$	Unphosphorylated retinoblastoma protein
$x(2) = Cyclin\,D/CDK4, 6$	Cyclin D/Cdk4,6 complex
$x(3) = Cyclin\,E/CDK2$	Cyclin E/Cdk2 complex
$x(4) = c\text{-}Myc$	Myc/Max dimer
$x(5) = E2F$	E2F1,2 and E2F3a
$x(6) = p21$	
$x(7) = p27$	
$x(8) = p53$	
$x(9) = Caspase\,3, 7$	Caspase 3 and Caspase 7
$x(10) = Cytochrome\,c$	Mitochondrial proteins, including Cytochrome c, Apaf-1, Caspase 9, SMAC/ DIABLO, Endo G, AIF and CAD.
$x(11) = Caspase\,8, 10$	Caspase 8 and Caspase 10
$x(12) = XIAP$	
$x(13) = Bcl\text{-}2$	
$x(14) = Bcl\text{-}xL$	
$x(15) = Bid$	
$x(16) = BAD$	
$x(17) = Bax$	
$x(18) = Ras$	Ras super family, refers to HRAS, KRAS and NRAS.
$x(19) = Akt$	
$x(20) = PTEN$	
$x(21) = ERK$	The extracellular-signal-regulated kinase (ERK) pathway
$x(22) = JNK/p38$	The JUN N-terminal kinase (JNK) pathway and the p38 pathway
$x(23) = MKP$	Dual-specificity MAPK phosphatases; also known as dual specificity protein phosphatases (DUSPs), mainly refers to DUSP1 here.

$x(24) = VEGF/VEGFR$	VEGF and VEGF receptors
$x(25) = EGF/EGFR$	EGF family protein and ErbB family, mainly refers to EGF, HB-EGF, ErbB1/2.
$x(26) = IGF/EGFR$	Insulin-like growth factor (IGF) and IGF receptors
$x(27) = HGF/Met$	Hepatocyte growth factor(HGF) and c-Met receptor
$x(28) = Integrin/FAK$	Integrin/FAK signaling pathway
$x(29) = E\text{-}cadherin$	
$x(30) = Zeb1/2$	The ZEB family, including ZEB1 (also known as TCF8 and δEF1) and ZEB2 (also known as ZFXH1B and SMAD interacting protein 1 (SIP1))
$x(31) = \beta\ \text{-}catenin$	Wnt/β-catenin signaling pathway
$x(32) = HIF$	HIF family
$x(33) = NF\text{-}kB$	
$x(34) = ikB$	
$x(35) = TNF\text{-}\alpha$	
$x(36) = IL\text{-}10$	Interleukin-10 and its receptor
$x(37) = IL\text{-}1$	Interleukin-1 family, refers to IL-1α, IL-1β, and IL-18 and their receptors.
$x(38) = IL\text{-}8$	Interleukin-8 and receptors CXCR1 and CXCR2.
$x(39) = Fas$	Fas ligand or CD95L, and its receptors FasR and DcR3.
$x(40) = COX\text{-}2$	
$x(41) = TGF\text{-}\beta$	TGF-β family
$x(42) = IFN\text{-}\gamma$	
$x(43) = STAT3$	Signal transducer and activator of transcription 3
$x(44) = GSK\text{-}3\beta$	
$x(45) = Gastrin/CC2R$	Gastrin and it receptor
$x(46) = Sox2$	
$x(47) = Cdx2$	
$x(48) = Shh$	Sonic hedgehog signaling

$$\frac{dx(1)}{dt} = \frac{1}{1 + a(1) * x(2)^{n(1)} + a(2) * x(3)^{n(2)} + a(3) * x(9)^{n(3)}} - x(1);$$

$$\frac{dx(2)}{dt} = \frac{a(4) * x(4)^{n(4)} + a(5) * x(31)^{n(5)} + a(6) * x(33)^{n(6)} + a(7) * x(21)^{n(7)} + a(8) * x(22)^{n(8)} + a(9) * x(48)^{n(9)}}{1 + a(4) * x(4)^{n(4)} + a(5) * x(31)^{n(5)} + a(6) * x(33)^{n(6)} + a(7) * x(21)^{n(7)} + a(8) * x(22)^{n(8)} + a(9) * x(48)^{n(9)}}$$
$$* \frac{1}{1 + a(10) * x(6)^{n(10)} + a(11) * x(44)^{n(11)} + a(12) * x(46)^{n(12)}} - x(2);$$

$$\frac{dx(3)}{dt} = \frac{a(13) * x(4)^{n(13)} + a(14) * x(5)^{n(14)}}{1 + a(13) * x(4)^{n(13)} + a(14) * x(5)^{n(14)}} * \frac{1}{1 + a(15) * x(6)^{n(15)} + a(16) * x(7)^{n(16)}} - x(3);$$

$$\frac{dx(4)}{dt} = \frac{a(17) * x(5)^{n(17)} + a(18) * x(19)^{n(18)} + a(19) * x(21)^{n(19)} + a(20) * x(22)^{n(20)} + a(21) * x(31)^{n(21)} + a(22) * x(33)^{n(22)} + a(23) * x(32)^{n(23)} + a(24) * x(48)^{n(24)}}{1 + a(17) * x(5)^{n(17)} + a(18) * x(19)^{n(18)} + a(19) * x(21)^{n(19)} + a(20) * x(22)^{n(20)} + a(21) * x(31)^{n(21)} + a(22) * x(33)^{n(22)} + a(23) * x(32)^{n(23)} + a(24) * x(48)^{n(24)}}$$
$$* \frac{1}{1 + a(25) * x(8)^{n(25)} + a(26) * x(41)^{n(26)} + a(27) * x(42)^{n(27)} + a(28) * x(6)^{n(28)}} - x(4);$$

$$\frac{dx(5)}{dt} = \frac{a(29) * x(5)^{n(29)} + a(30) * x(4)^{n(30)}}{1 + a(29) * x(5)^{n(29)} + a(30) * x(4)^{n(30)}} * \frac{1}{1 + a(31) * x(1)^{n(31)} + a(32) * x(6)^{n(32)}} - x(5);$$

$$\frac{dx(6)}{dt} = \frac{a(33) * x(8)^{n(33)} + a(34) * x(41)^{n(34)} + a(35) * x(5)^{n(35)} + a(36) * x(42)^{n(36)} + a(37) * x(32)^{n(37)} + a(38) * x(21)^{n(38)} + a(39) * x(47)^{n(39)}}{1 + a(33) * x(8)^{n(33)} + a(34) * x(41)^{n(34)} + a(35) * x(5)^{n(35)} + a(36) * x(42)^{n(36)} + a(37) * x(32)^{n(37)} + a(38) * x(21)^{n(38)} + a(39) * x(47)^{n(39)}}$$
$$* \frac{1}{1 + a(40) * x(4)^{n(40)} + a(41) * x(19)^{n(41)}} - x(6);$$

$$\frac{dx(7)}{dt} = \frac{a(42) * x(29)^{n(42)} + a(43) * x(41)^{n(43)} + a(44) * x(42)^{n(44)} + a(45) * x(20)^{n(45)} + a(46) * x(46)^{n(46)}}{1 + a(42) * x(29)^{n(42)} + a(43) * x(41)^{n(43)} + a(44) * x(42)^{n(44)} + a(45) * x(20)^{n(45)} + a(46) * x(46)^{n(46)}}$$
$$* \frac{1}{1 + a(47) * x(4)^{n(47)} + a(48) * x(19)^{n(48)} + a(49) * x(3)^{n(49)} + a(50) * x(21)^{n(50)}} - x(7);$$

$$\frac{dx(8)}{dt} = \frac{a(51) * x(5)^{n(51)} + a(52) * x(4)^{n(52)} + a(53) * x(33)^{n(53)} + a(54) * x(10)^{n(54)}}{1 + a(51) * x(5)^{n(51)} + a(52) * x(4)^{n(52)} + a(53) * x(33)^{n(53)} + a(54) * x(10)^{n(54)}}$$
$$* \frac{1}{1 + a(55) * x(19)^{n(55)}} - x(8);$$

$$\frac{dx(9)}{dt} = \frac{a(56) * x(10)^{n(56)} + a(57) * x(11)^{n(57)}}{1 + a(56) * x(10)^{n(56)} + a(57) * x(11)^{n(57)}} * \frac{1}{1 + a(58) * x(12)^{n(58)} + a(59) * x(6)^{n(59)}} - x(9);$$

$$\frac{dx(10)}{dt} = \frac{a(60) * x(16)^{n(60)} + a(61) * x(17)^{n(61)} + a(62) * x(15)^{n(62)} + a(63) * x(5)^{n(63)}}{1 + a(60) * x(16)^{n(60)} + a(61) * x(17)^{n(61)} + a(62) * x(15)^{n(62)} + a(63) * x(5)^{n(63)}}$$
$$* \frac{1}{1 + a(64) * x(19)^{n(64)} + a(65) * x(12)^{n(65)}} - x(10);$$

$$\frac{dx(11)}{dt} = \frac{a(66) * x(39)^{n(66)} + a(67) * x(35)^{n(67)} + a(68) * x(37)^{n(68)}}{1 + a(66) * x(39)^{n(66)} + a(67) * x(35)^{n(67)} + a(68) * x(37)^{n(68)}} - x(11);$$

$$\frac{dx(12)}{dt} = \frac{a(69) * x(19)^{n(69)} + a(70) * x(33)^{n(70)}}{1 + a(69) * x(19)^{n(69)} + a(70) * x(33)^{n(70)}} * \frac{1}{1 + a(71) * x(10)^{n(71)}} - x(12);$$

$$\frac{dx(13)}{dt} = \frac{a(72) * x(24)^{n(72)} + a(73) * x(33)^{n(73)} + a(74) * x(21)^{n(74)} + a(75) * x(48)^{n(75)}}{1 + a(72) * x(24)^{n(72)} + a(73) * x(33)^{n(73)} + a(74) * x(21)^{n(74)} + a(75) * x(48)^{n(75)}} - x(13);$$

$$\frac{dx(14)}{dt} = \frac{a(80) * x(25)^{n(80)} + a(81) * x(26)^{n(81)} + a(82) * x(33)^{n(82)}}{1 + a(80) * x(25)^{n(80)} + a(81) * x(26)^{n(81)} + a(82) * x(33)^{n(82)}} - x(14);$$

$$\frac{dx(15)}{dt} = \frac{a(85) * x(11)^{n(85)}}{1 + a(85) * x(11)^{n(85)}} - x(15);$$

$$\frac{dx(16)}{dt} = \frac{a(86) * x(8)^{n(86)}}{1 + a(86) * x(8)^{n(86)}} * \frac{1}{1 + a(87) * x(19)^{n(87)} + a(88) * x(21)^{n(88)}} - x(16);$$

$$\frac{dx(17)}{dt} = \frac{a(89) * x(4)^{n(89)} + a(90) * x(8)^{n(90)}}{1 + a(89) * x(4)^{n(89)} + a(90) * x(8)^{n(90)}} * \frac{1}{1 + a(91) * x(13)^{n(91)} + a(92) * x(14)^{n(92)}} - x(17);$$

$$\frac{dx(18)}{dt} = \frac{a(93) * x(27)^{n(93)} + a(94) * x(25)^{n(94)} + a(95) * x(24)^{n(95)} + a(96) * x(28)^{n(96)}}{1 + a(93) * x(27)^{n(93)} + a(94) * x(25)^{n(94)} + a(95) * x(24)^{n(95)} + a(96) * x(28)^{n(96)}} - x(18);$$

$$\frac{dx(19)}{dt} = \frac{a(97) * x(38)^{n(97)} + a(98) * x(18)^{n(98)} + a(99) * x(26)^{n(99)} + a(100) * x(29)^{n(100)} + a(101) * x(28)^{n(101)} + a(102) * x(27)^{n(102)} + a(103) * x(24)^{n(103)} + a(104) * x(25)}{1 + a(97) * x(38)^{n(97)} + a(98) * x(18)^{n(98)} + a(99) * x(26)^{n(99)} + a(100) * x(29)^{n(100)} + a(101) * x(28)^{n(101)} + a(102) * x(27)^{n(102)} + a(103) * x(24)^{n(103)} + a(104) * x(2}$$

$$* \frac{1}{1 + a(106) * x(20)^{n(106)}} - x(19);$$

$$\frac{dx(20)}{dt} = \frac{1}{1 + a(107) * x(19)^{n(107)} + a(108) * x(33)^{n(108)} + a(109) * x(31)^{n(109)}} - x(20);$$

$$\frac{dx(21)}{dt} = \frac{a(110) * x(18)^{n(110)} + a(111) * x(28)^{n(111)} + a(112) * x(43)^{n(112)} + a(113) * x(45)^{n(113)}}{1 + a(110) * x(18)^{n(110)} + a(111) * x(28)^{n(111)} + a(112) * x(43)^{n(112)} + a(113) * x(45)^{n(113)}}$$

$$* \frac{1}{1 + a(114) * x(20)^{n(114)} + a(115) * x(23)^{n(115)}} - x(21);$$

$$\frac{dx(22)}{dt} = \frac{a(116) * x(41)^{n(116)} + a(117) * x(35)^{n(117)} + a(118) * x(37)^{n(118)} + a(119) * x(38)^{n(119)} + a(120) * x(24)^{n(120)} + a(121) * x(25)^{n(121)} + a(122) * x(45)^{n(122)}}{1 + a(116) * x(41)^{n(116)} + a(117) * x(35)^{n(117)} + a(118) * x(37)^{n(118)} + a(119) * x(38)^{n(119)} + a(120) * x(24)^{n(120)} + a(121) * x(25)^{n(121)} + a(122) * x(45)^{n(122)}}$$

$$* \frac{1}{1 + a(123) * x(20)^{n(123)} + a(124) * x(23)^{n(124)}} - x(22);$$

$$\frac{dx(23)}{dt} = \frac{a(125) * x(21)^{n(125)} + a(126) * x(22)^{n(126)}}{1 + a(125) * x(21)^{n(125)} + a(126) * x(22)^{n(126)}} - x(23);$$

$$\frac{dx(24)}{dt} = \frac{a(127) * x(38)^{n(127)} + a(128) * x(32)^{n(128)} + a(129) * x(19)^{n(129)} + a(130) * x(27)^{n(130)} + a(131) * x(40)^{n(131)} + a(132) * x(37)^{n(132)} + a(133) * x(28)^{n(133)} + a(134) *}{1 + a(127) * x(38)^{n(127)} + a(128) * x(32)^{n(128)} + a(12) * x(19)^{n(129)} + a(130) * x(27)^{n(130)} + a(131) * x(40)^{n(131)} + a(132) * x(37)^{n(132)} + a(133) * x(28)^{n(133)} + a(134)}$$

$$\frac{dx(25)}{dt} = \frac{a(135) * x(38)^{n(135)} + a(136) * x(28)^{n(136)}}{1 + a(135) * x(38)^{n(135)} + a(136) * x(28)^{n(136)}} - x(25);$$

$$\frac{dx(26)}{dt} = \frac{a(137) * x(32)^{n(137)}}{1 + a(137) * x(32)^{n(137)}} - x(26);$$

$$\frac{dx(27)}{dt} = \frac{a(139) * x(21)^{n(139)} + a(140) * x(32)^{n(140)} + a(141) * x(31)^{n(141)}}{1 + a(139) * x(21)^{n(139)} + a(140) * x(32)^{n(140)} + a(141) * x(31)^{n(141)}} - x(27);$$

$$\frac{dx(28)}{dt} = \frac{a(142) * x(25)^{n(142)} + a(143) * x(27)^{n(143)}}{1 + a(142) * x(25)^{n(142)} + a(143) * x(27)^{n(143)}} * \frac{1}{1 + a(144) * x(20)^{n(144)}} - x(28);$$

$$\frac{dx(29)}{dt} = \frac{1}{1 + a(145) * x(28)^{n}(145) + a(146) * x(37)^{n}(146) + a(147) * x(27)^{n}(147) + a(148) * x(30)^{n}(148) + a(149) * x(9)^{n}(149)} - x(29);$$

$$\frac{dx(30)}{dt} = \frac{a(150) * x(41)^{n(150)} + a(151) * x(33)^{n(151)}}{1 + a(150) * x(41)^{n(150)} + a(151) * x(33)^{n(151)}} * \frac{1}{1 + a(152) * x(8)^{n(152)}} - x(30);$$

$$\frac{dx(31)}{dt} = \frac{a(153) * x(45)^{n(153)} + a(154) * x(27)^{n(154)} + a(155) * x(25)^{n(155)} + a(156) * x(26)^{n(156)}}{1 + a(153) * x(45)^{n(153)} + a(154) * x(27)^{n(154)} + a(155) * x(25)^{n(155)} + a(156) * x(26)^{n(156)}}$$

$$* \frac{1}{1 + a(157) * x(29)^{n(157)} + a(158) * x(44)^{n(158)} + a(159) * x(46)^{n(159)}} - x(31);$$

$$\frac{dx(32)}{dt} = \frac{a(160) * x(19)^{n(160)} + a(161) * x(33)^{n(161)} + a(162) * x(4)^{n(162)}}{1 + a(160) * x(19)^{n(160)} + a(161) * x(33)^{n(161)} + a(162) * x(4)^{n(162)}} * \frac{1}{1 + a(163) * x(8)^{n(163)}} - x(32);$$

$$\frac{dx(33)}{dt} = \frac{a(164) * x(22)^{n(164)}}{1 + a(164) * x(22)^{n(164)}} * \frac{1}{1 + a(165) * x(34)^{n(165)} + a(166) * x(8)^{n(166)}} - x(33);$$

$$\frac{dx(34)}{dt} = \frac{a(167) * x(33)^{n(167)} + a(168) * x(21)^{n(168)} + a(169) * x(22)^{n(169)}}{1 + a(167) * x(33)^{n(167)} + a(168) * x(21)^{n(168)} + a(169) * x(22)^{n(169)}}$$

$$* \frac{1}{1 + a(170) * x(37)^{n(170)} + a(171) * x(19)^{n(171)} + a(172) * x(35)^{n(172)} + a(173) * x(39)^{n(173)} + a(174) * x(41)^{n(174)}} -$$

$$\frac{dx(35)}{dt} = \frac{a(175) * x(33)^{n(175)} + a(176) * x(21)^{n(176)} + a(177) * x(22)^{n(177)}}{1 + a(175) * x(33)^{n(175)} + a(176) * x(21)^{n(176)} + a(177) * x(22)^{n(177)}} * \frac{1}{1 + a(178) * x(36)^{n(178)}} - x(35);$$

$$\frac{dx(36)}{dt} = \frac{a(179) * x(35)^{n(179)} + a(180) * x(39)^{n(180)}}{1 + a(179) * x(35)^{n(179)} + a(180) * x(39)^{n(180)}} * \frac{1}{1 + a(181) * x(42)^{n(181)}} - x(36);$$

$$\frac{dx(37)}{dt} = \frac{a(182) * x(33)^{n(182)} + a(183) * x(22)^{n(183)}}{1 + a(182) * x(33)^{n(182)} + a(183) * x(22)^{n(183)}} * \frac{1}{1 + a(184) * x(37)^{n(184)}} - x(37);$$

$$\frac{dx(38)}{dt} = \frac{a(185) * x(33)^{n(185)} + a(186) * x(22)^{n(186)}}{1 + a(185) * x(33)^{n(185)} + a(186) * x(22)^{n(186)}} - x(38);$$

$$\frac{dx(39)}{dt} = \frac{a(187) * x(8)^{n(187)} + a(188) * x(33)^{n(188)} + a(189) * x(42)^{n(189)}}{1 + a(187) * x(8)^{n(187)} + a(188) * x(33)^{n(188)} + a(189) * x(42)^{n(189)}} * \frac{1}{1 + a(190) * x(18)^{n(189)}} - x(39);$$

$$\frac{dx(40)}{dt} = \frac{a(191) * x(33)^{n(191)} + a(192) * x(22)^{n(192)}}{1 + a(191) * x(33)^{n(191)} + a(192) * x(22)^{n(192)}} - x(40);$$

$$\frac{dx(41)}{dt} = \frac{a(193) * x(28)^{n(193)} + a(194) * x(32)^{n(194)}}{1 + a(193) * x(28)^{n(193)} + a(194) * x(32)^{n(194)}} * \frac{1}{1 + a(195) * x(33)^{n(195)}} - x(41);$$

$$\frac{dx(42)}{dt} = \frac{a(196) * x(37)^{n(196)}}{1 + a(196) * x(37)^{n(196)}} * \frac{1}{1 + a(197) * x(36)^{n(197)}} - x(42);$$

$$\frac{dx(43)}{dt} = \frac{a(198) * x(36)^{n(198)} + a(199) * x(45)^{n(199)} + a(200) * x(25)^{n(200)} + a(201) * x(27)^{n(201)}}{1 + a(198) * x(36)^{n(198)} + a(199) * x(45)^{n(199)} + a(200) * x(25)^{n(200)} + a(201) * x(27)^{n(201)}} * \frac{1}{1 + a(202) * x(41)^{n(2[illegible]}}$$

$$\frac{dx(44)}{dt} = \frac{1}{1 + a(203) * x(19)^{n(203)} + a(204) * x(33)^{n(204)}} - x(44);$$

$$\frac{dx(45)}{dt} = \frac{a(205) * x(31)^{n(205)}}{1 + a(205) * x(31)^{n(205)}} - x(45);$$

$$\frac{dx(46)}{dt} = \frac{a(206) * x(46)^{n(206)} + a(207) * x(48)^{n(207)} + a(208) * x(43)^{n(208)}}{1 + a(206) * x(46)^{n(206)} + a(207) * x(48)^{n(207)} + a(208) * x(43)^{n(208)}} * \frac{1}{1 + a(209) * x(42)^{n(209)}} - x(46);$$

$$\frac{dx(47)}{dt} = \frac{a(210) * x(31)^{n(210)} + a(211) * x(47)^{n(211)}}{1 + a(210) * x(31)^{n(210)} + a(211) * x(47)^{n(211)}} * \frac{1}{1 + a(212) * x(46)^{n(212)}} - x(47);$$

$$\frac{dx(48)}{dt} = \frac{a(213) * x(46)^{n(213)}}{1 + a(213) * x(46)^{n(213)}} * \frac{1}{1 + a(214) * x(47)^{n(214)} + a(215) * x(44)^{n(215)}} - x(48)$$

REFERENCES

1. Qiao XT, Ziel JW, McKimpson W, Madison BB, Todisco A, Merchant JL, Samuelson LC, Gumucio DL. Prospective identification of a multilineage progenitor in murine stomach epithelium. Gastroenterology. 2007; 133:1989–1998.
2. Weinberg RA. The retinoblastoma protein and cell cycle control. Cell. 1995; 81:323–330.
3. Blais A, Dynlacht BD. Hitting their targets: an emerging picture of E2F and cell cycle control. Curr Opin Genet Dev. 2004; 14:527–532.
4. Obaya AJ, Mateyak MK, Sedivy JM. Mysterious liaisons: the relationship between c-Myc and the cell cycle. Oncogene. 1999; 18:2934–2941.
5. Hatsell S, Rowlands T, Hiremath M, Cowin P. Beta-catenin and Tcfs in mammary development and cancer. J Mammary Gland Biol Neoplasia. 2003; 8:145–158.
6. Tetsu O, McCormick F. Beta-catenin regulates expression of cyclin D1 in colon carcinoma cells. Nature. 1999; 398:422–426.
7. Shtutman M, Zhurinsky J, Simcha I, Albanese C, D'Amico M, Pestell R, Ben-Ze'ev A. The cyclin D1 gene is a target of the beta-catenin/LEF-1 pathway. P Natl Acad Sci USA. 1999; 96:5522–5527.
8. Hinz M, Krappmann D, Eichten A, Heder A, Scheidereit C, Strauss M. NF-kappaB function in growth control: regulation of cyclin D1 expression and G0/G1-to-S-phase transition. Molecular and Cellular Biology. 1999; 19:2690–2698.
9. Guttridge DC, Albanese C, Reuther JY, Pestell RG, Baldwin AS Jr. NF-kappaB controls cell growth and differentiation through transcriptional regulation of cyclin D1. Molecular and Cellular Biology. 1999; 19:5785–5799.
10. Cheng M, Sexl V, Sherr CJ, Roussel MF. Assembly of cyclin D-dependent kinase and titration of p27Kip1 regulated by mitogen-activated protein kinase kinase (MEK1). Proc Natl Acad Sci U S A. 1998; 95:1091–1096.
11. Bakiri L, Lallemand D, Bossy-Wetzel E, Yaniv M. Cell cycle-dependent variations in c-Jun and JunB phosphorylation: a role in the control of cyclin D1 expression. EMBO J. 2000; 19:2056–2068.
12. Merchant JL. Hedgehog signalling in gut development, physiology and cancer. J Physiol. 2012; 590:421–432.
13. Sherr CJ, Roberts JM. CDK inhibitors: positive and negative regulators of G1-phase progression. Genes Dev. 1999; 13:1501–1512.
14. Sherr CJ. Cancer cell cycles. Science. 1996; 274:1672–1677.
15. Datto MB, Li Y, Panus JF, Howe DJ, Xiong Y, Wang XF. Transforming growth factor beta induces the cyclin-dependent kinase inhibitor p21 through a p53-independent mechanism. Proc Natl Acad Sci U S A. 1995; 92:5545–5549.
16. Datto MB, Yu Y, Wang XF. Functional analysis of the transforming growth factor beta responsive elements in the WAF1/Cip1/p21 promoter. Journal of Biological Chemistry. 1995; 270:28623–28628.
17. Seoane J, Le HV, Massague J. Myc suppression of the p21(Cip1) Cdk inhibitor influences the outcome of the p53 response to DNA damage. Nature. 2002; 419:729–734.
18. Gartel AL, Radhakrishnan SK. Lost in transcription: p21 repression, mechanisms, and consequences. Cancer Research. 2005; 65:3980–3985.
19. Slingerland J, Pagano M. Regulation of the cdk inhibitor p27 and its deregulation in cancer. J Cell Physiol. 2000; 183:10–17.
20. Dang CV. c-Myc target genes involved in cell growth, apoptosis, and metabolism. Molecular and Cellular Biology. 1999; 19:1–11.
21. el-Deiry WS, Tokino T, Velculescu VE, Levy DB, Parsons R, Trent JM, Lin D, Mercer WE, Kinzler KW, Vogelstein B. WAF1, a potential mediator of p53 tumor suppression. Cell. 1993; 75:817–825.
22. Sachdeva M, Zhu S, Wu F, Wu H, Walia V, Kumar S, Elble R, Watabe K, Mo YY. p53 represses c-Myc through induction of the tumor suppressor miR-145. Proc Natl Acad Sci U S A. 2009; 106:3207–3212.
23. Ho JS, Ma W, Mao DY, Benchimol S. p53-Dependent transcriptional repression of c-myc is required for G1 cell cycle arrest. Molecular and Cellular Biology. 2005; 25:7423–7431.
24. Otsubo T, Akiyama Y, Yanagihara K, Yuasa Y. SOX2 is frequently downregulated in gastric cancers and inhibits cell growth through cell-cycle arrest and apoptosis. Br J Cancer. 2008; 98:824–831.
25. Fuchs Y, Steller H. Programmed cell death in animal development and disease. Cell. 2011; 147:742–758.
26. Oshima H, Matsunaga A, Fujimura T, Tsukamoto T, Taketo MM, Oshima M. Carcinogenesis in mouse stomach by simultaneous activation of the Wnt signaling and prostaglandin E2 pathway. Gastroenterology. 2006; 131:1086–1095.
27. Xia HH, Talley NJ. Apoptosis in gastric epithelium induced by Helicobacter pylori infection: implications in gastric carcinogenesis. Am J Gastroenterol. 2001; 96:16–26.
28. Igney FH, Krammer PH. Death and anti-death: tumour resistance to apoptosis. Nat Rev Cancer. 2002; 2:277–288.
29. Arnoult D, Gaume B, Karbowski M, Sharpe JC, Cecconi F, Youle RJ. Mitochondrial release of AIF and EndoG requires caspase activation downstream of Bax/Bak-mediated permeabilization. EMBO J. 2003; 22:4385–4399.
30. Chipuk JE, Bhat M, Hsing AY, Ma J, Danielpour D. Bcl-xL blocks transforming growth factor-beta 1-induced apoptosis by inhibiting cytochrome c release and not by directly antagonizing Apaf-1-dependent caspase activation in

prostate epithelial cells. Journal of Biological Chemistry. 2001; 276:26614–26621.

31. Budihardjo I, Oliver H, Lutter M, Luo X, Wang X. Biochemical pathways of caspase activation during apoptosis. Annu Rev Cell Dev Biol. 1999; 15:269–290.

32. Chang HY, Yang X. Proteases for cell suicide: functions and regulation of caspases. Microbiol Mol Biol Rev. 2000; 64:821–846.

33. Zou H, Yang R, Hao J, Wang J, Sun C, Fesik SW, Wu JC, Tomaselli KJ, Armstrong RC. Regulation of the Apaf-1/caspase-9 apoptosome by caspase-3 and XIAP. Journal of Biological Chemistry. 2003; 278:8091–8098.

34. Liu Z, Li H, Derouet M, Filmus J, LaCasse EC, Korneluk RG, Kerbel RS, Rosen KV. ras Oncogene triggers up-regulation of cIAP2 and XIAP in intestinal epithelial cells: epidermal growth factor receptor-dependent and -independent mechanisms of ras-induced transformation. Journal of Biological Chemistry. 2005; 280:37383–37392.

35. Adrain C, Martin SJ. The mitochondrial apoptosome: a killer unleashed by the cytochrome seas. Trends Biochem Sci. 2001; 26:390–397.

36. Bratton SB, Walker G, Srinivasula SM, Sun XM, Butterworth M, Alnemri ES, Cohen GM. Recruitment, activation and retention of caspases-9 and -3 by Apaf-1 apoptosome and associated XIAP complexes. EMBO J. 2001; 20:998–1009.

37. Gaur U, Aggarwal BB. Regulation of proliferation, survival and apoptosis by members of the TNF superfamily. Biochem Pharmacol. 2003; 66:1403–1408.

38. Vogelstein B, Lane D, Levine AJ. Surfing the p53 network. Nature. 2000; 408:307–310.

39. Levine AJ. p53, the cellular gatekeeper for growth and division. Cell. 1997; 88:323–331.

40. Furstenberger G, Senn HJ. Insulin-like growth factors and cancer. Lancet Oncol. 2002; 3:298–302.

41. Ohlsson C, Kley N, Werner H, LeRoith D. p53 regulates insulin-like growth factor-I (IGF-I) receptor expression and IGF-I-induced tyrosine phosphorylation in an osteosarcoma cell line: interaction between p53 and Sp1. Endocrinology. 1998; 139:1101–1107.

42. Khwaja A, RodriguezViciana P, Wennstrom S, Warne PH, Downward J. Matrix adhesion and Ras transformation both activate a phosphoinositide 3-OH kinase and protein kinase B/Akt cellular survival pathway. Embo Journal. 1997; 16:2783–2793.

43. Frisch SM, Francis H. Disruption of epithelial cell-matrix interactions induces apoptosis. J Cell Biol. 1994; 124:619–626.

44. Deveraux QL, Reed JC. IAP family proteins–suppressors of apoptosis. Genes Dev. 1999; 13:239–252.

45. Salvesen GS, Duckett CS. IAP proteins: blocking the road to death’s door. Nat Rev Mol Cell Biol. 2002; 3:401–410.

46. Pahl HL. Activators and target genes of Rel/NF-kappaB transcription factors. Oncogene. 1999; 18:6853–6866.

47. Karin M, Lin A. NF-kappaB at the crossroads of life and death. Nature immunology. 2002; 3:221–227.

48. Catz SD, Johnson JL. Transcriptional regulation of bcl-2 by nuclear factor kappa B and its significance in prostate cancer. Oncogene. 2001; 20:7342–7351.

49. Berridge MJ. (2012). Cell Signalling Biology: Portland Press.

50. Grivennikov SI, Greten FR, Karin M. Immunity, inflammation, and cancer. Cell. 2010; 140:883–899.

51. Fox JG, Wang TC. Inflammation, atrophy, and gastric cancer. J Clin Invest. 2007; 117:60–69.

52. Sakamoto N, Saiga H, Yasugi S. Analysis of temporal expression pattern and cis-regulatory sequences of chicken pepsinogen A and C. Biochemical and biophysical research communications. 1998; 250:420–424.

53. Sasaki H, Hogan BL. Enhancer analysis of the mouse HNF-3 beta gene: regulatory elements for node/notochord and floor plate are independent and consist of multiple sub-elements. Genes to cells: devoted to molecular & cellular mechanisms. 1996; 1:59–72.

54. Finch A, Holland P, Cooper J, Saklatvala J, Kracht M. Selective activation of JNK/SAPK by interleukin-1 in rabbit liver is mediated by MKK7. Febs Lett. 1997; 418(1-2):144–148.

55. Holtmann H, Enninga J, Kalble S, Thiefes A, Dorrie A, Broemer M, Winzen R, Wilhelm A, Ninomiya-Tsuji J, Matsumoto K, Resch K, Kracht M. The MAPK kinase kinase TAK1 plays a central role in coupling the interleukin-1 receptor to both transcriptional and RNA-targeted mechanisms of gene regulation. Journal of Biological Chemistry. 2001; 276:3508–3516.

56. Hammaker DR, Boyle DL, Chabaud-Riou M, Firestein GS. Regulation of c-Jun N-terminal kinase by MEKK-2 and mitogen-activated protein kinase kinase kinases in rheumatoid arthritis. J Immunol. 2004; 172:1612–1618.

57. Holtmann H, Winzen R, Holland P, Eickemeier S, Hoffmann E, Wallach D, Malinin NL, Cooper JA, Resch K, Kracht M. Induction of interleukin-8 synthesis integrates effects on transcription and mRNA degradation from at least three different cytokine- or stress-activated signal transduction pathways. Molecular and Cellular Biology. 1999; 19:6742–6753.

58. Winzen R, Thakur BK, Dittrich-Breiholz O, Shah M, Redich N, Dhamija S, Kracht M, Holtmann H. Functional analysis of KSRP interaction with the AU-rich element of interleukin-8 and identification of inflammatory mRNA targets. Molecular and Cellular Biology. 2007; 27:8388–8400.

59. Hoffmann E, Thiefes A, Buhrow D, Dittrich-Breiholz O, Schneider H, Resch K, Kracht M. MEK1-dependent delayed expression of Fos-related antigen-1 counteracts c-Fos and p65 NF-kappa B-mediated interleukin-8 transcription in response to cytokines or growth factors. Journal of Biological Chemistry. 2005; 280:9706–9718.
60. Krause A, Holtmann H, Eickemeier S, Winzen R, Szamel M, Resch K, Saklatvala J, Kracht M. Stress-activated protein kinase Jun N-terminal kinase is required for interleukin (IL)-1-induced IL-6 and IL-8 gene expression in the human epidermal carcinoma cell line KB. Journal of Biological Chemistry. 1998; 273:23681–23689.
61. Hoffmann E, Ashouri J, Wolter S, Doerrie A, Dittrich-Breiholz O, Schneider H, Wagner EF, Troppmair J, Mackman N, Kracht M. Transcriptional regulation of EGR-1 by the interleukin-1-JNK-MKK7-c-Jun pathway. Journal of Biological Chemistry. 2008; 283:12120–12128.
62. Chang YJ, Wu MS, Lin JT, Chen CC. Helicobacter pylori-induced invasion and angiogenesis of gastric cells is mediated by cyclooxygenase-2 induction through TLR2/TLR9 and promoter regulation. Journal of Immunology. 2005; 175:8242–8252.
63. Wu G, Luo J, Rana JS, Laham R, Sellke FW, Li J. Involvement of COX-2 in VEGF-induced angiogenesis via P38 and JNK pathways in vascular endothelial cells. Cardiovasc Res. 2006; 69:512–519.
64. Singer CA, Baker KJ, McCaffrey A, AuCoin DP, Dechert MA, Gerthoffer WT. p38 MAPK and NF-kappaB mediate COX-2 expression in human airway myocytes. Am J Physiol Lung Cell Mol Physiol. 2003; 285:L1087–1098.
65. Weber A, Wasiliew P and Kracht M. Interleukin-1 (IL-1) Pathway. Sci Signal. 2010; 3(105):-.
66. Hoffmann E, Dittrich-Breiholz O, Holtmann H, Kracht M. Multiple control of interleukin-8 gene expression. J Leukoc Biol. 2002; 72:847–855.
67. Zhu L, Crothers J Jr., Zhou R, Forte JG. A possible mechanism for ezrin to establish epithelial cell polarity. American journal of physiology Cell physiology. 2010; 299:C431–443.
68. Chang YJ, Wu MS, Lin JT, Sheu BS, Muta T, Inoue H, Chen CC. Induction of cyclooxygenase-2 overexpression in human gastric epithelial cells by Helicobacter pylori involves TLR2/TLR9 and c-Src-dependent nuclear factor-kappaB activation. Mol Pharmacol. 2004; 66:1465–1477.
69. Nakao S, Ogata Y, Shimizu-Sasaki E, Yamazaki M, Furuyama S, Sugiya H. Activation of NFkappaB is necessary for IL-1beta-induced cyclooxygenase-2 (COX-2) expression in human gingival fibroblasts. Mol Cell Biochem. 2000; 209:113–118.
70. Carmeliet P, Jain RK. Molecular mechanisms and clinical applications of angiogenesis. Nature. 2011; 473:298–307.
71. Gunaratnam L, Morley M, Franovic A, de Paulsen N, Mekhail K, Parolin DA, Nakamura E, Lorimer IA, Lee S. Hypoxia inducible factor activates the transforming growth factor-alpha/epidermal growth factor receptor growth stimulatory pathway in VHL(-/-) renal cell carcinoma cells. Journal of Biological Chemistry. 2003; 278:44966–44974.
72. Waugh DJ, Wilson C. The interleukin-8 pathway in cancer. Clin Cancer Res. 2008; 14:6735–6741.
73. Hatakeyama M, Imaizumi T, Sakaki H, Yoshida H, Tanaka H, Kimura H, Fukuda I, Satoh K. Interleukin-1 induces the expression of vascular endothelial growth factor in human pericardial mesothelial cells. Heart Vessels. 2007; 22:123–127.
74. Zhang YW, Su Y, Volpert OV, Vande Woude GF. Hepatocyte growth factor/scatter factor mediates angiogenesis through positive VEGF and negative thrombospondin 1 regulation. Proc Natl Acad Sci U S A. 2003; 100:12718–12723.
75. Giancotti FG, Ruoslahti E. Integrin signaling. Science. 1999; 285:1028–1032.
76. Skinner HD, Zheng JZ, Fang J, Agani F, Jiang BH. Vascular endothelial growth factor transcriptional activation is mediated by hypoxia-inducible factor 1alpha, HDM2, and p70S6K1 in response to phosphatidylinositol 3-kinase/AKT signaling. Journal of Biological Chemistry. 2004; 279:45643–45651.
77. Vander Heiden MG, Cantley LC, Thompson CB. Understanding the Warburg effect: the metabolic requirements of cell proliferation. Science. 2009; 324:1029–1033.
78. Engelman JA, Luo J, Cantley LC. The evolution of phosphatidylinositol 3-kinases as regulators of growth and metabolism. Nature reviews Genetics. 2006; 7:606–619.
79. Kaestner K. (2010). Development, Differentiation, and Disease of the Luminal Gastrointestinal Tract: Academic Press.
80. Unsicker K and Krieglstein K. (2006). Cell Signaling and Growth Factors in Development: Wiley.
81. Cavallaro U, Christofori G. Cell adhesion and signalling by cadherins and Ig-CAMs in cancer. Nat Rev Cancer. 2004; 4:118–132.
82. Thiery JP, Sleeman JP. Complex networks orchestrate epithelial-mesenchymal transitions. Nat Rev Mol Cell Biol. 2006; 7:131–142.
83. Silberg DG, Swain GP, Suh ER, Traber PG. Cdx1 and cdx2 expression during intestinal development. Gastroenterology. 2000; 119:961–971.
84. Gao N, White P, Kaestner KH. Establishment of intestinal identity and epithelial-mesenchymal signaling by Cdx2. Dev Cell. 2009; 16:588–599.
85. Henwood M, Clarke PA, Smith AM, Watson SA. Expression of gastrin in developing gastric adenocarcinoma. The British journal of surgery. 2001; 88:564–568.
86. Fourmy D, Gigoux V, Reubi JC. Gastrin in gastrointestinal diseases. Gastroenterology. 2011; 141:814–818. e811-813.
87. Mutoh H, Hakamata Y, Sato K, Eda A, Yanaka I, Honda S, Osawa H, Kaneko Y, Sugano K. Conversion of gastric

mucosa to intestinal metaplasia in Cdx2-expressing transgenic mice. Biochem Biophys Res Commun. 2002; 294:470–479.
88. Bai YQ, Yamamoto H, Akiyama Y, Tanaka H, Takizawa T, Koike M, Kenji Yagi O, Saitoh K, Takeshita K, Iwai T, Yuasa Y. Ectopic expression of homeodomain protein CDX2 in intestinal metaplasia and carcinomas of the stomach. Cancer Lett. 2002; 176:47–55.
89. Shu W, Guttentag S, Wang Z, Andl T, Ballard P, Lu MM, Piccolo S, Birchmeier W, Whitsett JA, Millar SE, Morrisey EE. Wnt/beta-catenin signaling acts upstream of N-myc, BMP4, and FGF signaling to regulate proximal-distal patterning in the lung. Dev Biol. 2005; 283:226–239.
90. Ebert MP, Fei G, Kahmann S, Muller O, Yu J, Sung JJ, Malfertheiner P. Increased beta-catenin mRNA levels and mutational alterations of the APC and beta-catenin gene are present in intestinal-type gastric cancer. Carcinogenesis. 2002; 23:87–91.
91. Yuasa Y. Control of gut differentiation and intestinal-type gastric carcinogenesis. Nat Rev Cancer. 2003; 3:592–600.
92. Que J, Okubo T, Goldenring JR, Nam KT, Kurotani R, Morrisey EE, Taranova O, Pevny LH, Hogan BL. Multiple dose-dependent roles for Sox2 in the patterning and differentiation of anterior foregut endoderm. Development. 2007; 134:2521–2531.
93. Arnold K, Sarkar A, Yram MA, Polo JM, Bronson R, Sengupta S, Seandel M, Geijsen N, Hochedlinger K. Sox2(+) adult stem and progenitor cells are important for tissue regeneration and survival of mice. Cell Stem Cell. 2011; 9:317–329.
94. Tani Y, Akiyama Y, Fukamachi H, Yanagihara K, Yuasa Y. Transcription factor SOX2 up-regulates stomach-specific pepsinogen A gene expression. J Cancer Res Clin Oncol. 2007; 133:263–269.
95. Park ET, Gum JR, Kakar S, Kwon SW, Deng G, Kim YS. Aberrant expression of SOX2 upregulates MUC5AC gastric foveolar mucin in mucinous cancers of the colorectum and related lesions. Int J Cancer. 2008; 122:1253–1260.
96. Raghoebir L, Bakker ER, Mills JC, Swagemakers S, Kempen MB, Munck AB, Driegen S, Meijer D, Grosveld F, Tibboel D, Smits R, Rottier RJ. SOX2 redirects the developmental fate of the intestinal epithelium toward a premature gastric phenotype. J Mol Cell Biol. 2012; 4:377–385.
97. Wang TC, Fox JG and Giraud AS. (2009). The Biology of Gastric Cancers: Springer.
98. Rozakis-Adcock M, Fernley R, Wade J, Pawson T, Bowtell D. The SH2 and SH3 domains of mammalian Grb2 couple the EGF receptor to the Ras activator mSos1. Nature. 1993; 363:83–85.
99. Gherardi E, Birchmeier W, Birchmeier C, Vande Woude G. Targeting MET in cancer: rationale and progress. Nat Rev Cancer. 2012; 12:89–103.
100. Ferrara N, Gerber HP, LeCouter J. The biology of VEGF and its receptors. Nat Med. 2003; 9:669–676.
101. Eliceiri BP. Integrin and growth factor receptor crosstalk. Circ Res. 2001; 89:1104–1110.
102. Stupack DG, Cheresh DA. Get a ligand, get a life: integrins, signaling and cell survival. J Cell Sci. 2002; 115:3729–3738.
103. Pylayeva-Gupta Y, Grabocka E, Bar-Sagi D. RAS oncogenes: weaving a tumorigenic web. Nat Rev Cancer. 2011; 11:761–774.
104. Zhu J, Blenis J, Yuan J. Activation of PI3K/Akt and MAPK pathways regulates Myc-mediated transcription by phosphorylating and promoting the degradation of Mad1. Proc Natl Acad Sci U S A. 2008; 105:6584–6589.
105. Abbas T, Dutta A. p21 in cancer: intricate networks and multiple activities. Nat Rev Cancer. 2009; 9:400–414.
106. Gambarotta G, Boccaccio C, Giordano S, Ando M, Stella MC, Comoglio PM. Ets up-regulates MET transcription. Oncogene. 1996; 13:1911–1917.
107. Datta SR, Dudek H, Tao X, Masters S, Fu H, Gotoh Y, Greenberg ME. Akt phosphorylation of BAD couples survival signals to the cell-intrinsic death machinery. Cell. 1997; 91:231–241.
108. Fujita E, Jinbo A, Matuzaki H, Konishi H, Kikkawa U, Momoi T. Akt phosphorylation site found in human caspase-9 is absent in mouse caspase-9. Biochem Biophys Res Commun. 1999; 264:550–555.
109. Zhou H, Li XM, Meinkoth J, Pittman RN. Akt regulates cell survival and apoptosis at a postmitochondrial level. J Cell Biol. 2000; 151:483–494.
110. Zhou BP, Liao Y, Xia W, Spohn B, Lee MH, Hung MC. Cytoplasmic localization of p21Cip1/WAF1 by Akt-induced phosphorylation in HER-2/neu-overexpressing cells. Nat Cell Biol. 2001; 3:245–252.
111. Cohen P, Frame S. The renaissance of GSK3. Nat Rev Mol Cell Biol. 2001; 2:769–776.
112. Waite KA, Eng C. Protean PTEN. form and function. Am J Hum Genet. 2002; 70:829–844.
113. Katsuda K, Kataoka M, Uno F, Murakami T, Kondo T, Roth JA, Tanaka N, Fujiwara T. Activation of caspase-3 and cleavage of Rb are associated with p16-mediated apoptosis in human non-small cell lung cancer cells. Oncogene. 2002; 21:2108–2113.
114. Takahashi-Yanaga F, Sasaguri T. GSK-3beta regulates cyclin D1 expression: a new target for chemotherapy. Cell Signal. 2008; 20:581–589.
115. Oswald F, Lovec H, Moroy T, Lipp M. E2F-dependent regulation of human MYC: trans-activation by cyclins D1 and A overrides tumour suppressor protein functions. Oncogene. 1994; 9:2029–2036.
116. Chen CR, Kang Y, Siegel PM, Massagué J. E2F4/5 and p107 as Smad cofactors linking the TGF receptor to c-myc repression. Cell. 2002; 110:19–32. [beta].

117. Alexandrow MG, Moses HL. Transforming growth factor beta and cell cycle regulation. Cancer Research. 1995; 55:1452–1457.

118. Vairo G, Vadiveloo PK, Royston AK, Rockman SP, Rock CO, Jackowski S, Hamilton JA. Deregulated c-myc expression overrides IFN gamma-induced macrophage growth arrest. Oncogene. 1995; 10:1969–1976.

119. Ayer DE, Eisenman RN. A switch from Myc:Max to Mad:Max heterocomplexes accompanies monocyte/macrophage differentiation. Genes Dev. 1993; 7:2110–2119.

120. Ayer DE, Kretzner L, Eisenman RN. Mad: a heterodimeric partner for Max that antagonizes Myc transcriptional activity. Cell. 1993; 72:211–222.

121. Zhang YW, Su Y, Volpert OV, Woude GFV. Hepatocyte growth factor/scatter factor mediates angiogenesis through positive VEGF and negative thrombospondin 1 regulation. Proceedings of the National Academy of Sciences. 2003; 100:12718.

122. Kitaura H, Shinshi M, Uchikoshi Y, Ono T, Iguchi-Ariga SM, Ariga H. Reciprocal regulation via protein-protein interaction between c-Myc and p21(cip1/waf1/sdi1) in DNA replication and transcription. Journal of Biological Chemistry. 2000; 275:10477–10483.

123. He TC, Sparks AB, Rago C, Hermeking H, Zawel L, da Costa LT, Morin PJ, Vogelstein B, Kinzler KW. Identification of c-MYC as a target of the APC pathway. Science. 1998; 281:1509–1512.

124. Engelmann D, Putzer BM. The dark side of E2F1: in transit beyond apoptosis. Cancer Research. 2012; 72:571–575.

125. Dyson N. The regulation of E2F by pRB-family proteins. Gene Dev. 1998; 12:2245–2262.

126. Harbour JW, Dean DC. The Rb/E2F pathway: expanding roles and emerging paradigms. Gene Dev. 2000; 14:2393–2409.

127. Sears R, Ohtani K, Nevins JR. Identification of positively and negatively acting elements regulating expression of the E2F2 gene in response to cell growth signals. Mol Cell Biol. 1997; 17:5227–5235.

128. Dimri GP, Nakanishi M, Desprez PY, Smith JR, Campisi J. Inhibition of E2F activity by the cyclin-dependent protein kinase inhibitor p21 in cells expressing or lacking a functional retinoblastoma protein. Molecular and Cellular Biology. 1996; 16:2987–2997.

129. Afshari CA, Nichols MA, Xiong Y, Mudryj M. A role for a p21-E2F interaction during senescence arrest of normal human fibroblasts. Cell Growth Differ. 1996; 7:979–988.

130. Hiyama H, Iavarone A, Reeves SA. Regulation of the cdk inhibitor p21 gene during cell cycle progression is under the control of the transcription factor E2F. Oncogene. 1998; 16:1513–1523.

131. Radhakrishnan SK, Feliciano CS, Najmabadi F, Haegebarth A, Kandel ES, Tyner AL, Gartel AL. Constitutive expression of E2F-1 leads to p21-dependent cell cycle arrest in S phase of the cell cycle. Oncogene. 2004; 23:4173–4176.

132. Koshiji M, Kageyama Y, Pete EA, Horikawa I, Barrett JC, Huang LE. HIF-1alpha induces cell cycle arrest by functionally counteracting Myc. EMBO J. 2004; 23:1949–1956.

133. Xaus J, Cardo M, Valledor AF, Soler C, Lloberas J, Celada A. Interferon gamma induces the expression of p21waf-1 and arrests macrophage cell cycle, preventing induction of apoptosis. Immunity. 1999; 11:103–113.

134. Kominsky S, Johnson HM, Bryan G, Tanabe T, Hobeika AC, Subramaniam PS, Torres B. IFNgamma inhibition of cell growth in glioblastomas correlates with increased levels of the cyclin dependent kinase inhibitor p21WAF1/CIP1. Oncogene. 1998; 17:2973–2979.

135. Mandal M, Bandyopadhyay D, Goepfert TM, Kumar R. Interferon-induces expression of cyclin-dependent kinase-inhibitors p21WAF1 and p27Kip1 that prevent activation of cyclin-dependent kinase by CDK-activating kinase (CAK). Oncogene. 1998; 16:217–225.

136. Gottschalk AR, Basila D, Wong M, Dean NM, Brandts CH, Stokoe D, Haas-Kogan DA. p27Kip1 is required for PTEN-induced G1 growth arrest. Cancer Research. 2001; 61:2105–2111.

137. St Croix B, Sheehan C, Rak JW, Florenes VA, Slingerland JM, Kerbel RS. E-Cadherin-dependent growth suppression is mediated by the cyclin-dependent kinase inhibitor p27(KIP1). J Cell Biol. 1998; 142:557–571.

138. Sa G, Stacey DW. P27 expression is regulated by separate signaling pathways, downstream of Ras, in each cell cycle phase. Exp Cell Res. 2004; 300:427–439.

139. Harvat BL, Seth P, Jetten AM. The role of p27Kip1 in gamma interferon-mediated growth arrest of mammary epithelial cells and related defects in mammary carcinoma cells. Oncogene. 1997; 14:2111–2122.

140. Matsuoka M, Nishimoto I, Asano S. Interferon-gamma impairs physiologic downregulation of cyclin-dependent kinase inhibitor, pKip1, during G1 phase progression in macrophages. Exp Hematol. 1999; 27:203–209.

141. Sheaff RJ, Groudine M, Gordon M, Roberts JM, Clurman BE. Cyclin E-CDK2 is a regulator of p27Kip1. Genes Dev. 1997; 11:1464–1478.

142. Ciarallo S, Subramaniam V, Hung W, Lee J-H, Kotchetkov R, Sandhu C, Milic A, Slingerland JM. Altered p27Kip1 phosphorylation, localization, and function in human epithelial cells resistant to transforming growth factor β-mediated G1 arrest. Mol Cell Biol. 2002; 22:2993–3002.

143. Rivard N, Boucher MJ, Asselin C, L'Allemain G. MAP kinase cascade is required for p27 downregulation and S phase entry in fibroblasts and epithelial cells. Am J Physiol-Cell Ph. 1999; 277:C652–C664.

144. Quante M, Varga J, Wang TC, Greten FR. The gastrointestinal tumor microenvironment. Gastroenterology. 2013; 145:63–78.

145. Sherr CJ. The INK4a/ARF network in tumour suppression. Nat Rev Mol Cell Bio. 2001; 2:731–737.

146. Sherr CJ, Weber JD. The ARF/p53 pathway. Curr Opin Genet Dev. 2000; 10:94–99.

147. Evan GI, Wyllie AH, Gilbert CS, Littlewood TD, Land H, Brooks M, Waters CM, Penn LZ, Hancock DC. Induction of apoptosis in fibroblasts by c-myc protein. Cell. 1992; 69:119–128.

148. Reisman D, Elkind NB, Roy B, Beamon J, Rotter V. c-Myc trans-activates the p53 promoter through a required downstream CACGTG motif. Cell Growth Differ. 1993; 4:57–65.

149. Cregan SP, Dawson VL, Slack RS. Role of AIF in caspase-dependent and caspase-independent cell death. Oncogene. 2004; 23:2785–2796.

150. Suzuki A, Tsutomi Y, Akahane K, Araki T, Miura M. Resistance to Fas-mediated apoptosis: activation of caspase 3 is regulated by cell cycle regulator p21WAF1 and IAP gene family ILP. Oncogene. 1998; 17:931–939.

151. Suzuki A, Tsutomi Y, Yamamoto N, Shibutani T, Akahane K. Mitochondrial regulation of cell death: mitochondria are essential for procaspase 3-p21 complex formation to resist Fas-mediated cell death. Molecular and Cellular Biology. 1999; 19:3842–3847.

152. Moroni MC, Hickman ES, Lazzerini Denchi E, Caprara G, Colli E, Cecconi F, Muller H, Helin K. Apaf-1 is a transcriptional target for E2F and p5. Nat Cell Biol. 2001; 3:552–558.

153. Furukawa Y, Nishimura N, Satoh M, Endo H, Iwase S, Yamada H, Matsuda M, Kano Y, Nakamura M. Apaf-1 is a mediator of E2F-1-induced apoptosis. Journal of Biological Chemistry. 2002; 277:39760–39768.

154. Janssens S, Beyaert R. A universal role for MyD88 in TLR/IL-1R-mediated signaling. Trends Biochem Sci. 2002; 27:474–482.

155. Dan HC, Sun M, Kaneko S, Feldman RI, Nicosia SV, Wang HG, Tsang BK, Cheng JQ. Akt phosphorylation and stabilization of X-linked inhibitor of apoptosis protein (XIAP). Journal of Biological Chemistry. 2004; 279:5405–5412.

156. Bufalo DD, Trisciuoglio D, Milella M. Crosstalk between VEGF and Bcl-2 in Tumor Progression and Angiogenesis. VEGF and Cancer. 2004:26–39.

157. Rahimi RA, Leof EB. TGF-beta signaling: a tale of two responses. J Cell Biochem. 2007; 102:593–608.

158. Breitschopf K, Haendeler J, Malchow P, Zeiher AM, Dimmeler S. Posttranslational modification of Bcl-2 facilitates its proteasome-dependent degradation: molecular characterization of the involved signaling pathway. Molecular and Cellular Biology. 2000; 20:1886–1896.

159. Hoffman B, Liebermann DA. Apoptotic signaling by c-MYC. Oncogene. 2008; 27:6462–6472.

160. Rodeck U, Jost M, Kari C, Shih DT, Lavker RM, Ewert DL, Jensen PJ. EGF-R dependent regulation of keratinocyte survival. J Cell Sci. 1997; 110:113–121.

161. Rodeck U, Jost M, DuHadaway J, Kari C, Jensen PJ, Risse B, Ewert DL. Regulation of Bcl-xL expression in human keratinocytes by cell-substratum adhesion and the epidermal growth factor receptor. Proc Natl Acad Sci U S A. 1997; 94:5067–5072.

162. Stoll SW, Benedict M, Mitra R, Hiniker A, Elder JT, Nunez G. EGF receptor signaling inhibits keratinocyte apoptosis: evidence for mediation by Bcl-XL. Oncogene. 1998; 16:1493–1499.

163. Jost M, Huggett TM, Kari C, Boise LH, Rodeck U. Epidermal growth factor receptor-dependent control of keratinocyte survival and Bcl-xL expression through a MEK-dependent pathway. Journal of Biological Chemistry. 2001; 276:6320–6326.

164. Parrizas M, LeRoith D. Insulin-like growth factor-1 inhibition of apoptosis is associated with increased expression of the bcl-xL gene product. Endocrinology. 1997; 138:1355–1358.

165. Sekharam M, Zhao H, Sun M, Fang Q, Zhang Q, Yuan Z, Dan HC, Boulware D, Cheng JQ, Coppola D. Insulin-like growth factor 1 receptor enhances invasion and induces resistance to apoptosis of colon cancer cells through the Akt/Bcl-x(L) pathway. Cancer Research. 2003; 63:7708–7716.

166. Jiang P, Du W, Wu M. p53 and Bad: remote strangers become close friends. Cell Res. 2007; 17:283–285.

167. She QB, Solit DB, Ye Q, O'Reilly KE, Lobo J, Rosen N. The BAD protein integrates survival signaling by EGFR/MAPK and PI3K/Akt kinase pathways in PTEN-deficient tumor cells. Cancer Cell. 2005; 8:287–297.

168. Zhang Z, Lapolla SM, Annis MG, Truscott M, Roberts GJ, Miao Y, Shao Y, Tan C, Peng J, Johnson AE, Zhang XC, Andrews DW, Lin J. Bcl-2 homodimerization involves two distinct binding surfaces, a topographic arrangement that provides an effective mechanism for Bcl-2 to capture activated Bax. Journal of Biological Chemistry. 2004; 279:43920–43928.

169. Peluso JJ, Pappalardo A, Fernandez G. E-cadherin-mediated cell contact prevents apoptosis of spontaneously immortalized granulosa cells by regulating Akt kinase activity. Biol Reprod. 2001; 64:1183–1190.

170. Guo W, Giancotti FG. Integrin signalling during tumour progression. Nat Rev Mol Cell Biol. 2004; 5:816–826.

171. Graziani A, Gramaglia D, Cantley LC, Comoglio PM. The tyrosine-phosphorylated hepatocyte growth factor/scatter factor receptor associates with phosphatidylinositol 3-kinase. Journal of Biological Chemistry. 1991; 266:22087–22090.
172. Ponzetto C, Bardelli A, Maina F, Longati P, Panayotou G, Dhand R, Waterfield MD, Comoglio PM. A novel recognition motif for phosphatidylinositol 3-kinase binding mediates its association with the hepatocyte growth factor/scatter factor receptor. Molecular and Cellular Biology. 1993; 13:4600–4608.
173. Citri A, Yarden Y. EGF-ERBB signalling: towards the systems level. Nat Rev Mol Cell Biol. 2006; 7:505–516.
174. Yu H, Rohan T. Role of the insulin-like growth factor family in cancer development and progression. J Natl Cancer Inst. 2000; 92:1472–1489.
175. LeRoith D, Roberts CT Jr. The insulin-like growth factor system and cancer. Cancer Lett. 2003; 195:127–137.
176. Treins C, Giorgetti-Peraldi S, Murdaca J, Semenza GL, Van Obberghen E. Insulin stimulates hypoxia-inducible factor 1 through a phosphatidylinositol 3-kinase/target of rapamycin-dependent signaling pathway. Journal of Biological Chemistry. 2002; 277:27975–27981.
177. Achyut BR, Yang L. Transforming growth factor-beta in the gastrointestinal and hepatic tumor microenvironment. Gastroenterology. 2011; 141:1167–1178.
178. Wang H, Quah SY, Dong JM, Manser E, Tang JP, Zeng Q. PRL-3 down-regulates PTEN expression and signals through PI3K to promote epithelial-mesenchymal transition. Cancer Res. 2007; 67:2922–2926.
179. Hara S, Oya M, Mizuno R, Horiguchi A, Marumo K, Murai M. Akt activation in renal cell carcinoma: contribution of a decreased PTEN expression and the induction of apoptosis by an Akt inhibitor. Ann Oncol. 2005; 16:928–933.
180. Vasudevan KM, Gurumurthy S, Rangnekar VM. Suppression of PTEN expression by NF-kappa B prevents apoptosis. Molecular and Cellular Biology. 2004; 24:1007–1021.
181. Lau MT, Klausen C, Leung PC. E-cadherin inhibits tumor cell growth by suppressing PI3K/Akt signaling via beta-catenin-Egr1-mediated PTEN expression. Oncogene. 2011; 30:2753–2766.
182. Ferrand A, Wang TC. Gastrin and cancer: a review. Cancer Lett. 2006; 238:15–29.
183. Liu Y, Shepherd EG, Nelin LD. MAPK phosphatases–regulating the immune response. Nat Rev Immunol. 2007; 7:202–212.
184. Saraiva M, O'Garra A. The regulation of IL- production by immune cells. Nat Rev Immunol. 2010; 10:170–181.
185. Hocevar BA, Brown TL, Howe PH. TGF-beta induces fibronectin synthesis through a c-Jun N-terminal kinase-dependent, Smad4-independent pathway. EMBO J. 1999; 18:1345–1356.
186. Engel ME, McDonnell MA, Law BK, Moses HL. Interdependent SMAD and JNK signaling in transforming growth factor-beta-mediated transcription. Journal of Biological Chemistry. 1999; 274:37413–37420.
187. Kant S, Swat W, Zhang S, Zhang ZY, Neel BG, Flavell RA, Davis RJ. TNF-stimulated MAP kinase activation mediated by a Rho family GTPase signaling pathway. Genes Dev. 2011; 25:2069–2078.
188. Zhao Y, Zhang ZY. The mechanism of dephosphorylation of extracellular signal-regulated kinase 2 by mitogen-activated protein kinase phosphatase 3. Journal of Biological Chemistry. 2001; 276:32382–32391.
189. Chi H, Barry SP, Roth RJ, Wu JJ, Jones EA, Bennett AM, Flavell RA. Dynamic regulation of pro-and anti-inflammatory cytokines by MAPK phosphatase 1 (MKP-1) in innate immune responses. P Natl Acad Sci USA. 2006; 103:2274.
190. Pearson G, Robinson F, Beers Gibson T, Xu BE, Karandikar M, Berman K, Cobb MH. Mitogen-activated protein (MAP) kinase pathways: regulation and physiological functions. Endocr Rev. 2001; 22:153–183.
191. Brondello JM, Pouysségur J, McKenzie FR. Reduced MAP kinase phosphatase-1 degradation after p42/p44MAPK-dependent phosphorylation. Science. 1999; 286:2514.
192. Hu J-H, Chen T, Zhuang Z-H, Kong L, Yu M-C, Liu Y, Zang J-W, Ge B-X. Feedback control of MKP-1 expression by p38. Cell Signal. 2007; 19:393–400.
193. Iniguez MA, Rodriguez A, Volpert OV, Fresno M, Redondo JM. Cyclooxygenase-2: a therapeutic target in angiogenesis. Trends in Molecular Medicine. 2003; 9:73–78.
194. Niu G, Wright KL, Huang M, Song L, Haura E, Turkson J, Zhang S, Wang T, Sinibaldi D, Coppola D, Heller R, Ellis LM, Karras J, Bromberg J, Pardoll D, Jove R, et al. Constitutive Stat3 activity up-regulates VEGF expression and tumor angiogenesis. Oncogene. 2002; 21:2000–2008.
195. Sulpice E, Ding S, Muscatelli-Groux B, Berge M, Han ZC, Plouet J, Tobelem G, Merkulova-Rainon T. Cross-talk between the VEGF-A and HGF signalling pathways in endothelial cells. Biol Cell. 2009; 101:525–539.
196. Pennacchietti S, Michieli P, Galluzzo M, Mazzone M, Giordano S, Comoglio PM. Hypoxia promotes invasive growth by transcriptional activation of the met protooncogene. Cancer Cell. 2003; 3:347–361.
197. Boccaccio C, Comoglio PM. Invasive growth: a MET-driven genetic programme for cancer and stem cells. Nat Rev Cancer. 2006; 6:637–645.
198. Boon EM, van der Neut R, van de Wetering M, Clevers H, Pals ST. Wnt signaling regulates expression of the receptor tyrosine kinase met in colorectal cancer. Cancer Research. 2002; 62:5126–5128.
199. Mariotti A, Kedeshian PA, Dans M, Curatola AM, Gagnoux-Palacios L, Giancotti FG. EGF-R signaling through Fyn kinase disrupts the function of integrin

alpha6beta4 at hemidesmosomes: role in epithelial cell migration and carcinoma invasion. J Cell Biol. 2001; 155:447–458.

200. Chan PC, Chen SY, Chen CH, Chen HC. Crosstalk between hepatocyte growth factor and integrin signaling pathways. J Biomed Sci. 2006; 13:215–223.

201. Desgrosellier JS, Cheresh DA. Integrins in cancer: biological implications and therapeutic opportunities. Nat Rev Cancer. 2010; 10:9–22.

202. Trusolino L, Bertotti A, Comoglio PM. A signaling adapter function for alpha6beta4 integrin in the control of HGF-dependent invasive growth. Cell. 2001; 107:643–654.

203. Avizienyte E, Wyke AW, Jones RJ, McLean GW, Westhoff MA, Brunton VG, Frame MC. Src-induced de-regulation of E-cadherin in colon cancer cells requires integrin signalling. Nat Cell Biol. 2002; 4:632–638.

204. Chan AO, Huang C, Hui WM, Cho CH, Yuen MF, Lam SK, Rashid A, Wong BC. Stability of E-cadherin methylation status in gastric mucosa associated with histology changes. Aliment Pharmacol Ther. 2006; 24:831–836.

205. Steinhusen U, Weiske J, Badock V, Tauber R, Bommert K, Huber O. Cleavage and shedding of E-cadherin after induction of apoptosis. Journal of Biological Chemistry. 2001; 276:4972–4980.

206. Pece S, Gutkind JS. E-cadherin and Hakai: signalling, remodeling or destruction?. Nat Cell Biol. 2002; 4:E72–74.

207. Kamei T, Matozaki T, Sakisaka T, Kodama A, Yokoyama S, Peng YF, Nakano K, Takaishi K, Takai Y. Coendocytosis of cadherin and c-Met coupled to disruption of cell-cell adhesion in MDCK cells–regulation by Rho, Rac and Rab small G proteins. Oncogene. 1999; 18:6776–6784.

208. Davies G, Jiang WG, Mason MD. HGF/SF modifies the interaction between its receptor c-Met, and the E-cadherin/catenin complex in prostate cancer cells. Int J Mol Med. 2001; 7:385–388.

209. Comijn J, Berx G, Vermassen P, Verschueren K, van Grunsven L, Bruyneel E, Mareel M, Huylebroeck D, van Roy F. The two-handed E box binding zinc finger protein SIP1 downregulates E-cadherin and induces invasion. Mol Cell. 2001; 7:1267–1278.

210. Eger A, Aigner K, Sonderegger S, Dampier B, Oehler S, Schreiber M, Berx G, Cano A, Beug H, Foisner R. DeltaEF1 is a transcriptional repressor of E-cadherin and regulates epithelial plasticity in breast cancer cells. Oncogene. 2005; 24:2375–2385.

211. Chua HL, Bhat-Nakshatri P, Clare SE, Morimiya A, Badve S, Nakshatri H. NF-kappaB represses E-cadherin expression and enhances epithelial to mesenchymal transition of mammary epithelial cells: potential involvement of ZEB-1 and ZEB-2. Oncogene. 2007; 26:711–724.

212. Ruzinova MB, Benezra R. Id proteins in development, cell cycle and cancer. Trends Cell Biol. 2003; 13:410–418.

213. Chang CJ, Chao CH, Xia W, Yang JY, Xiong Y, Li CW, Yu WH, Rehman SK, Hsu JL, Lee HH. p53 regulates epithelial-mesenchymal transition and stem cell properties through modulating miRNAs. Nature cell biology. 2011; 13:317–323.

214. Park SM, Gaur AB, Lengyel E, Peter ME. The miR-200 family determines the epithelial phenotype of cancer cells by targeting the E-cadherin repressors ZEB1 and ZEB2. Genes Dev. 2008; 22:894–907.

215. Song DH, Kaufman JC, Borodyansky L, Albanese C, Pestell RG, Wolfe MM. Gastrin stabilises beta-catenin protein in mouse colorectal cancer cells. Br J Cancer. 2005; 92:1581–1587.

216. Nelson WJ, Nusse R. Convergence of Wnt, beta-catenin, and cadherin pathways. Science. 2004; 303:1483–1487.

217. Mansukhani A, Ambrosetti D, Holmes G, Cornivelli L, Basilico C. Sox2 induction by FGF and FGFR2 activating mutations inhibits Wnt signaling and osteoblast differentiation. J Cell Biol. 2005; 168:1065–1076.

218. Danilkovitch-Miagkova A, Miagkov A, Skeel A, Nakaigawa N, Zbar B, Leonard EJ. Oncogenic mutants of RON and MET receptor tyrosine kinases cause activation of the beta-catenin pathway. Molecular and Cellular Biology. 2001; 21:5857–5868.

219. Vivanco I, Sawyers CL. The phosphatidylinositol 3-kinase-AKT pathway in human cancer. Nature Reviews Cancer. 2002; 2:489–501.

220. Scheid MP, Woodgett JR. PKB/AKT: Functional insights from genetic models. Nat Rev Mol Cell Bio. 2001; 2:760–768.

221. Conacci-Sorrell M, Simcha I, Ben-Yedidia T, Blechman J, Savagner P, Ben-Ze'ev A. Autoregulation of E-cadherin expression by cadherin-cadherin interactions: the roles of beta-catenin signaling, Slug, and MAPK. J Cell Biol. 2003; 163:847–857.

222. Morali OG, Delmas V, Moore R, Jeanney C, Thiery JP, Larue L. IGF-II induces rapid beta-catenin relocation to the nucleus during epithelium to mesenchyme transition. Oncogene. 2001; 20:4942–4950.

223. Playford MP, Bicknell D, Bodmer WF, Macaulay VM. Insulin-like growth factor 1 regulates the location, stability, and transcriptional activity of beta-catenin. Proc Natl Acad Sci U S A. 2000; 97:12103–12108.

224. Laughner E, Taghavi P, Chiles K, Mahon PC, Semenza GL. HER2 (neu) signaling increases the rate of hypoxia-inducible factor 1alpha (HIF-1alpha) synthesis: novel mechanism for HIF-1-mediated vascular endothelial growth factor expression. Molecular and Cellular Biology. 2001; 21:3995–4004.

225. Ravi R, Mookerjee B, Bhujwalla ZM, Sutter CH, Artemov D, Zeng Q, Dillehay LE, Madan A, Semenza GL, Bedi A. Regulation of tumor angiogenesis by p53-induced degradation of hypoxia-inducible factor 1alpha. Genes Dev. 2000; 14:34–44.

226. Belaiba RS, Bonello S, Zahringer C, Schmidt S, Hess J, Kietzmann T, Gorlach A. Hypoxia up-regulates hypoxia-inducible factor-1alpha transcription by involving phosphatidylinositol 3-kinase and nuclear factor kappaB in pulmonary artery smooth muscle cells. Mol Biol Cell. 2007; 18:4691–4697.
227. Gorlach A, Bonello S. The cross-talk between NF-kappaB and HIF-1: further evidence for a significant liaison. Biochem J. 2008; 412:e17–19.
228. Finco TS, Westwick JK, Norris JL, Beg AA, Der CJ, Baldwin AS Jr. Oncogenic Ha-Ras-induced signaling activates NF-kappaB transcriptional activity, which is required for cellular transformation. Journal of Biological Chemistry. 1997; 272:24113–24116.
229. Zandi E, Karin M. Bridging the gap: composition, regulation, and physiological function of the IkappaB kinase complex. Molecular and Cellular Biology. 1999; 19:4547–4551.
230. Driessler F, Venstrom K, Sabat R, Asadullah K, Schottelius AJ. Molecular mechanisms of interleukin-10-mediated inhibition of NF-kappaB activity: a role for p50. Clin Exp Immunol. 2004; 135:64–73.
231. Murray PJ. Understanding and exploiting the endogenous interleukin-10/STAT3-mediated anti-inflammatory response. Curr Opin Pharmacol. 2006; 6:379–386.
232. Sun SC, Ganchi PA, Ballard DW, Greene WC. NF-kappa B controls expression of inhibitor I kappa B alpha: evidence for an inducible autoregulatory pathway. Science. 1993; 259:1912–1915.
233. Griffin BD, Moynagh PN. In vivo binding of NF-kappa B to the I kappa B beta promoter is insufficient for transcriptional activation. Biochemical Journal. 2006; 400:115–125.
234. Windheim M, Stafford M, Peggie M, Cohen P. Interleukin-1 (IL-1) induces the Lys63-linked polyubiquitination of IL-1 receptor-associated kinase 1 to facilitate NEMO binding and the activation of IkappaBalpha kinase. Molecular and Cellular Biology. 2008; 28:1783–1791.
235. Romashkova JA, Makarov SS. NF-kappaB is a target of AKT in anti-apoptotic PDGF signalling. Nature. 1999; 401:86–90.
236. Madrid LV, Wang CY, Guttridge DC, Schottelius AJ, Baldwin AS Jr., Mayo MW. Akt suppresses apoptosis by stimulating the transactivation potential of the RelA/p65 subunit of NF-kappaB. Molecular and Cellular Biology. 2000; 20:1626–1638.
237. Burow ME, Weldon CB, Melnik LI, Duong BN, Collins-Burow BM, Beckman BS, McLachlan JA. PI3-K/AKT regulation of NF-kappaB signaling events in suppression of TNF-induced apoptosis. Biochem Biophys Res Commun. 2000; 271:342–345.
238. Meng F, Liu L, Chin PC, D’Mello SR. Akt is a downstream target of NF-kappa, B. Journal of Biological Chemistry. 2002; 277:29674–29680.
239. Schneider P, Thome M, Burns K, Bodmer JL, Hofmann K, Kataoka T, Holler N, Tschopp J. TRAIL receptors 1 (DR4) and 2 (DR5) signal FADD-dependent apoptosis and activate NF-kappaB. Immunity. 1997; 7:831–836.
240. Derynck R, Zhang YE. Smad-dependent and Smad-independent pathways in TGF-beta family signalling. Nature. 2003; 425:577–584.
241. Rajasingh J, Bord E, Luedemann C, Asai J, Hamada H, Thorne T, Qin G, Goukassian D, Zhu Y, Losordo DW, Kishore R. IL-10-induced TNF-alpha mRNA destabilization is mediated via IL-10 suppression of p38 MAP kinase activation and inhibition of HuR expression. Faseb Journal. 2006; 20:2112–2114.
242. Rutault K, Hazzalin CA, Mahadevan LC. Combinations of ERK and p38 MAPK inhibitors ablate tumor necrosis factor-alpha (TNF-alpha) mRNA induction. Evidence for selective destabilization of TNF-alpha transcripts. Journal of Biological Chemistry. 2001; 276:6666–6674.
243. Foey AD, Parry SL, Williams LM, Feldmann M, Foxwell BM, Brennan FM. Regulation of monocyte IL-10 synthesis by endogenous IL-1 and TNF-alpha: role of the p38 and p42/44 mitogen-activated protein kinases. J Immunol. 1998; 160:920–928.
244. Hu X, Paik PK, Chen J, Yarilina A, Kockeritz L, Lu TT, Woodgett JR, Ivashkiv LB. IFN-gamma suppresses IL-10 production and synergizes with TLR2 by regulating GSK3 and CREB/AP-1 proteins. Immunity. 2006; 24:563–574.
245. Walker PR, Saas P, Dietrich PY. Role of Fas ligand (CD95L) in immune escape: the tumor cell strikes back. J Immunol. 1997; 158:4521–4524.
246. Ferguson TA, Griffith TS. A vision of cell death: Fas ligand and immune privilege 10 years later. Immunological Reviews. 2006; 213:228–238.
247. Dinarello CA, Ikejima T, Warner SJ, Orencole SF, Lonnemann G, Cannon JG, Libby P. Interleukin 1 induces interleukin 1.I. Induction of circulating interleukin 1 in rabbits in vivo and in human mononuclear cells *in vitro*. J Immunol. 1987; 139:1902–1910.
248. Brissoni B, Agostini L, Kropf M, Martinon F, Swoboda V, Lippens S, Everett H, Aebi N, Janssens S, Meylan E, Felberbaum-Corti M, Hirling H, Gruenberg J, Tschopp J, Burns K. Intracellular trafficking of interleukin-1 receptor I requires Tollip. Curr Biol. 2006; 16:2265–2270.
249. Burns K, Clatworthy J, Martin L, Martinon F, Plumpton C, Maschera B, Lewis A, Ray K, Tschopp J, Volpe F. Tollip, a new component of the IL-1RI pathway, links IRAK to the IL-1 receptor. Nat Cell Biol. 2000; 2:346–351.
250. Didierlaurent A, Brissoni B, Velin D, Aebi N, Tardivel A, Kaslin E, Sirard JC, Angelov G, Tschopp J, Burns K. Tollip regulates proinflammatory responses to interleukin-1 and lipopolysaccharide. Molecular and Cellular Biology. 2006; 26:735–742.
251. Peli J, Schroter M, Rudaz C, Hahne M, Meyer C, Reichmann E, Tschopp J. Oncogenic Ras inhibits Fas

ligand-mediated apoptosis by downregulating the expression of Fas. EMBO J. 1999; 18:1824–1831.
252. Xu X, Fu XY, Plate J, Chong AS. IFN-gamma induces cell growth inhibition by Fas-mediated apoptosis: requirement of STAT1 protein for up-regulation of Fas and FasL expression. Cancer Research. 1998; 58:2832–2837.
253. Munger JS, Huang X, Kawakatsu H, Griffiths MJ, Dalton SL, Wu J, Pittet JF, Kaminski N, Garat C, Matthay MA, Rifkin DB, Sheppard D. The integrin alpha v beta 6 binds and activates latent TGF beta 1: a mechanism for regulating pulmonary inflammation and fibrosis. Cell. 1999; 96:319–328.
254. Mu D, Cambier S, Fjellbirkeland L, Baron JL, Munger JS, Kawakatsu H, Sheppard D, Broaddus VC, Nishimura SL. The integrin alpha(v)beta8 mediates epithelial homeostasis through MT1-MMP-dependent activation of TGF-beta1. J Cell Biol. 2002; 157:493–507.
255. Bitzer M, von Gersdorff G, Liang D, Dominguez-Rosales A, Beg AA, Rojkind M, Bottinger EP. A mechanism of suppression of TGF-beta/SMAD signaling by NF-kappa B/RelA. Genes Dev. 2000; 14:187–197.
256. Gaber T, Dziurla R, Tripmacher R, Burmester GR, Buttgereit F. Hypoxia inducible factor (HIF) in rheumatology: low O-2! See what HIF can do! Ann Rheum Dis. 2005; 64:971–980.
257. Akira S. The role of IL-18 in innate immunity. Curr Opin Immunol. 2000; 12:59–63.
258. Golab J, Zagozdzon , Stoklosal T, Kaminski R, Kozar K, Jakobisiak M. Direct stimulation of macrophages by IL-12 and IL-18–a bridge too far? Immunology Letters. 2000; 72:153–157.
259. Otani T, Nakamura S, Toki M, Motoda R, Kurimoto M, Orita K. Identification of IFN-gamma-producing cells in IL-12/IL-18-treated mice. Cell Immunol. 1999; 198:111–119.
260. Cope A, Le Friec G, Cardone J, Kemper C. The Th1 life cycle: molecular control of IFN-gamma to IL-10 switching. Trends Immunol. 2011; 32:278–286.
261. Starsichova A, Lincova E, Pernicova Z, Kozubik A, Soucek K. TGF-beta1 suppresses IL-6-induced STAT3 activation through regulation of Jak2 expression in prostate epithelial cells. Cell Signal. 2010; 22:1734–1744.
262. Walia B, Wang L, Merlin D, Sitaraman SV. TGF-beta down-regulates IL-6 signaling in intestinal epithelial cells: critical role of SMAD-2. FASEB J. 2003; 17:2130–2132.
263. Kisseleva T, Bhattacharya S, Braunstein J, Schindler CW. Signaling through the JAK/STAT pathway, recent advances and future challenges. Gene. 2002; 285(1-2):1–24.
264. Henson ES, Gibson SB. Surviving cell death through epidermal growth factor (EGF) signal transduction pathways: implications for cancer therapy. Cell Signal. 2006; 18:2089–2097.
265. Watson SA, Grabowska AM, El-Zaatari M, Takhar A. Gastrin - active participant or bystander in gastric carcinogenesis? Nat Rev Cancer. 2006; 6:936–946.
266. Koh TJ, Bulitta CJ, Fleming JV, Dockray GJ, Varro A, Wang TC. Gastrin is a target of the beta-catenin/TCF-4 growth-signaling pathway in a model of intestinal polyposis. J Clin Invest. 2000; 106:533–539.
267. Foshay KM, Gallicano GI. Regulation of Sox2 by STAT3 initiates commitment to the neural precursor cell fate. Stem Cells Dev. 2008; 17:269–278.
268. Asonuma S, Imatani A, Asano N, Oikawa T, Konishi H, Iijima K, Koike T, Ohara S, Shimosegawa T. Helicobacter pylori induces gastric mucosal intestinal metaplasia through the inhibition of interleukin-4-mediated HMG box protein Sox2 expression. Am J Physiol Gastrointest Liver Physiol. 2009; 297:G312–322.
269. Takanaga H, Tsuchida-Straeten N, Nishide K, Watanabe A, Aburatani H, Kondo T. Gli2 is a novel regulator of sox2 expression in telencephalic neuroepithelial cells. Stem Cells. 2009; 27:165–174.
270. Boyer LA, Lee TI, Cole MF, Johnstone SE, Levine SS, Zucker JP, Guenther MG, Kumar RM, Murray HL, Jenner RG, Gifford DK, Melton DA, Jaenisch R, Young RA. Core transcriptional regulatory circuitry in human embryonic stem cells. Cell. 2005; 122:947–956.
271. Coskun M, Troelsen JT, Nielsen OH. The role of CDX2 in intestinal homeostasis and inflammation. Biochim Biophys Acta. 2011; 1812:283–289.
272. Benahmed F, Gross I, Gaunt SJ, Beck F, Jehan F, Domon-Dell C, Martin E, Kedinger M, Freund JN, Duluc I. Multiple regulatory regions control the complex expression pattern of the mouse Cdx2 homeobox gene. Gastroenterology. 2008; 135:1238–1247. 1247 e1231–1233.
273. Raghoebir L, Bakker ER, Mills JC, Swagemakers S, Buscop-van Kempen M, Boerema-de Munck A, Driegen S, Meijer D, Grosveld F, Tibboel D, Smits R, Rottier RJ. SOX2 redirects the developmental fate of the intestinal epithelium toward a premature gastric phenotype. J Mol Cell Biol. 2012.
274. Favaro R, Valotta M, Ferri AL, Latorre E, Mariani J, Giachino C, Lancini C, Tosetti V, Ottolenghi S, Taylor V, Nicolis SK. Hippocampal development and neural stem cell maintenance require Sox2-dependent regulation of Shh. Nat Neurosci. 2009; 12:1248–1256.
275. Mutoh H, Hayakawa H, Sashikawa M, Sakamoto H, Sugano K. Direct repression of Sonic Hedgehog expression in the stomach by Cdx2 leads to intestinal transformation. Biochem J. 2010; 427:423–434.
276. Arici DS, Tuncer E, Ozer H, Simek G, Koyuncu A. Expression of retinoblastoma and cyclin D1 in gastric carcinoma. Neoplasma. 2009; 56:63–67.
277. Takano Y, Kato Y, van Diest PJ, Masuda M, Mitomi H, Okayasu I. Cyclin D2 overexpression and lack of p27

correlate positively and cyclin E inversely with a poor prognosis in gastric cancer cases. The American journal of pathology. 2000; 156:585–594.

278. Sosa MS, Bragado P, Aguirre-Ghiso JA. Mechanisms of disseminated cancer cell dormancy: an awakening field. Nat Rev Cancer. 2014; 14:611–622.

279. Hara T, Ooi A, Kobayashi M, Mai M, Yanagihara K, Nakanishi I. Amplification of c-myc, K-sam, and c-met in gastric cancers: detection by fluorescence in situ hybridization. Lab Invest. 1998; 78:1143–1153.

280. Shibuya M, Yokota J, Ueyama Y. Amplification and expression of a cellular oncogene (c-myc) in human gastric adenocarcinoma cells. Mol Cell Biol. 1985; 5:414–418.

281. Xie HL, Su Q, He XS, Liang XQ, Zhou JG, Song Y, Li YQ. Expression of p21(WAF1) and p53 and polymorphism of p21(WAF1) gene in gastric carcinoma. World J Gastroenterol. 2004; 10:1125–1131.

282. Sugai T, Tsukahara M, Endoh M, Shioi Y, Takebe N, Mue Y, Matsushita H, Toyota M, Suzuki K. Analysis of cell cycle-related proteins in gastric intramucosal differentiated-type cancers based on mucin phenotypes: a novel hypothesis of early gastric carcinogenesis based on mucin phenotype. BMC Gastroenterol. 2010; 10:55.

283. Anagnostopoulos GK, Stefanou D, Arkoumani E, Karagiannis J, Paraskeva K, Chalkley L, Habilomati E, Tsianos E, Agnantis NJ. Immunohistochemical expression of cell-cycle proteins in gastric precancerous lesions. J Gastroenterol Hepatol. 2008; 23:626–631.

284. Fenoglio-Preiser CM, Wang J, Stemmermann GN, Noffsinger A. TP53 and gastric carcinoma: a review. Hum Mutat. 2003; 21:258–270.

285. Mingchao , Devereux TR, Stockton P, Sun K, Sills RC, Clayton N, Portier M, Flake G. Loss of E-cadherin expression in gastric intestinal metaplasia and later stage p altered expression in gastric carcinogenesis. Experimental and toxicologic pathology : official journal of the Gesellschaft fur Toxikologische Pathologie. 2001; 53:237–246.

286. Sun Y, Chen XY, Liu J, Cheng XX, Wang XW, Kong QY, Li H. Differential caspase-3 expression in noncancerous, premalignant and cancer tissues of stomach and its clinical implication. Cancer Detect Prev. 2006; 30:168–173.

287. Kania J, Konturek SJ, Marlicz K, Hahn EG, Konturek PC. Expression of survivin and caspase-3 in gastric cancer. Dig Dis Sci. 2003; 48:266–271.

288. Gomes TS, Oshima CT, Segreto HR, Barrazueta LM, Costa HO, Lima FO, Forones NM, Ribeiro DA. The extrinsic apoptotic signaling pathway in gastric adenocarcinomas assessed by tissue microarray. Pathology, research and practice. 2011; 207:613–617.

289. Yoo NJ, Kim HS, Kim SY, Park WS, Kim SH, Lee JY, Lee SH. Stomach cancer highly expresses both initiator and effector caspases; an immunohistochemical study. APMIS: acta pathologica, microbiologica, et immunologica Scandinavica. 2002; 110:825–832.

290. Yamachika T, Inada K, Fujimitsu Y, Nakamura S, Yamamura Y, Kitou T, Itzkowitz SH, Werther JL, Miki K, Tatematsu M. Intestinalization of gastric signet ring cell carcinomas with progression. Virchows Archiv: an international journal of pathology. 1997; 431:103–110.

291. Shibata T, Noguchi T, Takeno S, Gabbert HE, Ramp U, Kawahara K. Disturbed XIAP and XAF1 expression balance is an independent prognostic factor in gastric adenocarcinomas. Annals of surgical oncology. 2008; 15:3579–3587.

292. Kim SY, Ferrell JE Jr. Substrate competition as a source of ultrasensitivity in the inactivation of Wee1. Cell. 2007; 128:1133–1145.

293. Yasuda R, Harvey CD, Zhong H, Sobczyk A, van Aelst L, Svoboda K. Supersensitive Ras activation in dendrites and spines revealed by two-photon fluorescence lifetime imaging. Nat Neurosci. 2006; 9:283–291.

294. Nash P, Tang X, Orlicky S, Chen Q, Gertler FB, Mendenhall MD, Sicheri F, Pawson T, Tyers M. Multisite phosphorylation of a CDK inhibitor sets a threshold for the onset of DNA replication. Nature. 2001; 414:514–521.

295. Zhang Q, Bhattacharya S, Andersen ME. Ultrasensitive response motifs: basic amplifiers in molecular signalling networks. Open biology. 2013; 3:130031.

296. Ohuchi N, Hand PH, Merlo G, Fujita J, Mariani-Costantini R, Thor A, Nose M, Callahan R, Schlom J. Enhanced expression of c-Ha-ras p21 in human stomach adenocarcinomas defined by immunoassays using monoclonal antibodies and in situ hybridization. Cancer Res. 1987; 47:1413–1420.

297. Czerniak B, Herz F, Gorczyca W, Koss LG. Expression of ras oncogene p21 protein in early gastric carcinoma and adjacent gastric epithelia. Cancer. 1989; 64:1467–1473.

298. Baek MK, Kim MH, Jang HJ, Park JS, Chung IJ, Shin BA, Ahn BW, Jung YD. EGF stimulates uPAR expression and cell invasiveness through ERK, AP-1, and NF-kappaB signaling in human gastric carcinoma cells. Oncol Rep. 2008; 20:1569–1575.

299. Liang B, Wang S, Zhu XG, Yu YX, Cui ZR, Yu YZ. Increased expression of mitogen-activated protein kinase and its upstream regulating signal in human gastric cancer. World journal of gastroenterology: WJG. 2005; 11:623–628.

300. Liu CJ, Shi YQ, Han ZY, Xue Y, Pan YL, Shen HQ, Du JP, Han S, Qiao TD, Fan DM. Zhonghua Yi Xue Za Zhi. 2004; 84:306–311.

301. Yu HG, Ai YW, Yu LL, Zhou XD, Liu J, Li JH, Xu XM, Liu S, Chen J, Liu F, Qi YL, Deng Q, Cao J, Liu SQ, Luo HS, Yu JP. Phosphoinositide 3-kinase/Akt pathway plays an important role in chemoresistance of gastric

cancer cells against etoposide and doxorubicin induced cell death. Int J Cancer. 2008; 122:433–443.

302. Carneiro F, Sobrinho-Simoes M. The prognostic significance of amplification and overexpression of c-met and c-erb B-2 in human gastric carcinomas. Cancer. 2000; 88:238–240.

303. Mizutani T, Onda M, Tokunaga A, Yamanaka N, Sugisaki Y. Relationship of C-erbB-2 protein expression and gene amplification to invasion and metastasis in human gastric cancer. Cancer. 1993; 72:2083–2088.

304. Feoktistova M, Geserick P, Kellert B, Dimitrova DP, Langlais C, Hupe M, Cain K, MacFarlane M, Hacker G, Leverkus M. cIAPs block Ripoptosome formation, a RIP1/caspase-8 containing intracellular cell death complex differentially regulated by cFLIP isoforms. Mol Cell. 2011; 43:449–463.

305. Oberst A, Dillon CP, Weinlich R, McCormick LL, Fitzgerald P, Pop C, Hakem R, Salvesen GS, Green DR. Catalytic activity of the caspase-8-FLIP(L) complex inhibits RIPK3-dependent necrosis. Nature. 2011; 471:363–367.

306. Vanlangenakker N, Vanden Berghe T, Vandenabeele P. Many stimuli pull the necrotic trigger, an overview. Cell Death Differ. 2012; 19:75–86.

307. Wertz IE, O'Rourke KM, Zhou H, Eby M, Aravind L, Seshagiri S, Wu P, Wiesmann C, Baker R, Boone DL, Ma A, Koonin EV, Dixit VM. De-ubiquitination and ubiquitin ligase domains of A20 downregulate NF-kappaB signalling. Nature. 2004; 430:694–699.

308. Eldar A, Dorfman R, Weiss D, Ashe H, Shilo BZ, Barkai N. Robustness of the BMP morphogen gradient in Drosophila embryonic patterning. Nature. 2002; 419:304–308.

309. Rajnakova A, Moochhala S, Goh PM, Ngoi S. Expression of nitric oxide synthase, cyclooxygenase, and p53 in different stages of human gastric cancer. Cancer Lett. 2001; 172:177–185.

310. van Rees BP, Saukkonen K, Ristimaki A, Polkowski W, Tytgat GN, Drillenburg P, Offerhaus GJ. Cyclooxygenase-2 expression during carcinogenesis in the human stomach. J Pathol. 2002; 196:171–179.

311. Chaffer CL, Brueckmann I, Scheel C, Kaestli AJ, Wiggins PA, Rodrigues LO, Brooks M, Reinhardt F, Su Y, Polyak K, Arendt LM, Kuperwasser C, Bierie B, Weinberg RA. Normal and neoplastic nonstem cells can spontaneously convert to a stem-like state. Proc Natl Acad Sci U S A. 2011; 108:7950–7955.

312. Roesch A, Fukunaga-Kalabis M, Schmidt EC, Zabierowski SE, Brafford PA, Vultur A, Basu D, Gimotty P, Vogt T, Herlyn M. A temporarily distinct subpopulation of slow-cycling melanoma cells is required for continuous tumor growth. Cell. 2010; 141:583–594.

313. Parker M, Mohankumar KM, Punchihewa C, Weinlich R, Dalton JD, Li Y, Lee R, Tatevossian RG, Phoenix TN, Thiruvenkatam R, White E, Tang B, Orisme W, Gupta K, Rusch M, Chen X, et al. C11orf95-RELA fusions drive oncogenic NF-kappaB signalling in ependymoma. Nature. 2014; 506:451–455.

314. Chun YS, Lindor NM, Smyrk TC, Petersen BT, Burgart LJ, Guilford PJ, Donohue JH. Germline E-cadherin gene mutations: is prophylactic total gastrectomy indicated? Cancer. 2001; 92:181–187.

315. Schwarz A. Preventive gastrectomy in patients with gastric cancer risk due to genetic alterations of the E-cadherin gene defect. Langenbecks Arch Surg. 2003; 388:27–32.

316. Fitzgerald RC, Caldas C. E-cadherin mutations and hereditary gastric cancer: prevention by resection?. Dig Dis. 2002; 20:23–31.

317. Nojima M, Suzuki H, Toyota M, Watanabe Y, Maruyama R, Sasaki S, Sasaki Y, Mita H, Nishikawa N, Yamaguchi K, Hirata K, Itoh F, Tokino T, Mori M, Imai K, Shinomura Y. Frequent epigenetic inactivation of SFRP genes and constitutive activation of Wnt signaling in gastric cancer. Oncogene. 2007; 26:4699–4713.

318. Park WS, Oh RR, Park JY, Lee SH, Shin MS, Kim YS, Kim SY, Lee HK, Kim PJ, Oh ST, Yoo NJ, Lee JY. Frequent somatic mutations of the beta-catenin gene in intestinal-type gastric cancer. Cancer Res. 1999; 59:4257–4260.

319. Horii A, Nakatsuru S, Miyoshi Y, Ichii S, Nagase H, Kato Y, Yanagisawa A, Nakamura Y. The APC gene, responsible for familial adenomatous polyposis, is mutated in human gastric cancer. Cancer Res. 1992; 52:3231–3233.

320. Lee JH, Abraham SC, Kim HS, Nam JH, Choi C, Lee MC, Park CS, Juhng SW, Rashid A, Hamilton SR, Wu TT. Inverse relationship between APC gene mutation in gastric adenomas and development of adenocarcinoma. Am J Pathol. 2002; 161:611–618.

321. Zheng HC, Xu XY, Xia P, Yu M, Takahashi H, Takano Y. Involvement of inactive GSK3beta overexpression in tumorigenesis and progression of gastric carcinomas. Hum Pathol. 2010; 41:1255–1264.

322. Ren J, Xu S, Guo D, Zhang J, Liu S. Increased expression of alpha5beta1-integrin is a prognostic marker for patients with gastric cancer. Clin Transl Oncol. 2013.

323. Varis A, Wolf M, Monni O, Vakkari ML, Kokkola A, Moskaluk C, Frierson H Jr., Powell SM, Knuutila S, Kallioniemi A, El-Rifai W. Targets of gene amplification and overexpression at 17q in gastric cancer. Cancer Res. 2002; 62:2625–2629.

324. Ang KL, Shi DL, Keong WW, Epstein RJ. Upregulated Akt signaling adjacent to gastric cancers: implications for screening and chemoprevention. Cancer Lett. 2005; 225:53–59.

325. Takahashi R, Tanaka S, Hiyama T, Ito M, Kitadai Y, Sumii M, Haruma K, Chayama K. Hypoxia-inducible factor-1alpha expression and angiogenesis in gastrointestinal stromal tumor of the stomach. Oncol Rep. 2003; 10:797–802.

326. Mizokami K, Kakeji Y, Oda S, Irie K, Yonemura T, Konishi F, Maehara Y. Clinicopathologic significance of hypoxia-inducible factor 1alpha overexpression in gastric carcinomas. J Surg Oncol. 2006; 94:149–154.
327. Urano N, Fujiwara Y, Doki Y, Tsujie M, Yamamoto H, Miyata H, Takiguchi S, Yasuda T, Yano M, Monden M. Overexpression of hypoxia-inducible factor-1 alpha in gastric adenocarcinoma. Gastric Cancer. 2006; 9:44–49.
328. Griffiths EA, Pritchard SA, McGrath SM, Valentine HR, Price PM, Welch IM, West CM. Hypoxia-associated markers in gastric carcinogenesis and HIF-2alpha in gastric and gastro-oesophageal cancer prognosis. Br J Cancer. 2008; 98:965–973.
329. Wu CS, Chen MF, Lee IL, Tung SY. Predictive role of nuclear factor-kappaB activity in gastric cancer: a promising adjuvant approach with caffeic acid phenethyl ester. Journal of clinical gastroenterology. 2007; 41:894–900.
330. Sasaki N, Morisaki T, Hashizume K, Yao T, Tsuneyoshi M, Noshiro H, Nakamura K, Yamanaka T, Uchiyama A, Tanaka M, Katano M. Nuclear factor-kappaB p65 (RelA) transcription factor is constitutively activated in human gastric carcinoma tissue. Clin Cancer Res. 2001; 7:4136–4142.
331. Forones NM, Mandowsky SV, Lourenco LG. Serum levels of interleukin-2 and tumor necrosis factor-alpha correlate to tumor progression in gastric cancer. Hepatogastroenterology. 2001; 48:1199–1201.
332. Izutani R, Katoh M, Asano S, Ohyanagi H, Hirose K. Enhanced expression of manganese superoxide dismutase mRNA and increased TNFalpha mRNA expression by gastric mucosa in gastric cancer. World journal of surgery. 1996; 20:228–233.
333. Sugai H, Kono K, Takahashi A, Ichihara F, Kawaida H, Fujii H, Matsumoto Y. Characteristic alteration of monocytes with increased intracellular IL-10 and IL-12 in patients with advanced-stage gastric cancer. J Surg Res. 2004; 116:277–287.
334. Morisaki T, Katano M, Ikubo A, Anan K, Nakamura M, Nakamura K, Sato H, Tanaka M, Torisu M. Immunosuppressive cytokines (IL-10, TGF-beta) genes expression in human gastric carcinoma tissues. J Surg Oncol. 1996; 63:234–239.
335. Sakamoto T, Saito H, Tatebe S, Tsujitani S, Ozaki M, Ito H, Ikeguchi M. Interleukin-10 expression significantly correlates with minor CD8+ T-cell infiltration and high microvessel density in patients with gastric cancer. Int J Cancer. 2006; 118:1909–1914.
336. Yu J, Zeng Z, Wang S, Tian L, Wu J, Xue L, Lee CW, Zhang M, Goggins WB, Chen M, Hu P, Sung JJ. IL-1B-511 polymorphism is associated with increased risk of certain subtypes of gastric cancer in Chinese: a case-control study. Am J Gastroenterol. 2010; 105:557–564.
337. El-Omar EM, Carrington M, Chow WH, McColl KE, Bream JH, Young HA, Herrera J, Lissowska J, Yuan CC, Rothman N, Lanyon G, Martin M, Fraumeni JF Jr., Rabkin CS. Interleukin-1 polymorphisms associated with increased risk of gastric cancer. Nature. 2000; 404:398–402.
338. Uefuji K, Ichikura T, Mochizuki H. Increased expression of interleukin-1alpha and cyclooxygenase-2 in human gastric cancer: a possible role in tumor progression. Anticancer research. 2005; 25:3225–3230.
339. Crabtree JE, Wyatt JI, Trejdosiewicz LK, Peichl P, Nichols PH, Ramsay N, Primrose JN, Lindley IJ. Interleukin-8 expression in Helicobacter pylori infected, normal, and neoplastic gastroduodenal mucosa. J Clin Pathol. 1994; 47:61–66.
340. Kitadai Y, Haruma K, Sumii K, Yamamoto S, Ue T, Yokozaki H, Yasui W, Ohmoto Y, Kajiyama G, Fidler IJ, Tahara E. Expression of interleukin-8 correlates with vascularity in human gastric carcinomas. The American journal of pathology. 1998; 152:93–100.
341. Nagashima H, Mori M, Sadanaga N, Mashino K, Yoshikawa Y, Sugimachi K. Expression of Fas ligand in gastric carcinoma relates to lymph node metastasis. Int J Oncol. 2001; 18:1157–1162.
342. Osaki M, Kase S, Kodani I, Watanabe M, Adachi H, Ito H. Expression of Fas and Fas ligand in human gastric adenomas and intestinal-type carcinomas: correlation with proliferation and apoptosis. Gastric Cancer. 2001; 4:198–205.
343. Maehara Y, Kakeji Y, Kabashima A, Emi Y, Watanabe A, Akazawa K, Baba H, Kohnoe S, Sugimachi K. Role of transforming growth factor-beta 1 in invasion and metastasis in gastric carcinoma. J Clin Oncol. 1999; 17:607–614.
344. Vagenas K, Spyropoulos C, Gavala V, Tsamandas AC. TGFbeta1, TGFbeta2, and TGFbeta3 protein expression in gastric carcinomas: correlation with prognostics factors and patient survival. J Surg Res. 2007; 139:182–188.
345. Woo S, Lee BL, Yoon J, Cho SJ, Baik TK, Chang MS, Lee HE, Park JW, Kim YH, Kim WH. Constitutive activation of signal transducers and activators of transcription 3 correlates with better prognosis, cell proliferation and hypoxia-inducible factor-1alpha in human gastric cancer. Pathobiology: journal of immunopathology, molecular and cellular biology. 2011; 78:295–301.
346. McKenzie RC, Jones CL, Tosi I, Caesar JA, Whittaker SJ, Mitchell TJ. Constitutive activation of STAT3 in Sezary syndrome is independent of SHP-1. Leukemia. 2012; 26:323–331.
347. Mizoshita T, Tsukamoto T, Nakanishi H, Inada K, Ogasawara N, Joh T, Itoh M, Yamamura Y, Tatematsu M. Expression of Cdx2 and the phenotype of advanced gastric cancers: relationship with prognosis. J Cancer Res Clin Oncol. 2003; 129:727–734.
348. Fukaya M, Isohata N, Ohta H, Aoyagi K, Ochiya T, Saeki N, Yanagihara K, Nakanishi Y, Taniguchi H, Sakamoto H, Shimoda T, Nimura Y, Yoshida T, Sasaki H. Hedgehog signal activation in gastric pit cell and in diffuse-type gastric cancer. Gastroenterology. 2006; 131:14–29.

SUPPLEMENTARY TABLES

Supplementary Table S1: Reference to each interaction in Figure 1

Components	Activation	References	Inhibition	References
Rb			Cyclin D/Cdk4,6*	[2]
			Cyclin E/Cdk2*	[2]
			Caspase 3§	[113]
Cyclin D/Cdk4,6	c-Myc‡	[4]	p21⁎	[13]
	β-catenin‡	[5–7]	GSK3β*	[114]
	NF-κB‡	[8, 9]	SOX2‡	[24]
	ERKǀ	[10]		
	JNK/p38ǀ	[11]		
	SHH‡	[12]		
Cyclin E/Cdk2	c-Myc‡	[4]	p21†	[14, 18]
	E2F‡	[3]	p27†	[3, 14]
c-Myc	E2F‡	[115]	p53‡	[22, 23]
	Aktǀ	[104]	TGF-βǀ	[116, 117]
	ERKǀ	[104]	IFN-γǀ	[118–121]
	JNK/p38ǀ	[104]	p21†	[105, 122]
	β-catenin‡	[123]		
	NF-κB‡	[46]		
	HIF2α‡	[121]		
	SHH‡	[12]		
E2F	E2F‡	[124]	Rb†	[125, 126]
	c-Myc‡	[127]	p21†	[128, 129]
p21	p53‡	[18, 21]	c-Myc‡	[17, 18]
	TGF-β‡	[15, 16]	Akt*	[110]
	E2F‡	[130, 131]		
	HIF‡	[132]		
	IFNγǀ	[133–135]		
	ERK‡ǀ	[105]		
	Cdx2‡	[91]		
p27	PTEN⁎	[136]	c-Myc‡	[19, 20]
	E-cadherin⁎	[137]	Aktǀ	[138]
	IFNγǀ	[135, 139, 140]	Cyclin E/Cdk2*	[141]
	TGF-βǀ	[142]	ERKǀ	[143, 144]
	SOX2ǀ	[24]		
p53	E2F‡	[145, 146]	Aktǀ	[42, 43]
	c-Myc‡	[20, 147, 148]		

(*Continued*)

Components	Activation	References	Inhibition	References
	NF-κB‡	[46]		
	Cytochrome *c* (Endo G, AIF, CAD)‖	[149]		
Caspase 3,7	Caspase 8,10§	[31–35]	p21†	[150, 151]
	Cytochrome *c*§	[31–36]	XIAP†	[44, 45]
Cytochrome *c*	Bad‖	[28]	Akt*	[108, 109]
	Bax‖	[28, 29]	XIAP†	[44, 45]
	Bid‖	[28]		
	E2F‡	[152, 153]		
Caspase 8,10	Fas†	[28]		
	TNF-α†	[28, 37]		
	IL-1†	[154]		
XIAP	Akt*	[155]	Cytochrome *c*†	[44]
	NF-κB‡	[46, 47]		
Bcl-2	VEGF※	[156]	p53‡	[38, 39]
	NF-κB‡	[48]	TGF-β‡‖	[157]
	ERK*	[158]	Bad†	[28]
	SHH‡	[12]	c-Myc‡	[159]
**Bcl-x_L**	EGF/EGFR‡‖	[160–163]	Bad†	[28]
	IGF/IGFR‡‖	[164, 165]	c-Myc‡	[159]
	NF-κB‡	[46, 47]		
Bid	Caspase 8,10§	[28]		
Bad	p53‡	[166]	Akt*	[107]
			ERK*	[167]
Bax/Bak	c-Myc‡	[159]	Bcl-2†	[168]
	p53‡	[38, 39]	Bcl-x_L†	[30]
Ras	HGF*‖	[99]		
	EGF*‖	[98]		
	VEGF*‖	[100]		
	Integrin*‖	[42, 43, 101, 102]		
PI3K-Akt	Ras*	[103]	PTEN*	[112]
	IL-8*‖	[72]		
	E-cadherin	[169]		
	Integrin*‖	[75, 170]		
	HGF*	[171, 172]		
	VEGF*	[100]		
	EGF*	[173]		

(*Continued*)

Components	Activation	References	Inhibition	References
	IGF*	[174–176]		
	TGF-β*	[157, 177]		
PTEN			Akt	[178, 179]
			NF-κB‡	[180]
			β-catenin‡‖	[181]
ERK	Ras*	[103]	PTEN*	[112]
	Gastrin*‖	[182]	MKP*	[183]
	Integrin*‖	[101, 102]		
	STAT3※‖	[184]		
JNK, p38	TGF-β*‖	[185, 186]	PTEN*	[112]
	TNF-α*‖	[187]	MKP*	[188, 189]
	IL-1*‖	[54–56]		
	IL-8*‖	[72]		
	EGF*‖	[173]		
	VEGF*‖	[100]		
	Gastrin*‖	[182]		
MKP	ERK*	[190, 191]		
	JNK	[192]		
VEGF/VEGFR	IL-8*	[72]		
	HIF‡	[71]		
	Akt‖	[76]		
	HGF/Met‖	[74]		
	COX-2‖	[193]		
	IL-1‡‖	[73]		
	STAT3‡	[194]		
	Integrin*	[75]		
EGF/EGFR	IL-8*	[72]		
	Integrin*	[75]		
IGF-1R	HIF*	[195]	p53‖	[40, 41]
HGF/SF c-Met	ERK‡‖	[106]		
	HIF‡	[196]		
	β-catenin‡	[197, 198]		
Integrin-FAK	EGF*	[199–201]	PTEN*	[102]
	HGF*‡	[200, 202]		
E-cadherin			Integrin*	[203]
			IL-1β‖	[204]
			Caspase 3,7§	[205]

(*Continued*)

Components	Activation	References	Inhibition	References
			c-Met*	[206–208]
			Zeb1/2‡	[209, 210]
ZEB1/2	TGF-β‡ǀ	[211, 212]	p53ǀ	[213, 214]
	NF-κB‡	[211, 212]		
β-catenin/LEF/TCF4 (cytoplasm)	Gastrin‡ǀ	[215]	E-cadherin†	[216]
			SOX2†	[217]
	HGF/c-Met*	[218]	GSK3β*	[219, 220]
	EGF*	[221]		
	IGF-1*	[222, 223]		
HIF	Akt*	[76, 224]	p53*	[225]
	NF-κB*	[226, 227]		
	c-Myc*			
NF-κB	JNK, p38ǀ	[228]	iκB†	[46, 229]
			STAT3‡	[230, 231]
iκB	NF-κB‡	[46, 232, 233]	TNF-αǀ	[229]
	ERK‡ǀ	[65]	IL-1βǀ	[65, 229, 234]
	JNK/p38‡ǀ	[65]	Akt*	[235–238]
			Fasǀ	[239]
			TGF-βǀ	[240]
TNF-α	NF-κB‡	[46]	IL-10ǀ	[241]
	ERK‡ǀ	[242]		
	JNK/p38‡ǀ	[242]		
IL-10	TNF-αǀ	[243]	IFN-γǀ	[244]
	Fas※ǀ	[245, 246]		
IL-1	NF-κB‡		IL-1ǀ	[65, 247–250]
	JNK/p38‡ǀ	[65]		
IL-8	NF-κB‡	[46, 57, 65, 66]		
	JNK/p38‡ǀ	[57–61]		
Fas	p53‡	[38, 39]	Rasǀ	[251]
	NF-κB‡	[46]		
	IFN-γ‡ǀ	[252]		
COX-2	NF-κB‡	[46, 68, 69]		
	JNK/p38‡	[62–64]		
TGF-β	Integrin†	[253, 254]	NF-κB‡ǀ	[240, 255]
	HIF‡	[256]		
IFN-γ	IL-1ǀ	[257–259]	IL-10ǀ	[260]

(*Continued*)

Components	Activation	References	Inhibition	References
STAT3	IL-10*	[184]	TGF-β*	[261, 262]
	Gastrin*	[182]		
	EGF*	[263, 264]		
	HGF*	[263]		
GSK3β			Akt*	[111]
			NF-κBǀ	[238]
Gastrin	β-catenin‡	[265, 266]		
SOX2	STAT3‡	[267]	IFN-γǀ	[268]
	SHH‡	[269]		
	SOX2‡	[270]		
CDX2	β-catenin‡	[271]	SOX2‡	[272, 273]
	CDX2‡	[271]		
SHH	SOX2‡	[274]	CDX2‡	[275]
			GSK3β*	[12]

* The molecular mechanism of inhibition and activation interactions in the network.
* Components Phosphorylate or dephoephorylate targets, which result in their activation or inactivation;
§ Components cleave tagets, which result in their activation or inactivation;
‡ Components transcript target genes, and induce expression of targets;
† Components bind to targets, which inhibit or activate the activity of targets, or sequester them in the cytoplasm or nuclear;
ǀ Components activate or inhibit targets indirectly, intermediates were abbreviated;
※ Components activate or inhibit targets through mechanisms still unknown.

Supplementary Table S2: Comparing model results and low throughput experimental data of the relative change of components

Components	Theoretical Results		Experimental Data	Experiment types
	P_1/A_1	P_2/A_1	GC/N	
Rb	↓	↓	↓[276]	RT-PCR
Cyclin D/Cdk4,6	↑	↑	↑[276, 277]	RT-PCR, IHC, WB
Cyclin E/Cdk2	↑	↑	↑[277]	RT-PCR, IHC, WB
E2F	↑	↑	↑[278]	RT-PCR, IHC, WB
Myc	↑	↑	↑[279, 280]	FISH, SB
p21	↑	↑	↓[281, 282]	IHC, PCR
p27	↓	↓	↓[277, 283]	RT-PCR, IHC, WB
p53	↑	↑	–[284, 285]	IHC
Caspase 3,7	↑	↑	↓[286, 287]	RT-PCR, IHC, WB
			↑[288, 289]	

(*Continued*)

Components	Theoretical Results		Experimental Data	Experiment types
	P_1/A_1	P_2/A_1	GC/N	
Cytochrome *c*	↑	↑	↓[290]	IHC
			↑[289]	
Caspase 8,10	↑	↑	↓[288]	IHC
			↑[289]	
XIAP	↑	↑	↑[291]	IHC
Bcl-2	↑	↑	↑[292]	IHC
Bcl-X_L	↑	↑	↑[293]	IHC
			↓[294]	IHC
Bid	↑	↑	N/A	
Bad	↑	↑	↑[295]	IHC
Bax	↑	↑	↑[294]	IHC
Ras	↑	↑	↑[296, 297]	IHC,
ERK	↑	↑	↑[298, 299]	WB, IHC
JNK, p38	↑	↑	↑[298, 299]	WB, IHC
MKP1/DUSP1	↑	↑	↑[300]	WB, RT-PCR
PTEN	↓	↓	↓[301]	RT-PCR
EGF/EGFR	↑	↑	↑[302–304]	IHC, SB
IGF/IGFR	↑	↑	↑[305, 306]	IHC
HGF/ c-Met	↑	↑	↑[279, 302]	IHC, SB, FISH
VEGF/VEGFR	↑	↑	↑[307, 308]	IHC
COX-2	↑	↑	↑[309–312]	IHC, WB
E-cadherin	↓	↓	↓[285, 313–316]	IHC, RT-PCR
ZEB1/2	↑	↑	↑[313]	RT-qPCR
β-catenin	↑	↑	↑[90, 317]	RT-PCR, IHC, WB
			↑[90, 318–320]	
GSK3β	↓	↓	↓[321]	IHC, WB
Integrin-FAK	↑	↑	↑[322, 323]	IHC, qRT-PCR, cDNA Microarrays
PI3K-Akt	↑	↑	↑[324]	RT-PCR, immunoblotting
HIF	↑	↑	↑[325–328]	IHC
NF-κB	↑	↑	↑[329, 330]	IHC
IκB	↑	↑	N/A	
TNF-α	↑	↑	↑[331, 332]	qRT-PCR
IL-10	↑	↑	↑[333–335]	RT-PCR
IL-1	↑	↑	↑[336, 337] [338]	RT-PCR
IL-8	↑	↑	↑[339, 340]	RT-PCR

(*Continued*)

Components	Theoretical Results		Experimental Data	Experiment types
	P_1/A_1	P_2/A_1	GC/N	
IFN-γ	↑	↑	N/A	
Fas	↑	↑	↑[341, 342]	WB, IHC
TGF-β	↑	↑	↑[334, 343, 344]	IHC
STAT3	↑	↑	↑[345, 346]	IHC
Gastrin	↑	↑	↑[85, 86]	IHC, RT-PCR
Sox2	↓	↓	↓[24]	qRT-PCR, WB
Cdx2	↑	↑	↑[347]	IHC
Shh	↑	↓	↑[348]	qRT-PCR, IHC
Consistency **(Theoretical/ Experimental)**	**97.5%(39/40)**	**95%(38/40)**		

If the ratios (for example, P_1 state to A_1 state) is greater than 1, we define the activity of the component is increased, otherwise is decreased. If the experimental results are conflict in independent studies, we define uncertain variation in component activity or expression of components between gastric cancer tissues and normal gastric epithelium. GC, gastric cancer;
N, normal gastric epithelium;
↑, means increased activity or expression of component;
↓, means decreased activity or expression of component;
__, non-significant or uncertain variation in component activity or expression;
N/A, means experimental results were not found. RT-PCR, reverse transcriptional polymerase chain reaction;
qRT-PCR, quantitative real-time polymerase chain reaction;
IHC, immunohistochemistry;
WB, western blot;
SB, southern blot;
FISH, fluorescence in situ hybridization;

Supplementary Table S3: Comparing model results and high-throughput experimental data of the relative change of components

	Theoretical Results		GSE19826	GSE22183	Gene Symbol
	P_1/A_1	P_2/A_1	GC/N	GC/N	
Rb	↓	↓	-	↓	RB1
Cyclin D/Cdk4,6	↑	↑	↑	↑	CCND1–3, CDK4, CDK6
Cyclin E/Cdk2	↑	↑	↑	↑	CCNE1–2, CDK2
Myc	↑	↑	↑	↑	MYC
E2F	↑	↑	↑	↑	E2F1, E2F2, E2F3
p21	↑	↑	-	↓	CDKN1A
p27	↓	↓	-	↑	CDKN1B
p53	↑	↑	-	↑	TP53

(Continued)

	Theoretical Results		GSE19826	GSE22183	Gene Symbol
	P_1/A_1	P_2/A_1	GC/N	GC/N	
Caspase 3,7	↑	↑	ND	↑	CASP3, CASP7
Cytochrome *c*	↑	↑	ND	↑	CYCS, CASP9, APAF1, DIABLO
Caspase 8,10	↑	↑	ND	↑	CASP8, CASP10
XIAP	↑	↑	↓	↑	XIAP
Bcl-2	↑	↑	↑	↑	BCL2
Bcl-xL	↑	↑	↑	↑	BCL2L1
Bid	↑	↑	↑	↑	BID
Bad	↑	↑	↓	-	BAD
Bax	↑	↑	↑	↑	BAX
Ras	↑	↑	↑	↑	KRAS, MRAS, RRAS, HRAS
Akt	↑	↑	↑	↑	AKT1, AKT2, AKT3
PTEN	↓	↓	-	↓	PTEN
ERK	↑	↑	↓	↑	MAPK1, MAPK3, MAPK6
JNK/p38	↑	↑	↑	↑	MAPK7–14
MKP	↑	↑	↑	↓	DUSP1
VEGF/VEGFR	↑	↑	-	↓	VEGFA
EGF/EGFR	↑	↑	↑	↑	EGF, EGFR, ERBB2
IGF/IGFR	↑	↑	↑	↓	IGF1, IGF1R, IGF2R
HGF/c-Met	↑	↑	↑	↑	HGF, MET
Integrin/FAK	↑	↑	↑	↑	ITGA2
E-cadherin	↓	↓	-	↑	CDH1
Zeb1/2	↑	↑	↑	↓	ZEB1, ZEB2
β-catenin	↑	↑	↑	↑	CTNNB1
HIF	↑	↑	↑	↓	HIF1A, ARNT, ARNT2, EPAS1
NFκB	↑	↑	↑	↑	NFKB1, NFKB2, RELA, RELB, REL
iκB	↑	↑	↑	↑	NFKBIA, NFKBIB, NFKBIE
TNF-α	↑	↑	↑	↑	TNF, TNFRSF1A, TNFRSF1B
IL10	↑	↑	↑	↑	IL10, IL10RA, IL10RB
IL1	↑	↑	ND	↑	IL1A, IL1B, IL1R1, IL1R2
IL8	↑	↑	↑	↑	IL8, CXCR1, CXCR2
Fas	↑	↑	↑	↑	FAS, FASLG
COX2	↑	↑	↓	↓	COX2
TGF-β	↑	↑	↑	ND	TGFB1, TGFB2, TGFB3, TGFBR1, TGFBR2, TGFBR3

(*Continued*)

	Theoretical Results		GSE19826	GSE22183	Gene Symbol
	P_1/A_1	P_2/A_1	GC/N	GC/N	
IFN-γ	↑	↑	↑	ND	IFNG, IFNGR1, IFNGR2
STAT3	↑	↑	↑	↓	STAT3
GSK3β	↓	↓	-	↓	GSK3B
Gastrin	↑	↑	↓	↑	GAST
Sox2	↓	↓	↓	↑	SOX2
Cdx2	↑	↑	↑	↑	CDX2
Shh	↑	↓	ND	ND	SHH, GLI1, GLI2, GLI3, PTCH1, PTCH2, SMO
GSE19826	**72.1%(40/47)**	**72.1%(40/47)**			
GSE22183	**73.3%(33/46)**	**71.1%(32/46)**			

If the ratios of one component between two stable states (for example, P1 state to A1 state) are greater than 1, we define the component activity as increased;
otherwise, the component activity is decreased.
GC, gastric cancer;
N, normal gastric epithelium;
↑, increased component activity or concentration;
↓, decreased component activity or concentration;
__, non-significant or uncertain variation in component activity or expression;
ND, non-determined variation in component expression.

Supplementary Table S4: Attractors of working endogenous network when n = 3, a = 7

Components	A_1	A_2	A_3	R_1	R_2	P_2	D
Rb	1.0000	1.0000	1.0000	0.9409	0.2155	0.1395	0.9586
Cyclin D/Cdk4,6	0.0006	0.0000	0.0000	0.1587	0.8000	0.8041	0.0010
Cyclin E/Cdk2	0.0000	0.0000	0.0000	0.1641	0.1963	0.7116	0.0000
Myc	0.0156	0.0098	0.0026	0.3064	0.3085	0.3072	0.0030
E2F	0.0000	0.0000	0.0000	0.0245	0.1843	0.6998	0.0000
p21	0.0000	0.0000	0.7298	0.1207	0.1220	0.1237	0.7872
p27	0.9340	0.9233	0.9233	0.1097	0.0828	0.0639	0.9297
p53	0.0000	0.0000	0.0000	0.0514	0.0524	0.1019	0.8154
Caspase 3,7	0.0000	0.0000	0.0000	0.0825	0.0821	0.0823	0.1844
Cytochrome c	0.0000	0.0000	0.0000	0.0346	0.0354	0.0633	0.8667
Caspase 8,10	0.0000	0.0000	0.0000	0.4939	0.4969	0.4960	0.1459
XIAP	0.0098	0.0098	0.0098	0.8654	0.8703	0.8688	0.0019
Bcl-2	0.0059	0.0000	0.0000	0.2846	0.2733	0.2707	0.0198
Bcl-xL	0.0000	0.0000	0.0000	0.7448	0.7439	0.7443	0.0000
Bid	0.0000	0.0000	0.0000	0.4576	0.4620	0.4606	0.0333
Bad	0.0000	0.0000	0.0000	0.0001	0.0001	0.0009	0.7831
Bax	0.0000	0.0000	0.0000	0.0415	0.0425	0.0432	0.7944
Ras	0.0000	0.0000	0.0000	0.9507	0.9546	0.9546	0.1580
Akt	0.1122	0.1122	0.1122	0.9537	0.9678	0.9678	0.1130
PTEN	0.9902	0.9902	0.9902	0.1337	0.0800	0.0796	0.9892
ERK	0.0000	0.0000	0.0000	0.4331	0.4388	0.4388	0.0495
JNK/p38	0.0000	0.0000	0.0000	0.4416	0.4432	0.4432	0.0203
MKP	0.0000	0.0000	0.0000	0.5395	0.5456	0.5456	0.0069
VEGF/VEGFR	0.0098	0.0098	0.0098	0.9560	0.9595	0.9593	0.0718
EGF/EGFR	0.0000	0.0000	0.0000	0.8483	0.8574	0.8572	0.0000
IGF/IGFR	0.0000	0.0000	0.0000	0.8201	0.8224	0.8143	0.0000
HGF/c-Met	0.0000	0.0000	0.0000	0.8384	0.9093	0.9091	0.0050
Integrin/FAK	0.0000	0.0000	0.0000	0.8789	0.9031	0.9031	0.0000
E-cadherin	1.0000	1.0000	1.0000	0.0790	0.0704	0.0706	0.9586
Zeb1/2	0.0000	0.0000	0.0000	0.7033	0.7035	0.7006	0.0000
β-catenin	0.0000	0.0000	0.0000	0.1746	0.8805	0.8843	0.0000
HIF	0.0098	0.0098	0.0098	0.8683	0.8730	0.8673	0.0022
NFκB	0.0000	0.0000	0.0000	0.3751	0.3776	0.3753	0.0004
iκB	0.0000	0.0000	0.0000	0.0603	0.0592	0.0590	0.0060
TNF-α	0.0000	0.0000	0.0000	0.4483	0.4501	0.4497	0.0066
IL10	0.0000	0.0000	0.0000	0.3694	0.3718	0.3714	0.1459

(*Continued*)

Components	A_1	A_2	A_3	R_1	R_2	P_2	D
IL1	0.0000	0.0000	0.0000	0.3666	0.3681	0.3673	0.0020
IL8	0.0000	0.0000	0.0000	0.4930	0.4965	0.4948	0.0020
Fas	0.0000	0.0000	0.0000	0.0420	0.0422	0.0421	0.4961
COX2	0.0000	0.0000	0.0000	0.4930	0.4965	0.4948	0.0020
TGF-β	0.0000	0.0000	0.0000	0.6595	0.6590	0.6619	0.0000
IFN-γ	0.0000	0.0000	0.0000	0.1895	0.1903	0.1896	0.0000
STAT3	0.0000	0.0000	0.0000	0.2983	0.3107	0.3081	0.0333
GSK3β	0.9902	0.9902	0.9902	0.1344	0.1295	0.1296	0.9892
Gastrin	0.0000	0.0000	0.0000	0.0359	0.8270	0.8288	0.0000
Sox2	0.7389	0.0000	0.0000	0.8485	0.2009	0.1949	0.8048
Cdx2	0.0000	0.0000	0.7370	0.0068	0.8528	0.8581	0.0000
Shh	0.0947	0.0000	0.0000	0.7969	0.0100	0.0091	0.1031

A (A_1, A_2 and A_3), R (R_1 and R_2), P (P_1 and P_2) are point attractors, D is a cyclic attractor. Dynamical trajectories of Caspase 8/10, Bid, Fas, VEGF/VEGFR, IL10, Ras and STAT3 is showed in Supplemnetary Figure S1.

Supplementary Table S5: Attractors of working endogenous network when *n* = 3, a = 8

Components	A_1	A_2	A_3	R_1	R_2	P_2	D
Rb	1.0000	1.0000	1.0000	0.9443	0.1867	0.1063	0.9698
Cyclin D/Cdk4,6	0.0005	0.0000	0.0000	0.1432	0.8126	0.8186	0.0006
Cyclin E/Cdk2	0.0000	0.0000	0.0000	0.1581	0.1961	0.7949	0.0000
Myc	0.0142	0.0082	0.0016	0.2883	0.2903	0.2890	0.0020
E2F	0.0000	0.0000	0.0000	0.0208	0.1872	0.7883	0.0000
p21	0.0000	0.0000	0.8024	0.1073	0.1087	0.1104	0.8219
p27	0.9447	0.9328	0.9328	0.0986	0.0760	0.0534	0.9404
p53	0.0000	0.0000	0.0000	0.0462	0.0477	0.0978	0.8441
Caspase 3,7	0.0000	0.0000	0.0000	0.0785	0.0783	0.0784	0.1562
Cytochrome c	0.0000	0.0000	0.0000	0.0379	0.0382	0.0599	0.8921
Caspase 8,10	0.0000	0.0000	0.0000	0.5073	0.5095	0.5085	0.2012
XIAP	0.0082	0.0082	0.0082	0.8825	0.8858	0.8846	0.0013
Bcl-2	0.0062	0.0000	0.0000	0.2713	0.2616	0.2587	0.0117
Bcl-xL	0.0000	0.0000	0.0000	0.7667	0.7649	0.7656	0.0000
Bid	0.0000	0.0000	0.0000	0.5109	0.5142	0.5127	0.0197
Bad	0.0000	0.0000	0.0000	0.0001	0.0001	0.0008	0.8188
Bax	0.0000	0.0000	0.0000	0.0339	0.0348	0.0353	0.8285
Ras	0.0000	0.0000	0.0000	0.9590	0.9617	0.9617	0.1121
Akt	0.1009	0.1009	0.1009	0.9625	0.9732	0.9732	0.1014
PTEN	0.9918	0.9918	0.9918	0.1167	0.0695	0.0690	0.9911
ERK	0.0000	0.0000	0.0000	0.4134	0.4176	0.4177	0.0353
JNK/p38	0.0000	0.0000	0.0000	0.4198	0.4210	0.4210	0.0128
MKP	0.0000	0.0000	0.0000	0.5364	0.5412	0.5413	0.0049
VEGF/VEGFR	0.0082	0.0082	0.0082	0.9632	0.9656	0.9654	0.0480
EGF/EGFR	0.0000	0.0000	0.0000	0.8726	0.8784	0.8782	0.0000
IGF/IGFR	0.0000	0.0000	0.0000	0.8465	0.8478	0.8396	0.0000
HGF/c-Met	0.0000	0.0000	0.0000	0.8599	0.9218	0.9218	0.0037
Integrin/FAK	0.0000	0.0000	0.0000	0.9009	0.9187	0.9187	0.0000
E-cadherin	1.0000	1.0000	1.0000	0.0651	0.0592	0.0593	0.9698
Zeb1/2	0.0000	0.0000	0.0000	0.7240	0.7236	0.7207	0.0000
β-catenin	0.0000	0.0000	0.0000	0.1561	0.8884	0.8939	0.0000
HIF	0.0082	0.0082	0.0082	0.8848	0.8879	0.8820	0.0015
NFκB	0.0000	0.0000	0.0000	0.3711	0.3730	0.3706	0.0002
iκB	0.0000	0.0000	0.0000	0.0536	0.0529	0.0527	0.0043
TNF-α	0.0000	0.0000	0.0000	0.4328	0.4341	0.4337	0.0048
IL10	0.0000	0.0000	0.0000	0.3714	0.3732	0.3728	0.2012

(*Continued*)

Components	A_1	A_2	A_3	R_1	R_2	P_2	D
IL1	0.0000	0.0000	0.0000	0.3623	0.3635	0.3627	0.0013
IL8	0.0000	0.0000	0.0000	0.5002	0.5030	0.5011	0.0013
Fas	0.0000	0.0000	0.0000	0.0397	0.0398	0.0396	0.5761
COX2	0.0000	0.0000	0.0000	0.5002	0.5030	0.5011	0.0013
TGF-β	0.0000	0.0000	0.0000	0.6525	0.6514	0.6546	0.0000
IFN-γ	0.0000	0.0000	0.0000	0.1955	0.1960	0.1953	0.0000
STAT3	0.0000	0.0000	0.0000	0.2841	0.2941	0.2912	0.0197
GSK3β	0.9918	0.9918	0.9918	0.1171	0.1138	0.1139	0.9911
Gastrin	0.0000	0.0000	0.0000	0.0295	0.8487	0.8510	0.0000
Sox2	0.8095	0.0000	0.0000	0.8549	0.1979	0.1904	0.8326
Cdx2	0.0000	0.0000	0.8090	0.0049	0.8612	0.8684	0.0000
Shh	0.0919	0.0000	0.0000	0.8227	0.0095	0.0084	0.0946

A (A_1, A_2 and A_3), R (R_1 and R_2), P (P_1 and P_2) are point attractors, D is a cyclic attractor. Dynamical trajectories of Caspase 8/10, Bid, Fas, VEGF/VEGFR, IL10, Ras and STAT3 is showed in Supplementary Figure S1.

Supplementary Table S6: Attractors of working endogenous network when $n = 3$, $a = 10$

Components	A_1	A_2	A_3	R_1	R_2	P_1	P_2	D
Rb	1.0000	1.0000	1.0000	0.9473	0.1495	0.1425	0.0763	0.9835
Cyclin D/ Cdk4,6	0.0003	0.0000	0.0000	0.1224	0.8241	0.1221	0.8328	0.0003
Cyclin E/ Cdk2	0.0000	0.0000	0.0000	0.1501	0.2075	0.8432	0.8586	0.0001
Myc	0.0107	0.0056	0.0008	0.2617	0.2635	0.2605	0.2625	0.0010
E2F	0.0000	0.0000	0.0000	0.0160	0.2018	0.8211	0.8556	0.0512
p21	0.0000	0.0000	0.8621	0.0889	0.0894	0.0916	0.0907	0.8684
p27	0.9582	0.9470	0.9470	0.0828	0.0651	0.0535	0.0420	0.9560
p53	0.0000	0.0000	0.0000	0.0392	0.0411	0.0852	0.0840	0.8836
Caspase 3,7	0.0000	0.0000	0.0000	0.0707	0.0704	0.0707	0.0704	0.1189
Cytochrome c	0.0000	0.0000	0.0000	0.0387	0.0385	0.0505	0.0500	0.9278
Caspase 8,10	0.0000	0.0000	0.0000	0.5274	0.5291	0.5264	0.5282	0.4243
XIAP	0.0056	0.0056	0.0056	0.9049	0.9075	0.9042	0.9069	0.0006
Bcl-2	0.0051	0.0000	0.0000	0.2508	0.2434	0.2481	0.2409	0.0332
Bcl-xL	0.0000	0.0000	0.0000	0.7939	0.7918	0.7949	0.7925	0.0000
Bid	0.0000	0.0000	0.0000	0.5947	0.5969	0.5933	0.5957	0.4331
Bad	0.0000	0.0000	0.0000	0.0001	0.0001	0.0006	0.0005	0.8665
Bax	0.0000	0.0000	0.0000	0.0247	0.0254	0.0251	0.0257	0.8731
Ras	0.0000	0.0000	0.0000	0.9692	0.9707	0.9691	0.9707	0.4767
Akt	0.0826	0.0826	0.0826	0.9691	0.9795	0.9690	0.9795	0.0836

(*Continued*)

Components	A_1	A_2	A_3	R_1	R_2	P_1	P_2	D
PTEN	1.0000	1.0000	1.0000	0.1066	0.0594	0.1068	0.0588	1.0000
ERK	0.0000	0.0000	0.0000	0.3803	0.3833	0.3803	0.3833	0.0595
JNK/p38	0.0000	0.0000	0.0000	0.3846	0.3855	0.3846	0.3855	0.0433
MKP	0.0000	0.0000	0.0000	0.5281	0.5318	0.5281	0.5318	0.0029
VEGF/ VEGFR	0.0056	0.0056	0.0056	0.9720	0.9735	0.9719	0.9734	0.4500
EGF/EGFR	0.0000	0.0000	0.0000	0.9021	0.9057	0.9019	0.9056	0.0000
IGF/IGFR	0.0000	0.0000	0.0000	0.8811	0.8819	0.8744	0.8756	0.0000
HGF/c-Met	0.0000	0.0000	0.0000	0.8892	0.9381	0.8876	0.9384	0.0021
Integrin/FAK	0.0000	0.0000	0.0000	0.9237	0.9381	0.9235	0.9382	0.0000
E-cadherin	1.0000	1.0000	1.0000	0.0485	0.0450	0.0487	0.0451	0.9835
Zeb1/2	0.0000	0.0000	0.0000	0.7529	0.7521	0.7505	0.7498	0.0000
β-catenin	0.0000	0.0000	0.0000	0.1315	0.8916	0.1312	0.8995	0.0000
HIF	0.0056	0.0056	0.0056	0.9065	0.9090	0.9013	0.9041	0.0007
NFκB	0.0000	0.0000	0.0000	0.3621	0.3636	0.3601	0.3617	0.0001
iκB	0.0000	0.0000	0.0000	0.0445	0.0439	0.0444	0.0437	0.0017
TNF-α	0.0000	0.0000	0.0000	0.4066	0.4075	0.4063	0.4071	0.0017
IL10	0.0000	0.0000	0.0000	0.3711	0.3723	0.3707	0.3720	0.4243
IL1	0.0000	0.0000	0.0000	0.3539	0.3547	0.3532	0.3541	0.0008
IL8	0.0000	0.0000	0.0000	0.5107	0.5130	0.5088	0.5113	0.0008
Fas	0.0000	0.0000	0.0000	0.0355	0.0356	0.0354	0.0355	0.4193
COX2	0.0000	0.0000	0.0000	0.5107	0.5130	0.5088	0.5113	0.0008
TGF-β	0.0000	0.0000	0.0000	0.6366	0.6350	0.6396	0.6379	0.0000
IFN-γ	0.0000	0.0000	0.0000	0.2032	0.2035	0.2026	0.2030	0.0000
STAT3	0.0000	0.0000	0.0000	0.2618	0.2691	0.2590	0.2665	0.4331
GSK3β	0.9944	0.9944	0.9944	0.0945	0.0919	0.0946	0.0920	0.9942
Gastrin	0.0000	0.0000	0.0000	0.0222	0.8764	0.0221	0.8792	0.0000
Sox2	0.8671	0.0000	0.0000	0.8551	0.1964	0.8558	0.1878	0.8860
Cdx2	0.0000	0.0000	0.8670	0.0031	0.8658	0.0030	0.8753	0.0000
Shh	0.0800	0.0000	0.0000	0.8549	0.0094	0.8552	0.0081	0.0808

A (A_1, A_2 and A_3), R (R_1 and R_2), P (P_1 and P_2) are point attractors, D is a cyclic attractor. Dynamical trajectories of Caspase 8/10, Bid, Fas, VEGF/VEGFR, IL10, Ras and STAT3 is showed in Supplementary Figure S1.

Supplementary Table S7: Attractors of working endogenous network when *n* = 4, *a* = 16

Components	A_1	A_2	A_3	R_1	R_2	P_1	P_2	D
Rb	1.0000	1.0000	1.0000	0.9997	0.0737	0.0805	0.0400	0.9995
Cyclin D/ Cdk4,6	0.0000	0.0000	(0.0000)	0.0659	0.9414	0.0659	0.9414	0.0000
Cyclin E/ Cdk2	0.0000	0.0000	0.0000	0.0093	0.0094	0.9192	0.9194	0.0000
Myc	0.0003	0.0002	0.0000	0.1556	0.1561	0.1563	0.1568	0.0000
E2F	0.0000	0.0000	0.0000	0.0005	0.0094	0.9186	0.9194	0.0000
p21	0.0000	0.0000	0.9195	0.0583	0.0595	0.0595	0.0600	0.9233
p27	0.9773	0.9695	0.9695	0.0564	0.0499	0.0336	0.0297	0.9775
p53	0.0000	0.0000	0.0000	0.0135	0.0135	0.0567	0.0566	0.9312
Caspase 3,7	0.0000	0.0000	0.0000	0.0321	0.0322	0.0321	0.0322	0.0737
Cytochrome c	0.0000	(0.0000)	(0.0000)	0.0125	0.0126	0.0321	0.0321	0.9588
Caspase 8,10	0.0000	0.0000	(0.0000)	0.4672	0.4680	0.4672	0.4679	0.4761
XIAP	0.0002	0.0002	0.0002	0.9395	0.9396	0.9395	0.9396	0.0000
Bcl-2	0.0001	0.0000	0.0000	0.1538	0.1502	0.1538	0.1501	0.0019
Bcl-xL	0.0000	0.0000	(0.0000)	0.9514	0.9514	0.9513	0.9512	0.0000
Bid	0.0000	0.0000	0.0000	0.4325	0.4342	0.4325	0.4341	0.0152
Bad	0.0000	0.0000	0.0000	0.0000	0.0000	0.0000	0.0000	0.9232
Bax	0.0000	0.0000	0.0000	0.0007	0.0007	0.0007	0.0007	0.9234
Ras	0.0000	0.0000	0.0000	0.9813	0.9821	0.9813	0.9821	0.0464
Akt	0.0554	0.0554	0.0554	0.9882	0.9887	0.9882	0.9887	0.0555
PTEN	0.9998	0.9998	0.9998	0.0605	0.0319	0.0605	0.0319	0.9998
ERK	(0.0000)	(0.0000)	(0.0000)	0.4311	0.4324	0.4311	0.4324	0.0082
JNK/p38	0.0000	0.0000	0.0000	0.4335	0.4336	0.4335	0.4336	0.0027
MKP	0.0000	(0.0000)	(0.0000)	0.5277	0.5295	0.5277	0.5295	0.0000
VEGF/ VEGFR	0.0002	0.0002	0.0002	0.9821	0.9828	0.9821	0.9828	0.0157
EGF/EGFR	(0.0000)	(0.0000)	0.0000	0.9345	0.9353	0.9345	0.9353	0.0000
IGF/IGFR	0.0000	0.0000	0.0000	0.9257	0.9258	0.9255	0.9256	0.0000
HGF/c-Met	0.0000	0.0000	0.0000	0.9287	0.9653	0.9286	0.9653	0.0000
Integrin/FAK	0.0000	0.0000	(0.0000)	0.9600	0.9631	0.9600	0.9631	0.0000
E-cadherin	1.0000	1.0000	1.0000	0.0286	0.0269	0.0286	0.0269	0.9995
Zeb1/2	0.0000	0.0000	(0.0000)	0.8470	0.8467	0.8469	0.8466	0.0000
β-catenin	0.0000	0.0000	(0.0000)	0.0689	0.9803	0.0689	0.9802	0.0000
HIF	0.0002	0.0002	0.0002	0.9395	0.9396	0.9394	0.9395	0.0000
NFκB	0.0000	(0.0000)	(0.0000)	0.3609	0.3613	0.3609	0.3612	0.0000
iκB	0.0000	(0.0000)	(0.0000)	0.0260	0.0260	0.0260	0.0260	0.0000

(*Continued*)

Components	A_1	A_2	A_3	R_1	R_2	P_1	P_2	D
TNF-α	0.0000	(0.0000)	(0.0000)	0.4418	0.4422	0.4418	0.4422	0.0000
IL10	0.0000	0.0000	(0.0000)	0.3748	0.3756	0.3748	0.3756	0.4761
IL1	0.0000	(0.0000)	(0.0000)	0.3594	0.3597	0.3594	0.3597	0.0000
IL8	0.0000	(0.0000)	(0.0000)	0.4555	0.4560	0.4554	0.4560	0.0000
Fas	0.0000	0.0000	(0.0000)	0.0139	0.0139	0.0139	0.0139	0.7964
COX2	0.0000	(0.0000)	(0.0000)	0.4555	0.4560	0.4554	0.4560	0.0000
TGF-β	0.0000	0.0000	0.0000	0.7574	0.7569	0.7575	0.7570	0.0000
IFN-γ	(0.0000)	(0.0000)	(0.0000)	0.1602	0.1602	0.1602	0.1602	0.0000
STAT3	0.0000	0.0000	0.0000	0.1534	0.1559	0.1533	0.1559	0.0152
GSK3β	0.9998	0.9998	0.9998	0.0605	0.0604	0.0605	0.0604	0.9998
Gastrin	(0.0000)	(0.0000)	0.0000	0.0004	0.9366	0.0004	0.9366	0.0000
Sox2	0.9196	(0.0000)	(0.0000)	0.9516	0.0093	0.9516	0.0093	0.9220
Cdx2	(0.0000)	(0.0000)	0.9196	0.0000	0.9664	0.0000	0.9664	0.0000
Shh	0.0541	0.0000	(0.0000)	0.9290	0.0000	0.9290	0.0000	0.0543

A (A_1, A_2 and A_3), R (R_1 and R_2), P (P_1 and P_2) are point attractors, D is a cyclic attractor. Dynamical trajectories of Caspase 8/10, Bid, Fas, VEGF/VEGFR, IL10, Ras and STAT3 is showed in Supplementary Figure S1.

Supplementary Table S8: Attractors of working endogenous network when *n* = 5, a = 32

Components	A_1	A_2	A_3	R_1	R_2	P_1	P_2	D
Rb	1.0000	1.0000	1.0000	1.0000	0.0352	0.0362	0.0182	1.0000
Cyclin D/ Cdk4,6	0.0000	(0.0000)	0.0000	0.0320	0.9696	0.0320	0.9696	0.0000
Cyclin E/ Cdk2	0.0000	(0.0000)	0.0000	0.0001	0.0001	0.9638	0.9638	0.0000
Myc	0.0000	0.0000	0.0000	0.0707	0.0706	0.0709	0.0708	0.0000
E2F	0.0000	(0.0000)	0.0000	0.0000	0.0001	0.9638	0.9638	0.0000
p21	0.0000	(0.0000)	0.9638	0.0304	0.0307	0.0307	0.0308	0.9990
p27	0.9891	0.9846	0.9846	0.0299	0.0284	0.0165	0.0157	0.9997
p53	0.0000	(0.0000)	0.0000	0.0047	0.0047	0.0300	0.0300	0.9990
Caspase 3,7	0.0000	(0.0000)	0.0000	0.0121	0.0121	0.0121	0.0121	0.0010
Cytochrome c	0.0000	(0.0000)	0.0000	0.0022	0.0022	0.0162	0.0162	0.9997
Caspase 8,10	0.0000	(0.0000)	0.0000	0.4387	0.4389	0.4387	0.4389	0.2113
XIAP	0.0000	0.0000	0.0000	0.9691	0.9691	0.9691	0.9691	0.0000
Bcl-2	0.0000	(0.0000)	(0.0000)	0.0703	0.0692	0.0703	0.0692	0.0005
Bcl-xL	0.0000	(0.0000)	0.0000	0.9818	0.9818	0.9818	0.9818	0.0000
Bid	0.0000	(0.0000)	0.0000	0.3422	0.3426	0.3422	0.3426	0.5745
Bad	0.0000	0.0000	0.0000	0.0000	0.0000	0.0000	0.0000	0.9990
Bax	0.0000	(0.0000)	0.0000	0.0000	0.0000	0.0000	0.0000	0.9990

(*Continued*)

Components	A_1	A_2	A_3	R_1	R_2	P_1	P_2	D
Ras	0.0000	(0.0000)	0.0000	0.9913	0.9915	0.9913	0.9915	0.9754
Akt	0.0294	0.0294	0.0294	0.9946	0.9947	0.9946	0.9947	0.0010
PTEN	1.0000	1.0000	1.0000	0.0309	0.0159	0.0309	0.0159	1.0000
ERK	0.0000	0.0000	0.0000	0.4420	0.4425	0.4420	0.4425	0.0010
JNK/p38	0.0000	(0.0000)	0.0000	0.4432	0.4431	0.4432	0.4431	0.0010
MKP	0.0000	(0.0000)	0.0000	0.5208	0.5215	0.5208	0.5215	0.0000
VEGF/ VEGFR	0.0000	0.0000	0.0000	0.9914	0.9916	0.9914	0.9916	0.9911
EGF/EGFR	0.0000	0.0000	0.0000	0.9673	0.9675	0.9673	0.9675	0.0000
IGF/IGFR	0.0000	0.0000	0.0000	0.9647	0.9647	0.9647	0.9647	0.0000
HGF/c-Met	0.0000	0.0000	0.0000	0.9654	0.9832	0.9654	0.9832	0.0000
Integrin/FAK	0.0000	(0.0000)	(0.0000)	0.9818	0.9826	0.9818	0.9826	0.0000
E-cadherin	1.0000	1.0000	1.0000	0.0126	0.0122	0.0126	0.0122	1.0000
Zeb1/2	0.0000	(0.0000)	0.0000	0.9294	0.9295	0.9294	0.9295	0.0000
β-catenin	0.0000	(0.0000)	(0.0000)	0.0328	0.9910	0.0328	0.9910	0.0000
HIF	0.0000	0.0000	0.0000	0.9691	0.9691	0.9691	0.9691	0.0000
NFκB	0.0000	(0.0000)	0.0000	0.3536	0.3535	0.3536	0.3535	0.0000
iκB	0.0000	0.0000	0.0000	0.0122	0.0122	0.0122	0.0122	0.0000
TNF-α	0.0000	0.0000	0.0000	0.4515	0.4516	0.4515	0.4516	0.0000
IL10	0.0000	(0.0000)	0.0000	0.3747	0.3750	0.3747	0.3750	0.2113
IL1	0.0000	0.0000	0.0000	0.3554	0.3553	0.3554	0.3553	0.0000
IL8	0.0000	0.0000	0.0000	0.4200	0.4198	0.4200	0.4198	0.0000
Fas	0.0000	(0.0000)	0.0000	0.0048	0.0048	0.0048	0.0048	0.0702
COX2	0.0000	0.0000	0.0000	0.4200	0.4198	0.4200	0.4198	0.0000
TGF-β	0.0000	0.0000	0.0000	0.8349	0.8351	0.8349	0.8351	0.0000
IFN-γ	0.0000	0.0000	0.0000	0.1243	0.1240	0.1243	0.1240	0.0000
STAT3	0.0000	(0.0000)	0.0000	0.0702	0.0706	0.0702	0.0706	0.5745
GSK3β	1.0000	1.0000	1.0000	0.0309	0.0309	0.0309	0.0309	1.0000
Gastrin	0.0000	0.0000	0.0000	0.0000	0.9683	0.0000	0.9683	0.0000
Sox2	0.9638	0.0000	0.0000	0.9816	0.0001	0.9816	0.0001	0.9995
Cdx2	0.0000	0.0000	0.9638	0.0000	0.9836	0.0000	0.9836	0.0000
Shh	0.0292	0.0000	0.0000	0.9668	0.0000	0.9668	0.0000	0.0010

A (A_1, A_2 and A_3), R (R_1 and R_2), P (P_1 and P_2) are point attractors, D is a cyclic attractor. Dynamical trajectories of Caspase 8/10, Bid, Fas, VEGF/VEGFR, IL10, Ras and STAT3 is showed in Supplementary Figure S1.

Supplementary Table S9: Attractors of working endogenous network when *n* = 10, a = 1024

Components	A_1	A_2	A_3	R_1	R_2	P_1	P_2	D
Rb	1.0000	1.0000	1.0000	1.0000	0.0010	0.0010	0.0005	1.0000
Cyclin D/ Cdk4,6	0.0000	0.0000	(0.0000)	0.0010	0.9990	0.0010	0.9990	0.0000
Cyclin E/ Cdk2	0.0000	0.0000	(0.0000)	0.0000	0.0000	0.9990	0.9990	0.0000
Myc	0.0000	0.0000	0.0000	0.0012	0.0012	0.0012	0.0012	0.0000
E2F	0.0000	0.0000	(0.0000)	0.0000	0.0000	0.9990	0.9990	0.0000
p21	0.0000	0.0000	0.9990	0.0010	0.0010	0.0010	0.0010	0.9990
p27	0.9997	0.9995	0.9995	0.0010	0.0010	0.0005	0.0005	0.9997
p53	0.0000	0.0000	(0.0000)	0.0000	0.0000	0.0010	0.0010	0.9990
Caspase 3,7	0.0000	0.0000	(0.0000)	0.0001	0.0001	0.0001	0.0001	0.0010
Cytochrome c	0.0000	0.0000	(0.0000)	0.0000	0.0000	0.0005	0.0005	0.9997
Caspase 8,10	0.0000	0.0000	0.0000	0.3925	0.3925	0.3925	0.3925	0.2113
XIAP	0.0000	0.0000	0.0000	0.9990	0.9990	0.9990	0.9990	0.0000
Bcl-2	0.0000	0.0000	(0.0000)	0.0012	0.0012	0.0012	0.0012	0.0005
Bcl-xL	0.0000	0.0000	(0.0000)	0.9995	0.9995	0.9995	0.9995	0.0000
Bid	0.0000	0.0000	(0.0000)	0.0817	0.0816	0.0817	0.0816	0.5745
Bad	0.0000	0.0000	0.0000	0.0000	0.0000	0.0000	0.0000	0.9990
Bax	0.0000	0.0000	0.0000	0.0000	0.0000	0.0000	0.0000	0.9990
Ras	0.0000	0.0000	0.0000	0.9998	0.9998	0.9998	0.9998	0.9754
Akt	0.0010	0.0010	0.0010	0.9999	0.9999	0.9999	0.9999	0.0010
PTEN	1.0000	1.0000	1.0000	0.0010	0.0005	0.0010	0.0005	1.0000
ERK	0.0000	(0.0000)	(0.0000)	0.4677	0.4677	0.4677	0.4677	0.0010
JNK/p38	0.0000	0.0000	0.0000	0.4678	0.4677	0.4678	0.4677	0.0010
MKP	0.0000	0.0000	(0.0000)	0.5065	0.5065	0.5065	0.5065	0.0000
VEGF/ VEGFR	0.0000	0.0000	0.0000	0.9998	0.9998	0.9998	0.9998	0.9911
EGF/EGFR	0.0000	0.0000	0.0000	0.9990	0.9990	0.9990	0.9990	0.0000
IGF/IGFR	0.0000	0.0000	0.0000	0.9990	0.9990	0.9990	0.9990	0.0000
HGF/c-Met	0.0000	0.0000	0.0000	0.9990	0.9995	0.9990	0.9995	0.0000
Integrin/FAK	0.0000	(0.0000)	(0.0000)	0.9995	0.9995	0.9995	0.9995	0.0000
E-cadherin	1.0000	1.0000	1.0000	0.0003	0.0003	0.0003	0.0003	1.0000
Zeb1/2	0.0000	0.0000	(0.0000)	0.9988	0.9988	0.9988	0.9988	0.0000
β-catenin	0.0000	(0.0000)	0.0000	0.0010	0.9998	0.0010	0.9998	0.0000
HIF	0.0000	0.0000	0.0000	0.9990	0.9990	0.9990	0.9990	0.0000
NFκB	0.0000	(0.0000)	0.0000	0.3393	0.3392	0.3393	0.3392	0.0000
iκB	0.0000	0.0000	(0.0000)	0.0003	0.0003	0.0003	0.0003	0.0000

(*Continued*)

Components	A_1	A_2	A_3	R_1	R_2	P_1	P_2	D
TNF-α	0.0000	0.0000	(0.0000)	0.4770	0.4770	0.4770	0.4770	0.0000
IL10	0.0000	0.0000	0.0000	0.3844	0.3844	0.3844	0.3844	0.2113
IL1	0.0000	0.0000	(0.0000)	0.3408	0.3407	0.3408	0.3407	0.0000
IL8	0.0000	0.0000	(0.0000)	0.3482	0.3481	0.3482	0.3481	0.0000
Fas	0.0000	0.0000	0.0000	0.0000	0.0000	0.0000	0.0000	0.0702
COX2	0.0000	0.0000	(0.0000)	0.3482	0.3481	0.3482	0.3481	0.0000
TGF-β	0.0000	0.0000	0.0000	0.9793	0.9793	0.9793	0.9793	0.0000
IFN-γ	0.0000	0.0000	0.0000	0.0198	0.0197	0.0198	0.0197	0.0000
STAT3	0.0000	0.0000	0.0000	0.0012	0.0012	0.0012	0.0012	0.5745
GSK3β	1.0000	1.0000	1.0000	0.0010	0.0010	0.0010	0.0010	1.0000
Gastrin	(0.0000)	0.0000	(0.0000)	0.0000	0.9990	0.0000	0.9990	0.0000
Sox2	0.9990	0.0000	(0.0000)	0.9995	0.0000	0.9995	0.0000	0.9995
Cdx2	(0.0000)	0.0000	0.9990	0.0000	0.9995	0.0000	0.9995	0.0000
Shh	0.0010	(0.0000)	(0.0000)	0.9990	0.0000	0.9990	(0.0000)	0.0010

A (A_1, A_2 and A_3), R (R_1 and R_2), P (P_1 and P_2) are point attractors, D is a cyclic attractor. Dynamical trajectories of Caspase 8/10, Bid, Fas, VEGF/VEGFR, IL10, Ras and STAT3 is showed in Supplementary Figure S1.

Supplementary Table S10: Saddle points of working endogenous network when $n = 3$, a = 10

Components	S_1	S_2	S_3	S_4	S_5	S_6	S_7
**Neighbor stable states**	A_1,R_1	R_1,P_1	R_1,R_2	R_2,P_2	P_1,P_2	R_1,R_2,P_1,P_2	A_1,A_2
Rb	0.9713	0.2566	0.3048	0.1449	0.1070	0.2357	1.0000
Cyclin D/Cdk4,6	0.0682	0.1223	0.6066	0.8246	0.5952	0.6040	0.0000
Cyclin E/Cdk2	0.0466	0.6601	0.1654	0.3066	0.8542	0.4697	0.0000
Myc	0.2248	0.2611	0.2615	0.2634	0.2610	0.2612	0.0064
E2F	0.0095	0.5668	0.1295	0.2984	0.8446	0.4166	0.0000
p21	0.3934	0.0891	0.0883	0.0893	0.0902	0.0886	0.0000
p27	0.3875	0.0651	0.0675	0.0639	0.0436	0.0620	0.9479
p53	0.1183	0.0697	0.0391	0.0463	0.0841	0.0560	0.0000
Caspase 3,7	0.1363	0.0705	0.0704	0.0704	0.0704	0.0704	0.0000
Cytochrome c	0.1164	0.0452	0.0383	0.0394	0.0500	0.0415	0.0000
Caspase 8,10	0.3988	0.5272	0.5287	0.5290	0.5277	0.5285	0.0000
XIAP	0.5040	0.9056	0.9072	0.9076	0.9064	0.9070	0.0059
Bcl-2	0.2943	0.2496	0.2433	0.2432	0.2407	0.2428	0.0006
Bcl-xL	0.6838	0.7945	0.7944	0.7918	0.7946	0.7947	(0.0000)
Bid	0.3880	0.5943	0.5964	0.5969	0.5951	0.5961	(0.0000)
Bad	0.0074	0.0003	0.0001	0.0001	0.0005	0.0002	0.0000

(*Continued*)

**Components**	**S_1**	**S_2**	**S_3**	**S_4**	**S_5**	**S_6**	**S_7**
**Neighbor stable states**	**A_1,R_1**	**R_1,P_1**	**R_1,R_2**	**R_2,P_2**	**P_1,P_2**	**R_1,R_2,P_1,P_2**	**A_1,A_2**
Bax	0.0259	0.0249	0.0247	0.0254	0.0252	0.0248	0.0000
Ras	0.9152	0.9693	0.9702	0.9707	0.9702	0.9702	0.0000
Akt	0.4465	0.9730	0.9779	0.9798	0.9778	0.9779	0.0840
PTEN	0.4856	0.0934	0.0701	0.0556	0.0709	0.0703	0.9941
ERK	0.3193	0.3805	0.3826	0.3833	0.3826	0.3826	0.0000
JNK/p38	0.3265	0.3848	0.3853	0.3855	0.3853	0.3853	0.0000
MKP	0.4025	0.5285	0.5310	0.5318	0.5309	0.5310	0.0000
VEGF/VEGFR	0.8706	0.9721	0.9731	0.9735	0.9729	0.9730	0.0059
EGF/EGFR	0.4663	0.9030	0.9050	0.9058	0.9048	0.9049	0.0000
IGF/IGFR	0.5868	0.8781	0.8819	0.8815	0.8755	0.8805	0.0000
HGF/c-Met	0.6447	0.8887	0.9199	0.9381	0.9182	0.9195	0.0000
Integrin/FAK	0.3668	0.9274	0.9350	0.9385	0.9347	0.9350	(0.0000)
E-cadherin	0.1373	0.0483	0.0462	0.0450	0.0464	0.0462	1.0000
Zeb1/2	0.6598	0.7516	0.7523	0.7519	0.7500	0.7518	0.0000
β-catenin	0.1011	0.1314	0.6997	0.8921	0.6897	0.6974	(0.0000)
HIF	0.5288	0.9049	0.9086	0.9088	0.9037	0.9076	0.0059
NFκB	0.2513	0.3614	0.3633	0.3635	0.3614	0.3629	0.0000
iκB	0.1025	0.0441	0.0440	0.0438	0.0438	0.0439	0.0000
TNF-α	0.3557	0.4066	0.4073	0.4075	0.4069	0.4072	0.0000
IL10	0.3026	0.3711	0.3721	0.3723	0.3717	0.3720	0.0000
IL1	0.2772	0.3537	0.3545	0.3547	0.3539	0.3544	0.0000
IL8	0.3363	0.5102	0.5125	0.5130	0.5107	0.5121	0.0000
Fas	0.0193	0.0355	0.0356	0.0356	0.0355	0.0356	0.0000
COX2	0.3363	0.5102	0.5125	0.5130	0.5107	0.5121	0.0000
TGF-β	0.5727	0.6379	0.6354	0.6352	0.6384	0.6360	0.0000
IFNγ	0.1375	0.2030	0.2035	0.2035	0.2029	0.2033	0.0000
STAT3	0.2776	0.2606	0.2674	0.2690	0.2645	0.2667	(0.0000)
GSK3β	0.4881	0.0936	0.0923	0.0918	0.0924	0.0923	0.9941
Gastrin	0.0102	0.0222	0.7740	0.8765	0.7664	0.7723	0.0000
Sox2	0.8539	0.8554	0.3328	0.1960	0.3385	0.3342	0.4124
Cdx2	0.0014	0.0031	0.6240	0.8663	0.6106	0.6209	0.0000
Shh	0.3984	0.8552	0.0784	0.0093	0.0851	0.0799	0.0381

(*Continued*)

**Components**	**S_8**	**S_9**	**S_{10}**	**S_{11}**	**S_{12}**	**S_{13}**	**S_{14}**
**Neighbor stable states**	**A_1,A_2**	**A_2,A_3**	**A_2,A_3**	**A_1,D**	**A_2,D**	**A_3,D**	**A_3,D**
Rb	0.7654	0.9591	1.0000	0.7654	0.7654	0.9984	0.9834
Cyclin D/Cdk4,6	0.0000	0.0000	0.0000	0.0003	0.0000	0.0000	0.0003
Cyclin E/Cdk2	0.0000	0.0000	0.0000	0.0000	0.0000	0.0000	0.0000
Myc	0.0027	0.0019	0.0035	0.0053	0.0024	0.0007	0.0010
E2F	0.0000	0.0000	0.0000	0.0000	0.0000	0.0000	0.0000
p21	0.3115	0.5338	0.4102	0.3115	0.3115	0.8694	0.8679
p27	0.9336	0.9437	0.9464	0.9503	0.9309	0.9463	0.9554
p53	0.3571	0.3574	0.0000	0.3571	0.3571	0.3575	0.8832
Caspase 3,7	0.3129	0.1622	(0.0000)	0.3129	0.3129	0.0540	0.1190
Cytochrome c	0.3824	0.3828	(0.0000)	0.3824	0.3824	0.3829	0.9274
Caspase 8,10	0.2344	0.2354	(0.0000)	0.2344	0.2344	0.2356	0.4243
XIAP	0.0027	0.0036	0.0059	0.0027	0.0027	0.0038	0.0007
Bcl-2	0.0003	0.0001	0.0000	0.0029	0.0000	0.0001	0.0333
Bcl-xL	0.0000	0.0000	(0.0000)	0.0000	0.0000	0.0000	0.0000
Bid	0.1141	0.1155	(0.0000)	0.1141	0.1141	0.1157	0.4330
Bad	0.3115	0.3117	(0.0000)	0.3115	0.3115	0.3117	0.8660
Bax	0.3128	0.3134	0.0000	0.3128	0.3128	0.3135	0.8729
Ras	0.0001	0.0001	0.0000	0.0001	0.0001	0.0001	0.4766
Akt	0.0752	0.0829	0.0840	0.0752	0.0752	0.0839	0.0850
PTEN	0.9958	0.9943	0.9941	0.9958	0.9958	0.9941	0.9939
ERK	0.0013	0.0014	(0.0000)	0.0013	0.0013	0.0014	0.0605
JNK/p38	0.0000	0.0000	0.0000	0.0000	0.0000	0.0000	0.0441
MKP	0.0000	0.0000	(0.0000)	0.0000	0.0000	0.0000	0.0031
VEGF/VEGFR	0.0188	0.0207	0.0059	0.0188	0.0188	0.0209	0.4499
EGF/EGFR	(0.0000)	(0.0000)	(0.0000)	0.0000	0.0000	(0.0000)	0.0000
IGF/IGFR	0.0000	0.0000	0.0000	0.0000	0.0000	0.0000	0.0000
HGF/c-Met	0.0000	0.0000	0.0000	0.0000	0.0000	0.0000	0.0022
Integrin/FAK	0.0000	0.0000	(0.0000)	0.0000	0.0000	0.0000	0.0000
E-cadherin	0.7654	0.9591	1.0000	0.7654	0.7654	0.9984	0.9834
Zeb1/2	0.0000	0.0000	(0.0000)	0.0000	0.0000	0.0000	0.0000
β-catenin	0.0000	0.0000	(0.0000)	0.0000	0.0000	0.0000	0.0000
HIF	0.0029	0.0039	0.0059	0.0029	0.0029	0.0040	0.0008
NFκB	0.0000	0.0000	(0.0000)	0.0000	0.0000	(0.0000)	0.0001
iκB	0.0000	0.0000	(0.0000)	0.0000	0.0000	0.0000	0.0018
TNF-α	0.0000	0.0000	(0.0000)	0.0000	0.0000	0.0000	0.0017

(*Continued*)

IL10	0.2344	0.2354	(0.0000)	0.2344	0.2344	0.2356	0.4243
IL1	0.0000	0.0000	(0.0000)	0.0000	0.0000	(0.0000)	0.0009
IL8	0.0000	0.0000	(0.0000)	0.0000	0.0000	(0.0000)	0.0009
Fas	0.3128	0.3134	(0.0000)	0.3128	0.3128	0.3135	0.4192
COX2	0.0000	0.0000	(0.0000)	0.0000	0.0000	(0.0000)	0.0009
TGF-β	0.0000	0.0000	0.0000	0.0000	0.0000	0.0000	0.0000
IFNγ	(0.0000)	(0.0000)	(0.0000)	0.0000	0.0000	(0.0000)	0.0000
STAT3	0.1141	0.1155	(0.0000)	0.1141	0.1141	0.1157	0.4330
GSK3β	0.9958	0.9943	0.9941	0.9958	0.9958	0.9941	0.9939
Gastrin	(0.0000)	0.0000	(0.0000)	0.0000	0.0000	0.0000	0.0000
Sox2	0.4055	0.0152	(0.0000)	0.8675	0.0147	0.0153	0.8860
Cdx2	(0.0000)	0.4126	0.4126	0.0000	0.0000	0.8669	0.0000
Shh	0.0368	0.0000	0.0000	0.0798	0.0000	0.0000	0.0808

Supplementary Table S11: Saddle points of working endogenous network when *n* = 4, a = 16

**Components**	S_1	S_2	S_3	S_4	S_5	S_6	S_7
**Neighbor stable states**	A_1,R_1	R_1,P_1	R_1,R_2	R_2,P_2	P_1,P_2	R_1,R_2,P_1,P_2	A_1,A_2
Rb	0.9997	0.2842	0.6196	0.0687	0.0767	0.2709	1.0000
Cyclin D/Cdk4,6	0.0444	0.0659	0.4426	0.9414	0.4426	0.4426	0.0000
Cyclin E/Cdk2	0.0462	0.6299	0.0090	0.4980	0.9192	0.6003	0.0000
Myc	0.2690	0.1557	0.1544	0.1562	0.1556	0.1546	0.0002
E2F	0.0045	0.5704	0.0027	0.4979	0.9187	0.5528	0.0000
p21	0.3722	0.0585	0.0583	0.0596	0.0595	0.0585	0.0000
p27	0.3890	0.0490	0.0509	0.0471	0.0303	0.0453	0.9704
p53	0.0659	0.0408	0.0135	0.0343	0.0566	0.0393	0.0000
Caspase 3,7	0.0587	0.0321	0.0321	0.0322	0.0321	0.0321	(0.0000)
Cytochrome c	0.0020	0.0241	0.0125	0.0211	0.0321	0.0234	0.0000
Caspase 8,10	0.3170	0.4672	0.4674	0.4680	0.4673	0.4673	(0.0000)
XIAP	0.5078	0.9395	0.9395	0.9396	0.9395	0.9395	0.0002
Bcl-2	0.3317	0.1538	0.1500	0.1502	0.1499	0.1500	0.0000
Bcl-xL	0.5746	0.9514	0.9517	0.9514	0.9514	0.9517	(0.0000)
Bid	0.1392	0.4325	0.4329	0.4341	0.4328	0.4329	(0.0000)
Bad	0.0001	0.0000	0.0000	0.0000	0.0000	0.0000	0.0000
Bax	0.0264	0.0007	0.0006	0.0007	0.0007	0.0006	0.0000
Ras	0.9130	0.9813	0.9815	0.9821	0.9815	0.9815	0.0000
Akt	0.4958	0.9882	0.9883	0.9887	0.9883	0.9883	0.0554
PTEN	0.4922	0.0605	0.0550	0.0319	0.0550	0.0550	0.9998
ERK	0.3854	0.4311	0.4314	0.4324	0.4314	0.4314	0.0000
JNK/p38	0.3814	0.4335	0.4335	0.4336	0.4335	0.4335	0.0000
MKP	0.4089	0.5277	0.5282	0.5295	0.5282	0.5282	0.0000
VEGF/VEGFR	0.8369	0.9821	0.9822	0.9828	0.9822	0.9822	0.0002

(*Continued*)

Components	S_1	S_2	S_3	S_4	S_5	S_6	S_7
Neighbor stable states	A_1,R_1	R_1,P_1	R_1,R_2	R_2,P_2	P_1,P_2	R_1,R_2,P_1,P_2	A_1,A_2
EGF/EGFR	0.2783	0.9345	0.9347	0.9353	0.9347	0.9347	(0.0000)
IGF/IGFR	0.5524	0.9257	0.9258	0.9258	0.9256	0.9257	0.0000
HGF/c-Met	0.6137	0.9287	0.9362	0.9653	0.9362	0.9362	0.0000
Integrin/FAK	0.3625	0.9600	0.9606	0.9631	0.9606	0.9606	(0.0000)
E-cadherin	0.1632	0.0286	0.0282	0.0269	0.0282	0.0282	1.0000
Zeb1/2	0.6289	0.8470	0.8470	0.8467	0.8469	0.8469	(0.0000)
β-catenin	0.0577	0.0689	0.5667	0.9803	0.5667	0.5667	(0.0000)
HIF	0.5271	0.9395	0.9396	0.9396	0.9394	0.9395	0.0002
NFκB	0.2524	0.3609	0.3610	0.3613	0.3609	0.3610	0.0000
iκB	0.1061	0.0260	0.0260	0.0260	0.0260	0.0260	0.0000
TNF-α	0.3936	0.4418	0.4419	0.4422	0.4419	0.4419	0.0000
IL10	0.2773	0.3748	0.3750	0.3756	0.3750	0.3750	(0.0000)
IL1	0.2662	0.3594	0.3595	0.3597	0.3595	0.3595	0.0000
IL8	0.2876	0.4554	0.4555	0.4560	0.4555	0.4555	0.0000
Fas	0.0051	0.0139	0.0139	0.0139	0.0139	0.0139	0.0000
COX2	0.2876	0.4554	0.4555	0.4560	0.4555	0.4555	0.0000
TGF-β	0.5651	0.7574	0.7573	0.7569	0.7574	0.7573	0.0000
IFNγ	0.0679	0.1602	0.1602	0.1602	0.1601	0.1602	0.0000
STAT3	0.2702	0.1533	0.1540	0.1559	0.1540	0.1540	(0.0000)
GSK3β	0.4922	0.0605	0.0605	0.0604	0.0605	0.0605	0.9998
Gastrin	0.0002	0.0004	0.6226	0.9366	0.6226	0.6226	(0.0000)
Sox2	0.9267	0.9516	0.4606	0.0093	0.4606	0.4606	0.5000
Cdx2	0.0000	0.0000	0.3885	0.9664	0.3885	0.3885	(0.0000)
Shh	0.4753	0.9290	0.3067	0.0000	0.3067	0.3067	0.0294

Components	S_8	S_9	S_{10}	S_{11}	S_{12}	S_{13}	S_{14}
Neighbor stable states	A_1,A_2	A_2,A_3	A_2,A_3	A_1,D	A_2,D	A_3,D	A_3,D
Rb	0.8193	0.9935	1.0000	0.8193	0.8193	1.0000	0.9995
Cyclin D/Cdk4,6	0.0000	0.0000	(0.0000)	0.0000	0.0000	0.0000	0.0000
Cyclin E/Cdk2	0.0000	0.0000	(0.0000)	0.0000	0.0000	0.0000	0.0000
Myc	0.0001	0.0000	0.0001	0.0001	0.0001	0.0000	0.0000
E2F	0.0000	0.0000	(0.0000)	0.0000	0.0000	0.0000	0.0000
p21	0.4111	0.6293	0.4999	0.4111	0.4111	0.9238	0.9232
p27	0.9602	0.9692	0.9695	0.9718	0.9586	0.9695	0.9775
p53	0.4571	0.4571	(0.0000)	0.4571	0.4571	0.4571	0.9314
Caspase 3,7	0.3427	0.1423	(0.0000)	0.3427	0.3427	0.0395	0.0741
Cytochrome c	0.4790	0.4790	(0.0000)	0.4790	0.4790	0.4790	0.9602

(*Continued*)

Components	S_8	S_9	S_{10}	S_{11}	S_{12}	S_{13}	S_{14}
Neighbor stable states	A_1,A_2	A_2,A_3	A_2,A_3	A_1,D	A_2,D	A_3,D	A_3,D
Caspase 8,10	0.3138	0.3138	(0.0000)	0.3138	0.3138	0.3138	0.4976
XIAP	0.0001	0.0001	0.0001	0.0001	0.0001	0.0001	0.0000
Bcl-2	0.0000	0.0000	(0.0000)	0.0001	0.0000	0.0000	0.0195
Bcl-xL	(0.0000)	0.0000	(0.0000)	0.0000	0.0000	(0.0000)	0.0000
Bid	0.1342	0.1343	(0.0000)	0.1342	0.1342	0.1343	0.4951
Bad	0.4111	0.4111	0.0000	0.4111	0.4111	0.4111	0.9231
Bax	0.4112	0.4112	(0.0000)	0.4112	0.4112	0.4112	0.9233
Ras	0.0000	0.0000	(0.0000)	0.0000	0.0000	0.0000	0.4803
Akt	0.0517	0.0553	0.0554	0.0517	0.0517	0.0554	0.0557
PTEN	0.9999	0.9999	0.9999	0.9999	0.9999	0.9998	0.9998
ERK	0.0003	0.0003	0.0000	0.0003	0.0003	0.0003	0.0379
JNK/p38	0.0000	0.0000	(0.0000)	0.0000	0.0000	0.0000	0.0283
MKP	0.0000	0.0000	(0.0000)	0.0000	0.0000	0.0000	0.0000
VEGF/VEGFR	0.0053	0.0053	0.0001	0.0053	0.0053	0.0053	0.4902
EGF/EGFR	(0.0000)	(0.0000)	0.0000	(0.0000)	(0.0000)	0.0000	0.0000
IGF/IGFR	0.0000	0.0000	0.0000	0.0000	0.0000	0.0000	0.0000
HGF/c-Met	0.0000	0.0000	0.0000	0.0000	0.0000	0.0000	0.0000
Integrin/FAK	0.0000	0.0000	(0.0000)	0.0000	0.0000	0.0000	0.0000
E-cadherin	0.8193	0.9935	1.0000	0.8193	0.8193	1.0000	0.9995
Zeb1/2	(0.0000)	0.0000	(0.0000)	0.0000	0.0000	(0.0000)	0.0000
β-catenin	0.0000	0.0000	(0.0000)	0.0000	0.0000	0.0000	0.0000
HIF	0.0001	0.0001	0.0001	0.0001	0.0001	0.0001	0.0000
NFκB	0.0000	0.0000	(0.0000)	0.0000	0.0000	0.0000	0.0000
iκB	0.0000	0.0000	(0.0000)	0.0000	0.0000	0.0000	0.0000
TNF-α	0.0000	0.0000	(0.0000)	0.0000	0.0000	0.0000	0.0000
IL10	0.3138	0.3138	(0.0000)	0.3138	0.3138	0.3138	0.4976
IL1	0.0000	0.0000	(0.0000)	0.0000	0.0000	0.0000	0.0000
IL8	0.0000	0.0000	(0.0000)	0.0000	0.0000	0.0000	0.0000
Fas	0.4112	0.4112	(0.0000)	0.4112	0.4112	0.4112	0.4988
COX2	0.0000	0.0000	(0.0000)	0.0000	0.0000	0.0000	0.0000
TGF-β	0.0000	0.0000	0.0000	0.0000	0.0000	0.0000	0.0000
IFNγ	(0.0000)	(0.0000)	0.0000	(0.0000)	(0.0000)	(0.0000)	0.0000
STAT3	0.1342	0.1343	(0.0000)	0.1342	0.1342	0.1343	0.4951
GSK3β	0.9999	0.9999	0.9999	0.9999	0.9999	0.9998	0.9998
Gastrin	(0.0000)	(0.0000)	0.0000	(0.0000)	(0.0000)	0.0000	0.0000
Sox2	0.4987	0.0052	0.0000	0.9197	0.0052	0.0052	0.9276
Cdx2	(0.0000)	0.5000	0.5000	(0.0000)	(0.0000)	0.9196	0.0000
Shh	0.0293	0.0000	0.0000	0.0541	0.0000	0.0000	0.0543

Supplementary Table S12: Saddle points of working endogenous network when *n* = 5, a = 32

Components	S_1	S_2	S_3	S_4	S_5	S_6	S_7
Neighbor stable states	A_1,R_1	R_1,P_1	R_1,R_2	R_2,P_2	P_1,P_2	R_1,R_2,P_1,P_2	A_1,A_2
Rb	1.0000	0.3112	0.6682	0.0340	0.0356	0.2971	1.0000
Cyclin D/Cdk4,6	0.0217	0.0320	0.4347	0.9696	0.4347	0.4347	0.0000
Cyclin E/Cdk2	0.0247	0.5861	0.0001	0.5000	0.9638	0.5666	0.0000
Myc	0.2637	0.0707	0.0703	0.0706	0.0707	0.0704	0.0000
E2F	0.0012	0.5360	0.0000	0.5000	0.9638	0.5275	0.0000
p21	0.3778	0.0304	0.0304	0.0307	0.0307	0.0304	(0.0000)
p27	0.4034	0.0280	0.0285	0.0276	0.0157	0.0270	0.9848
p53	0.0335	0.0191	0.0047	0.0168	0.0300	0.0186	0.0000
Caspase 3,7	0.0232	0.0121	0.0121	0.0121	0.0121	0.0121	0.0000
Cytochrome c	0.0000	0.0103	0.0022	0.0090	0.0162	0.0100	0.0000
Caspase 8,10	0.2809	0.4387	0.4388	0.4389	0.4388	0.4388	(0.0000)
XIAP	0.5041	0.9691	0.9691	0.9691	0.9691	0.9691	0.0000
Bcl-2	0.3345	0.0703	0.0693	0.0692	0.0693	0.0693	0.0000
Bcl-xL	0.5664	0.9818	0.9818	0.9818	0.9818	0.9818	(0.0000)
Bid	0.0530	0.3422	0.3423	0.3426	0.3423	0.3423	(0.0000)
Bad	0.0000	0.0000	0.0000	0.0000	0.0000	0.0000	(0.0000)
Bax	0.0131	0.0000	0.0000	0.0000	0.0000	0.0000	0.0000
Ras	0.9403	0.9913	0.9913	0.9915	0.9913	0.9913	0.0000
Akt	0.4986	0.9946	0.9946	0.9947	0.9946	0.9946	0.0294
PTEN	0.4959	0.0309	0.0293	0.0159	0.0293	0.0293	1.0000
ERK	0.4100	0.4420	0.4421	0.4425	0.4421	0.4421	0.0000
JNK/p38	0.4008	0.4432	0.4432	0.4431	0.4432	0.4432	0.0000
MKP	0.4123	0.5208	0.5209	0.5215	0.5209	0.5209	0.0000
VEGF/VEGFR	0.8349	0.9914	0.9914	0.9916	0.9914	0.9914	0.0000
EGF/EGFR	0.2034	0.9673	0.9673	0.9675	0.9673	0.9673	(0.0000)
IGF/IGFR	0.5344	0.9647	0.9647	0.9647	0.9647	0.9647	0.0000
HGF/c-Met	0.6029	0.9654	0.9674	0.9832	0.9674	0.9674	(0.0000)
Integrin/FAK	0.3670	0.9818	0.9819	0.9826	0.9819	0.9819	(0.0000)
E-cadherin	0.1378	0.0126	0.0125	0.0122	0.0125	0.0125	1.0000
Zeb1/2	0.6408	0.9294	0.9294	0.9295	0.9294	0.9294	(0.0000)
β-catenin	0.0277	0.0328	0.5631	0.9910	0.5631	0.5631	(0.0000)
HIF	0.5140	0.9691	0.9691	0.9691	0.9691	0.9691	0.0000
NFκB	0.2486	0.3536	0.3536	0.3535	0.3536	0.3536	0.0000
iκB	0.1023	0.0122	0.0122	0.0122	0.0122	0.0122	0.0000

(*Continued*)

Components	S_1	S_2	S_3	S_4	S_5	S_6	S_7
Neighbor stable states	A_1,R_1	R_1,P_1	R_1,R_2	R_2,P_2	P_1,P_2	R_1,R_2,P_1,P_2	A_1,A_2
TNF-α	0.4065	0.4515	0.4515	0.4516	0.4515	0.4515	0.0000
IL10	0.2621	0.3747	0.3748	0.3750	0.3748	0.3748	(0.0000)
IL1	0.2564	0.3554	0.3554	0.3553	0.3554	0.3554	0.0000
IL8	0.2654	0.4200	0.4199	0.4198	0.4199	0.4199	0.0000
Fas	0.0012	0.0048	0.0048	0.0048	0.0048	0.0048	(0.0000)
COX2	0.2654	0.4200	0.4199	0.4198	0.4199	0.4199	0.0000
TGF-β	0.5594	0.8349	0.8350	0.8351	0.8350	0.8350	0.0000
IFNγ	0.0329	0.1243	0.1242	0.1240	0.1242	0.1242	(0.0000)
STAT3	0.2623	0.0702	0.0703	0.0706	0.0703	0.0703	(0.0000)
GSK3β	0.4959	0.0309	0.0309	0.0309	0.0309	0.0309	1.0000
Gastrin	0.0000	0.0000	0.6443	0.9683	0.6443	0.6443	0.0000
Sox2	0.9652	0.9816	0.4727	0.0001	0.4727	0.4727	0.5000
Cdx2	0.0000	0.0000	0.3848	0.9836	0.3848	0.3848	(0.0000)
Shh	0.4920	0.9668	0.3388	0.0000	0.3388	0.3388	0.0152

Components	S_8	S_9	S_{10}	S_{11}	S_{12}	S_{13}	S_{14}
Neighbor stable states	A_1,A_2	A_2,A_3	A_2,A_3	A_1,D	A_2,D	A_3,D	A_3,D
Rb	0.8729	0.9994	1.0000	0.8729	0.8729	1.0000	1.0000
Cyclin D/Cdk4,6	0.0000	0.0000	0.0000	0.0000	0.0000	0.0000	0.0000
Cyclin E/Cdk2	0.0000	0.0000	0.0000	0.0000	0.0000	0.0000	0.0000
Myc	0.0000	0.0000	0.0000	0.0000	0.0000	0.0000	0.0000
E2F	0.0000	0.0000	0.0000	0.0000	0.0000	0.0000	0.0000
p21	0.4316	0.6376	0.5000	0.4316	0.4316	0.9647	0.9643
p27	0.9801	0.9846	0.9846	0.9868	0.9797	0.9846	0.9891
p53	0.4732	0.4732	0.0000	0.4732	0.4732	0.4732	0.9669
Caspase 3,7	0.3401	0.1151	0.0000	0.3401	0.3401	0.0181	0.0350
Cytochrome c	0.4894	0.4894	0.0000	0.4894	0.4894	0.4894	0.9819
Caspase 8,10	0.3239	0.3239	0.0000	0.3239	0.3239	0.3239	0.4995
XIAP	0.0000	0.0000	0.0000	0.0000	0.0000	0.0000	0.0000
Bcl-2	0.0000	0.0000	0.0000	0.0000	0.0000	0.0000	0.0090
Bcl-xL	0.0000	0.0000	0.0000	0.0000	0.0000	0.0000	(0.0000)
Bid	0.1024	0.1024	0.0000	0.1024	0.1024	0.1024	0.4988
Bad	0.4316	0.4316	0.0000	0.4316	0.4316	0.4316	0.9643
Bax	0.4316	0.4316	0.0000	0.4316	0.4316	0.4316	0.9643
Ras	0.0000	0.0000	0.0000	0.0000	0.0000	0.0000	0.4927

(*Continued*)

**Components**	**S_8**	**S_9**	**S_{10}**	**S_{11}**	**S_{12}**	**S_{13}**	**S_{14}**
**Neighbor stable states**	**A_1,A_2**	**A_2,A_3**	**A_2,A_3**	**A_1,D**	**A_2,D**	**A_3,D**	**A_3,D**
Akt	0.0285	0.0294	0.0294	0.0285	0.0285	0.0294	0.0294
PTEN	1.0000	1.0000	1.0000	1.0000	1.0000	1.0000	1.0000
ERK	0.0000	0.0000	0.0000	0.0000	0.0000	0.0000	0.0199
JNK/p38	0.0000	0.0000	0.0000	0.0000	0.0000	0.0000	0.0149
MKP	0.0000	0.0000	0.0000	0.0000	0.0000	0.0000	0.0000
VEGF/VEGFR	0.0004	0.0004	0.0000	0.0004	0.0004	0.0004	0.4971
EGF/EGFR	0.0000	0.0000	0.0000	0.0000	0.0000	0.0000	0.0000
IGF/IGFR	0.0000	0.0000	0.0000	0.0000	0.0000	0.0000	0.0000
HGF/c-Met	0.0000	0.0000	0.0000	0.0000	0.0000	0.0000	0.0000
Integrin/FAK	0.0000	0.0000	0.0000	(0.0000)	(0.0000)	0.0000	0.0000
E-cadherin	0.8729	0.9994	1.0000	0.8729	0.8729	1.0000	1.0000
Zeb1/2	0.0000	0.0000	0.0000	0.0000	0.0000	0.0000	(0.0000)
β-catenin	0.0000	(0.0000)	0.0000	(0.0000)	(0.0000)	0.0000	0.0000
HIF	0.0000	0.0000	0.0000	0.0000	0.0000	0.0000	0.0000
NFκB	0.0000	0.0000	0.0000	(0.0000)	(0.0000)	0.0000	0.0000
iκB	0.0000	0.0000	0.0000	0.0000	0.0000	0.0000	0.0000
TNF-α	0.0000	0.0000	0.0000	0.0000	0.0000	0.0000	0.0000
IL10	0.3239	0.3239	0.0000	0.3239	0.3239	0.3239	0.4995
IL1	0.0000	0.0000	0.0000	(0.0000)	0.0000	0.0000	0.0000
IL8	0.0000	0.0000	0.0000	(0.0000)	0.0000	0.0000	0.0000
Fas	0.4316	0.4316	0.0000	0.4316	0.4316	0.4316	0.4998
COX2	0.0000	0.0000	0.0000	(0.0000)	0.0000	0.0000	0.0000
TGF-β	0.0000	0.0000	0.0000	0.0000	0.0000	0.0000	0.0000
IFNγ	0.0000	0.0000	0.0000	0.0000	0.0000	0.0000	0.0000
STAT3	0.1024	0.1024	0.0000	0.1024	0.1024	0.1024	0.4988
GSK3β	1.0000	1.0000	1.0000	1.0000	1.0000	1.0000	1.0000
Gastrin	0.0000	0.0000	0.0000	0.0000	0.0000	(0.0000)	0.0000
Sox2	0.4999	0.0004	0.0000	0.9638	0.0004	0.0004	0.9653
Cdx2	0.0000	0.5000	0.5000	0.0000	0.0000	0.9638	0.0000
Shh	0.0151	0.0000	0.0000	0.0292	0.0000	0.0000	0.0292

Supplementary Table S13: Saddle points of working endogenous network when *n* = 10, a = 1024

Components	S_1	S_2	S_3	S_4	S_5	S_6	S_7
Neighbor stable states	A_1,R_1	R_1,P_1	R_1,R_2	R_2,P_2	P_1,P_2	R_1,R_2,P_1,P_2	A_1,A_2
Rb	1.0000	0.3663	0.8118	0.0010	0.0010	0.3585	1.0000
Cyclin D/Cdk4,6	0.0006	0.0010	0.4320	0.9990	0.4320	0.4320	0.0000
Cyclin E/Cdk2	0.0015	0.5282	0.0000	0.5000	0.9990	0.5227	0.0000
Myc	0.2664	0.0012	0.0012	0.0012	0.0012	0.0012	0.0000
E2F	0.0000	0.5057	0.0000	0.5000	0.9990	0.5046	0.0000
p21	0.3832	0.0010	0.0010	0.0010	0.0010	0.0010	(0.0000)
p27	0.4202	0.0010	0.0010	0.0010	0.0005	0.0010	0.9995
p53	0.0019	0.0005	0.0000	0.0005	0.0010	0.0005	0.0000
Caspase 3,7	0.0000	0.0001	0.0001	0.0001	0.0001	0.0001	(0.0000)
Cytochrome c	0.0000	0.0003	0.0000	0.0002	0.0005	0.0003	0.0000
Caspase 8,10	0.1728	0.3925	0.3925	0.3925	0.3925	0.3925	(0.0000)
XIAP	0.5001	0.9990	0.9990	0.9990	0.9990	0.9990	0.0000
Bcl-2	0.3440	0.0012	0.0012	0.0012	0.0012	0.0012	0.0000
Bcl-xL	0.5126	0.9995	0.9995	0.9995	0.9995	0.9995	0.0000
Bid	0.0000	0.0817	0.0817	0.0816	0.0817	0.0817	(0.0000)
Bad	0.0000	0.0000	0.0000	0.0000	0.0000	0.0000	0.0000
Bax	0.0008	0.0000	0.0000	0.0000	0.0000	0.0000	0.0000
Ras	0.9961	0.9998	0.9998	0.9998	0.9998	0.9998	0.0000
Akt	0.4999	0.9999	0.9999	0.9999	0.9999	0.9999	0.0010
PTEN	0.4999	0.0010	0.0010	0.0005	0.0010	0.0010	1.0000
ERK	0.4536	0.4677	0.4677	0.4677	0.4677	0.4677	0.0000
JNK/p38	0.4522	0.4678	0.4678	0.4677	0.4678	0.4678	0.0000
MKP	0.4265	0.5065	0.5065	0.5065	0.5065	0.5065	(0.0000)
VEGF/VEGFR	0.8694	0.9998	0.9998	0.9998	0.9998	0.9998	0.0000
EGF/EGFR	0.1209	0.9990	0.9990	0.9990	0.9990	0.9990	(0.0000)
IGF/IGFR	0.5026	0.9990	0.9990	0.9990	0.9990	0.9990	0.0000
HGF/c-Met	0.5812	0.9990	0.9990	0.9995	0.9990	0.9990	(0.0000)
Integrin/FAK	0.4094	0.9995	0.9995	0.9995	0.9995	0.9995	(0.0000)
E-cadherin	0.0489	0.0003	0.0003	0.0003	0.0003	0.0003	1.0000
Zeb1/2	0.6546	0.9988	0.9988	0.9988	0.9988	0.9988	0.0000
β-catenin	0.0008	0.0010	0.5484	0.9998	0.5484	0.5484	(0.0000)
HIF	0.5005	0.9990	0.9990	0.9990	0.9990	0.9990	0.0000
NFκB	0.2682	0.3393	0.3393	0.3392	0.3393	0.3393	0.0000
iκB	0.1042	0.0003	0.0003	0.0003	0.0003	0.0003	(0.0000)
TNF-α	0.4271	0.4770	0.4770	0.4770	0.4770	0.4770	(0.0000)

(*Continued*)

**Components**	**S_1**	**S_2**	**S_3**	**S_4**	**S_5**	**S_6**	**S_7**
**Neighbor stable states**	**A_1,R_1**	**R_1,P_1**	**R_1,R_2**	**R_2,P_2**	**P_1,P_2**	**R_1,R_2,P_1,P_2**	**A_1,A_2**
IL10	0.1714	0.3844	0.3844	0.3844	0.3844	0.3844	(0.0000)
IL1	0.2687	0.3408	0.3408	0.3407	0.3408	0.3408	0.0000
IL8	0.2692	0.3482	0.3482	0.3481	0.3482	0.3482	0.0000
Fas	0.0000	0.0000	0.0000	0.0000	0.0000	0.0000	0.0000
COX2	0.2692	0.3482	0.3482	0.3481	0.3482	0.3482	0.0000
TGF-β	0.5330	0.9793	0.9793	0.9793	0.9793	0.9793	0.0000
IFNγ	0.0020	0.0198	0.0198	0.0197	0.0198	0.0198	0.0000
STAT3	0.2828	0.0012	0.0012	0.0012	0.0012	0.0012	(0.0000)
GSK3β	0.4999	0.0010	0.0010	0.0010	0.0010	0.0010	1.0000
Gastrin	0.0000	0.0000	0.7159	0.9990	0.7159	0.7159	(0.0000)
Sox2	0.9990	0.9995	0.4903	0.0000	0.4903	0.4903	0.5000
Cdx2	0.0000	0.0000	0.3970	0.9995	0.3970	0.3970	(0.0000)
Shh	0.4998	0.9990	0.4105	0.0000	0.4105	0.4105	0.0005

**Components**	**S_8**	**S_9**	**S_{10}**	**S_{11}**	**S_{12}**	**S_{13}**	**S_{14}**
**Neighbor stable states**	**A_1,A_2**	**A_2,A_3**	**A_2,A_3**	**A_1,D**	**A_2,D**	**A_3,D**	**A_3,D**
Rb	0.9835	1.0000	1.0000	0.9835	0.9835	1.0000	1.0000
Cyclin D/Cdk4,6	0.0000	(0.0000)	0.0000	0.0000	0.0000	0.0000	0.0000
Cyclin E/Cdk2	(0.0000)	0.0000	0.0000	0.0000	(0.0000)	0.0000	0.0000
Myc	0.0000	0.0000	0.0000	0.0000	0.0000	0.0000	0.0000
E2F	(0.0000)	0.0000	0.0000	0.0000	(0.0000)	0.0000	0.0000
p21	0.4663	0.5000	0.6520	0.4663	0.4663	0.9990	0.9990
p27	0.9995	0.9995	0.9995	0.9997	0.9995	0.9995	0.9997
p53	0.4933	0.0000	0.4933	0.4933	0.4933	0.4933	0.9990
Caspase 3,7	0.3323	0.0000	0.0327	0.3323	0.3323	0.0005	0.0010
Cytochrome c	0.4987	(0.0000)	0.4987	0.4987	0.4987	0.4987	0.9995
Caspase 8,10	0.3321	0.0000	0.3321	0.3321	0.3321	0.3321	0.5000
XIAP	0.0000	0.0000	0.0000	0.0000	0.0000	0.0000	0.0000
Bcl-2	0.0000	0.0000	0.0000	0.0000	0.0000	0.0000	0.0002
Bcl-xL	(0.0000)	0.0000	(0.0000)	0.0000	0.0000	(0.0000)	0.0000
Bid	0.0165	0.0000	0.0165	0.0165	0.0165	0.0165	0.5000
Bad	0.4663	0.0000	0.4663	0.4663	0.4663	0.4663	0.9990
Bax	0.4663	0.0000	0.4663	0.4663	0.4663	0.4663	0.9990
Ras	0.0000	0.0000	0.0000	0.0000	0.0000	0.0000	0.4999
Akt	0.0010	0.0010	0.0010	0.0010	0.0010	0.0010	0.0010

(*Continued*)

**Components**	S_1	S_2	S_3	S_4	S_5	S_6	S_7
**Neighbor stable states**	A_1,R_1	R_1,P_1	R_1,R_2	R_2,P_2	P_1,P_2	R_1,R_2,P_1,P_2	A_1,A_2
PTEN	1.0000	1.0000	1.0000	1.0000	1.0000	1.0000	1.0000
ERK	0.0000	0.0000	0.0000	0.0000	0.0000	0.0000	0.0007
JNK/p38	0.0000	0.0000	0.0000	0.0000	0.0000	0.0000	0.0005
MKP	0.0000	(0.0000)	0.0000	0.0000	0.0000	0.0000	0.0000
VEGF/VEGFR	0.0000	0.0000	0.0000	0.0000	0.0000	0.0000	0.5000
EGF/EGFR	0.0000	0.0000	0.0000	0.0000	0.0000	(0.0000)	0.0000
IGF/IGFR	(0.0000)	0.0000	0.0000	0.0000	(0.0000)	0.0000	(0.0000)
HGF/c-Met	0.0000	(0.0000)	0.0000	0.0000	0.0000	0.0000	0.0000
Integrin/FAK	(0.0000)	0.0000	0.0000	0.0000	(0.0000)	0.0000	0.0000
E-cadherin	0.9835	1.0000	1.0000	0.9835	0.9835	1.0000	1.0000
Zeb1/2	0.0000	0.0000	(0.0000)	0.0000	0.0000	(0.0000)	0.0000
β-catenin	(0.0000)	0.0000	0.0000	0.0000	(0.0000)	0.0000	0.0000
HIF	0.0000	0.0000	0.0000	0.0000	0.0000	0.0000	0.0000
NFκB	0.0000	(0.0000)	0.0000	0.0000	(0.0000)	0.0000	0.0000
iκB	0.0000	(0.0000)	0.0000	0.0000	0.0000	0.0000	0.0000
TNF-α	0.0000	(0.0000)	0.0000	0.0000	0.0000	0.0000	0.0000
IL10	0.3321	0.0000	0.3321	0.3321	0.3321	0.3321	0.5000
IL1	0.0000	(0.0000)	0.0000	0.0000	0.0000	(0.0000)	0.0000
IL8	0.0000	(0.0000)	0.0000	0.0000	0.0000	(0.0000)	0.0000
Fas	0.4663	0.0000	0.4663	0.4663	0.4663	0.4663	0.5000
COX2	0.0000	(0.0000)	0.0000	0.0000	0.0000	(0.0000)	0.0000
TGF-β	(0.0000)	0.0000	0.0000	0.0000	(0.0000)	0.0000	(0.0000)
IFNγ	(0.0000)	0.0000	0.0000	(0.0000)	0.0000	(0.0000)	0.0000
STAT3	0.0165	0.0000	0.0165	0.0165	0.0165	0.0165	0.5000
GSK3β	1.0000	1.0000	1.0000	1.0000	1.0000	1.0000	1.0000
Gastrin	(0.0000)	(0.0000)	(0.0000)	0.0000	(0.0000)	(0.0000)	(0.0000)
Sox2	0.5000	(0.0000)	0.0000	0.9990	0.0000	0.0000	0.9990
Cdx2	0.0000	0.5000	0.5000	0.0000	(0.0000)	0.9990	(0.0000)
Shh	0.0005	(0.0000)	0.0000	0.0010	0.0000	0.0000	0.0010

Supplementary Table S14: Invariant point attractors of ordinary differential equations (ODE) model under random parameter $n_i (3 \leq n_i \leq 10; i = 1, 2, ..., 215)$ and $a_i = 2^{n_i}$ and parameter $n = 3$, $a = 10$

	Random Parameter n_i=[3,10], a=2^{ni}, i=1,2,…,215.							Parameter n=3,a=10						
	A_1	A_2	A_3	R_1	R_2	P_1	P_2	A_1	A_2	A_3	R_1	R_2	P_1	P_2
Rb	1.00	1.00	1.00	1.00	0.00	0.00	0.00	1.00	1.00	1.00	0.95	0.15	0.14	0.08
Cyclin D/Cdk4,6	0.00	(0.00)	0.00	0.00	0.99	0.00	0.99	0.00	0.00	0.00	0.12	0.82	0.12	0.83
Cyclin E/Cdk2	0.00	(0.00)	0.00	0.00	0.00	0.99	0.99	0.00	0.00	0.00	0.15	0.21	0.84	0.86
Myc	0.00	0.00	(0.00)	0.01	0.01	0.01	0.01	0.01	0.01	0.00	0.26	0.26	0.26	0.26
E2F	0.00	(0.00)	0.00	0.00	0.00	0.99	0.99	0.00	0.00	0.00	0.02	0.20	0.82	0.86
p21	0.00	0.00	1.00	0.01	0.01	0.01	0.01	0.00	0.00	0.86	0.09	0.09	0.09	0.09
p27	0.99	0.99	0.99	0.00	0.00	0.00	0.00	0.96	0.95	0.95	0.08	0.07	0.05	0.04
p53	(0.00)	(0.00)	0.00	0.00	0.00	0.06	0.06	0.00	0.00	0.00	0.04	0.04	0.08	0.08
Caspase 3,7	(0.00)	(0.00)	(0.00)	0.01	0.01	0.01	0.01	0.00	0.00	0.00	0.07	0.07	0.07	0.07
Cytochrome *c*	0.00	0.00	(0.00)	0.00	0.00	0.00	0.00	0.00	0.00	0.00	0.04	0.04	0.05	0.05
Caspase 8,10	(0.00)	(0.00)	0.00	0.49	0.49	0.49	0.49	0.00	0.00	0.00	0.53	0.53	0.53	0.53
XIAP	0.00	0.00	0.00	0.94	0.94	0.94	0.94	0.01	0.01	0.01	0.91	0.91	0.91	0.91
Bcl-2	0.00	0.00	(0.00)	0.08	0.07	0.08	0.07	0.01	0.00	0.00	0.25	0.24	0.25	0.24
Bcl-xL	(0.00)	(0.00)	0.00	0.99	0.99	0.99	0.99	0.00	0.00	0.00	0.79	0.79	0.79	0.79
Bid	0.00	(0.00)	0.00	0.44	0.45	0.44	0.45	0.00	0.00	0.00	0.60	0.60	0.59	0.60
Bad	(0.00)	0.00	(0.00)	0.00	0.00	0.00	0.00	0.00	0.00	0.00	0.00	0.00	0.00	0.00
Bax	0.00	0.00	0.00	0.00	0.00	0.00	0.00	0.00	0.00	0.00	0.02	0.03	0.03	0.03
Ras	(0.00)	(0.00)	0.00	1.00	1.00	1.00	1.00	0.00	0.00	0.00	0.97	0.97	0.97	0.97
Akt	0.01	0.01	0.01	1.00	1.00	1.00	1.00	0.08	0.08	0.08	0.97	0.98	0.97	0.98
PTEN	1.00	1.00	1.00	0.03	0.00	0.03	0.00	0.99	0.99	0.99	0.09	0.06	0.09	0.06
ERK	0.00	0.00	(0.00)	0.48	0.48	0.48	0.48	0.00	0.00	0.00	0.38	0.38	0.38	0.38
JNK/p38	0.00	0.00	(0.00)	0.45	0.45	0.45	0.45	0.00	0.00	0.00	0.38	0.39	0.38	0.39
MKP	0.00	(0.00)	0.00	0.51	0.51	0.51	0.51	0.00	0.00	0.00	0.53	0.53	0.53	0.53
VEGF/VEGFR	0.00	0.00	0.00	1.00	1.00	1.00	1.00	0.01	0.01	0.01	0.97	0.97	0.97	0.97
EGF/EGFR	(0.00)	0.00	0.00	0.94	0.94	0.94	0.94	0.00	0.00	0.00	0.90	0.91	0.90	0.91
IGF/IGFR	0.00	(0.00)	(0.00)	1.00	1.00	1.00	1.00	0.00	0.00	0.00	0.88	0.88	0.88	0.88
HGF/c-Met	0.00	0.00	0.00	0.93	1.00	0.93	1.00	0.00	0.00	0.00	0.89	0.94	0.89	0.94
Integrin/FAK	(0.00)	0.00	(0.00)	0.99	0.99	0.99	0.99	0.00	0.00	0.00	0.93	0.94	0.93	0.94
E-cadherin	1.00	1.00	1.00	0.01	0.01	0.01	0.01	1.00	1.00	1.00	0.05	0.04	0.05	0.05
Zeb1/2	(0.00)	(0.00)	0.00	0.94	0.93	0.93	0.93	0.00	0.00	0.00	0.75	0.75	0.75	0.75
β-catenin	0.00	0.00	(0.00)	0.02	1.00	0.02	1.00	0.00	0.00	0.00	0.13	0.89	0.13	0.90
HIF	0.00	0.00	0.00	0.93	0.93	0.93	0.93	0.01	0.01	0.01	0.91	0.91	0.90	0.90
NFκB	0.00	0.00	(0.00)	0.35	0.36	0.35	0.36	0.00	0.00	0.00	0.36	0.36	0.36	0.36
iκB	0.00	0.00	0.00	0.02	0.02	0.02	0.02	0.00	0.00	0.00	0.04	0.04	0.04	0.04
TNF-α	(0.00)	0.00	0.00	0.48	0.48	0.48	0.48	0.00	0.00	0.00	0.41	0.41	0.41	0.41
IL10	(0.00)	(0.00)	0.00	0.43	0.43	0.43	0.43	0.00	0.00	0.00	0.37	0.37	0.37	0.37
IL1	(0.00)	(0.00)	(0.00)	0.33	0.33	0.33	0.33	0.00	0.00	0.00	0.35	0.35	0.35	0.35
IL8	0.00	0.00	(0.00)	0.42	0.42	0.42	0.42	0.00	0.00	0.00	0.51	0.51	0.51	0.51
FasL	(0.00)	0.00	(0.00)	0.01	0.01	0.01	0.01	0.00	0.00	0.00	0.04	0.04	0.04	0.04
COX2	0.00	(0.00)	(0.00)	0.31	0.32	0.31	0.32	0.00	0.00	0.00	0.51	0.51	0.51	0.51
TGF-β	0.00	0.00	0.00	0.88	0.88	0.88	0.88	0.00	0.00	0.00	0.64	0.64	0.64	0.64
IFNγ	(0.00)	(0.00)	0.00	0.07	0.08	0.07	0.08	0.00	0.00	0.00	0.20	0.20	0.20	0.20
STAT3	(0.00)	(0.00)	0.00	0.04	0.04	0.04	0.04	0.00	0.00	0.00	0.26	0.27	0.26	0.27
GSK3β	1.00	1.00	1.00	0.00	0.00	0.00	0.00	0.99	0.99	0.99	0.09	0.09	0.09	0.09
Gastrin	0.00	0.00	0.00	0.00	0.98	0.00	0.98	0.00	0.00	0.00	0.02	0.88	0.02	0.88
Sox2	1.00	(0.00)	0.00	1.00	0.00	1.00	0.00	0.87	0.00	0.00	0.86	0.20	0.86	0.19
Cdx2	0.00	0.00	0.94	0.00	0.99	0.00	0.99	0.00	0.00	0.87	0.00	0.87	0.00	0.88
Shh	0.00	(0.00)	(0.00)	0.99	0.00	0.99	0.00	0.08	0.00	0.00	0.86	0.01	0.86	0.01

Supplementary Table S15: Invariant saddle points of working endogenous network under random parameter $n_i(3 \leq n_i \leq 10; i = 1, 2, ..., 215)$ and $a_i = 2^{n_i}$

Random Parameter n_i=[3,10], a=2^{ni}, i=1,2,…,215.														
**Components**	S_1	S_2	S_3	S_4	S_5	S_6	S_7	S_8	S_9	S_{10}	S_{11}	S_{12}	S_{13}	S_{14}
**Neighbor stable states**	A_1,R_1	R_1,P_1	R_1,R_2	R_2,P_2	P_1,P_2	R_1,R_2,P_1,P_2	A_1,A_2	A_1,A_2	A_2,A_3	A_2,A_3	A_1,D	A_2,D	A_3,D	A_3,D
Rb	0.99	0.22	0.81	0.00	0.00	0.27	0.86	1.00	1.00	1.00	0.86	0.86	1.00	1.00
Cyclin D/Cdk4,6	0.00	0.00	0.42	0.99	0.42	0.42	0.00	0.00	(0.00)	0.00	0.00	0.00	0.00	0.00
Cyclin E/Cdk2	0.01	0.55	0.00	0.50	0.99	0.55	0.00	0.00	(0.00)	0.00	(0.00)	(0.00)	0.00	0.00
Myc	0.33	0.01	0.01	0.01	0.01	0.01	0.00	0.00	0.00	0.00	0.00	0.00	0.00	0.00
E2F	0.00	0.52	0.00	0.50	0.99	0.52	0.00	0.00	0.00	(0.00)	0.00	(0.00)	0.00	0.00
p21	0.35	0.01	0.01	0.01	0.01	0.01	0.45	0.00	0.50	0.65	0.45	0.45	1.00	0.96
p27	0.08	0.00	0.00	0.00	0.00	0.00	0.98	0.99	0.99	0.99	0.99	0.98	0.99	0.99
p53	0.07	0.04	0.00	0.03	0.06	0.04	0.48	0.00	(0.00)	0.48	0.48	0.48	0.48	0.95
Caspase 3,7	0.17	0.01	0.01	0.03	0.01	0.01	0.31	(0.00)	0.00	0.07	0.31	0.31	0.00	0.01
Cytochrome *c*	0.09	0.00	0.00	0.00	0.00	0.00	0.49	(0.00)	0.00	0.49	0.49	0.49	0.49	1.00
Caspase 8,10	0.48	0.49	0.49	0.99	0.49	0.49	0.26	0.00	(0.00)	0.26	0.26	0.26	0.26	0.51
XIAP	0.53	0.94	0.94	0.94	0.94	0.94	0.00	0.00	0.00	0.00	0.00	0.00	0.00	0.00
Bcl-2	0.44	0.08	0.07	0.07	0.07	0.07	0.00	0.00	0.00	(0.00)	0.00	0.00	0.00	0.01
Bcl-xL	0.72	0.99	0.99	0.99	0.99	0.99	0.00	0.00	0.00	0.00	0.00	(0.00)	(0.00)	0.00
Bid	0.42	0.44	0.44	1.00	0.44	0.44	0.00	(0.00)	0.00	0.00	0.00	0.00	0.00	0.56
Bad	0.00	0.00	0.00	0.00	0.00	0.00	0.45	0.00	(0.00)	0.45	0.45	0.45	0.45	0.96
Bax	0.02	0.00	0.00	0.00	0.00	0.00	0.44	0.00	0.00	0.44	0.44	0.44	0.44	0.99
Ras	1.00	1.00	1.00	1.00	1.00	1.00	0.00	(0.00)	0.00	0.00	0.00	0.00	0.00	0.74
Akt	0.49	1.00	1.00	1.00	1.00	1.00	0.01	0.01	0.01	0.01	0.01	0.01	0.01	0.01
PTEN	0.50	0.03	0.03	0.00	0.03	0.03	1.00	1.00	1.00	1.00	1.00	1.00	1.00	1.00
ERK	0.42	0.48	0.48	0.48	0.48	0.48	0.00	0.00	(0.00)	0.00	0.00	0.00	0.00	0.00
JNK/p38	0.46	0.45	0.45	0.45	0.45	0.45	0.00	0.00	0.00	0.00	0.00	0.00	0.00	0.07
MKP	0.39	0.51	0.51	0.51	0.51	0.51	0.00	(0.00)	0.00	0.00	0.00	0.00	(0.00)	0.00
VEGF/VEGFR	0.89	1.00	1.00	1.00	1.00	1.00	0.00	0.00	0.00	0.00	0.00	0.00	0.00	0.56
EGF/EGFR	0.47	0.94	0.94	0.94	0.94	0.94	(0.00)	0.00	(0.00)	(0.00)	0.00	0.00	0.00	0.00
IGF/IGFR	0.59	1.00	1.00	1.00	1.00	1.00	0.00	0.00	0.00	0.00	0.00	0.00	0.00	0.00
HGF/c-Met	0.59	0.93	0.94	1.00	0.94	0.94	0.00	0.00	0.00	0.00	0.00	0.00	0.00	0.00
Integrin/FAK	0.38	0.99	0.99	0.99	0.99	0.99	0.00	0.00	0.00	0.00	0.00	(0.00)	0.00	0.00
E-cadherin	0.19	0.01	0.01	0.01	0.01	0.01	0.88	1.00	1.00	1.00	0.88	0.88	1.00	1.00
Zeb1/2	0.56	0.94	0.94	0.93	0.93	0.94	0.00	(0.00)	(0.00)	0.00	(0.00)	0.00	(0.00)	0.00
β-catenin	0.02	0.02	0.53	1.00	0.53	0.53	0.00	0.00	0.00	0.00	0.00	(0.00)	0.00	0.00
HIF	0.52	0.93	0.93	0.93	0.93	0.93	0.00	0.00	0.00	0.00	0.00	0.00	0.00	0.00
NFκB	0.37	0.35	0.35	0.36	0.35	0.35	0.00	(0.00)	0.00	(0.00)	0.00	(0.00)	(0.00)	0.00
iκB	0.11	0.02	0.02	0.01	0.02	0.02	0.00	0.00	0.00	0.00	0.00	0.00	0.00	0.00
TNF-α	0.48	0.48	0.48	0.61	0.48	0.48	0.00	0.00	(0.00)	0.00	0.00	0.00	(0.00)	0.00
IL10	0.41	0.43	0.43	0.43	0.43	0.43	0.34	0.00	(0.00)	0.34	0.34	0.34	0.34	0.51
IL1	0.34	0.33	0.33	0.33	0.33	0.33	0.00	(0.00)	(0.00)	(0.00)	0.00	(0.00)	(0.00)	0.00
IL8	0.43	0.42	0.42	0.42	0.42	0.42	0.00	0.00	0.00	(0.00)	0.00	0.00	0.00	0.00
FasL	0.01	0.01	0.01	0.05	0.01	0.01	0.45	(0.00)	(0.00)	0.45	0.45	0.45	0.45	0.50
COX2	0.34	0.31	0.31	0.32	0.31	0.31	0.00	(0.00)	0.00	(0.00)	(0.00)	0.00	(0.00)	0.00
TGF-β	0.51	0.88	0.88	0.88	0.88	0.88	0.00	(0.00)	0.00	0.00	0.00	0.00	0.00	0.00
IFNγ	0.10	0.07	0.07	0.51	0.07	0.07	0.00	0.00	(0.00)	0.00	0.00	0.00	(0.00)	0.00
STAT3	0.38	0.04	0.04	0.04	0.04	0.04	0.07	0.00	0.00	0.07	0.07	0.07	0.07	0.52
GSK3β	0.51	0.00	0.00	0.00	0.00	0.00	1.00	1.00	1.00	1.00	1.00	1.00	1.00	1.00
Gastrin	0.00	0.00	0.58	0.98	0.58	0.58	0.00	0.00	0.00	0.00	(0.00)	0.00	(0.00)	0.00
Sox2	1.00	1.00	0.49	0.00	0.49	0.49	0.34	0.50	(0.00)	0.00	1.00	0.00	0.00	1.00
Cdx2	0.00	0.00	0.33	0.99	0.33	0.33	(0.00)	0.00	0.50	0.50	0.00	0.00	0.94	0.00
Shh	0.43	0.99	0.41	(0.00)	0.41	0.41	0.00	0.00	0.00	0.00	0.00	0.00	0.00	0.00

Supplementary Table S16: Invariant point attractors of ordinary differential equations (ODE) model under random parameter $n_i (3 \leq n_i \leq 10; i = 1, 2, ..., 215)$ and $a_i = 2^{n_i}$ and parameter $n = 3$, $a = 10$

	Random Parameter n_i=[1,10], a=2^{ni}, i=1,2,…,215.							Parameter n=3,a=10						
	A_1	A_2	A_3	R_1	R_2	P_1	P_2	A_1	A_2	A_3	R_1	R_2	P_1	P_2
Rb	1.00	1.00	1.00	1.00	0.02	0.01	0.00	1.00	1.00	1.00	0.95	0.15	0.14	0.08
Cyclin D/Cdk4,6	0.00	0.00	0.00	0.03	1.00	0.03	1.00	0.00	0.00	0.00	0.12	0.82	0.12	0.83
Cyclin E/Cdk2	0.00	0.00	0.00	0.00	0.00	1.00	1.00	0.00	0.00	0.00	0.15	0.21	0.84	0.86
Myc	0.00	0.00	0.00	0.00	0.00	0.00	0.00	0.01	0.01	0.00	0.26	0.26	0.26	0.26
E2F	0.00	0.00	0.00	0.00	0.00	0.98	0.98	0.00	0.00	0.00	0.02	0.20	0.82	0.86
p21	0.00	0.00	1.00	0.07	0.07	0.07	0.07	0.00	0.00	0.86	0.09	0.09	0.09	0.09
p27	1.00	1.00	1.00	0.00	0.00	0.00	0.00	0.96	0.95	0.95	0.08	0.07	0.05	0.04
p53	0.00	0.00	0.00	0.01	0.01	0.04	0.04	0.00	0.00	0.00	0.04	0.04	0.08	0.08
Caspase 3,7	0.00	0.00	0.00	0.05	0.05	0.05	0.05	0.00	0.00	0.00	0.07	0.07	0.07	0.07
Cytochrome *c*	0.00	0.00	0.00	0.01	0.01	0.01	0.01	0.00	0.00	0.00	0.04	0.04	0.05	0.05
Caspase 8,10	0.00	0.00	0.00	0.49	0.49	0.49	0.49	0.00	0.00	0.00	0.53	0.53	0.53	0.53
XIAP	0.00	0.00	0.00	0.92	0.92	0.92	0.92	0.01	0.01	0.01	0.91	0.91	0.91	0.91
Bcl-2	0.00	0.00	0.00	0.00	0.00	0.00	0.00	0.01	0.00	0.00	0.25	0.24	0.25	0.24
Bcl-xL	0.00	0.00	0.00	0.98	0.98	0.98	0.98	0.00	0.00	0.00	0.79	0.79	0.79	0.79
Bid	0.00	0.00	0.00	0.49	0.49	0.49	0.49	0.00	0.00	0.00	0.60	0.60	0.59	0.60
Bad	0.00	0.00	0.00	0.00	0.00	0.00	0.00	0.00	0.00	0.00	0.00	0.00	0.00	0.00
Bax	0.00	0.00	0.00	0.00	0.00	0.00	0.00	0.00	0.00	0.00	0.02	0.03	0.03	0.03
Ras	0.00	0.00	0.00	0.98	0.98	0.98	0.98	0.00	0.00	0.00	0.97	0.97	0.97	0.97
Akt	0.04	0.04	0.04	1.00	1.00	1.00	1.00	0.08	0.08	0.08	0.97	0.98	0.97	0.98
PTEN	1.00	1.00	1.00	0.00	0.00	0.00	0.00	0.99	0.99	0.99	0.09	0.06	0.09	0.06
ERK	0.00	0.00	0.00	0.45	0.45	0.45	0.45	0.00	0.00	0.00	0.38	0.38	0.38	0.38
JNK/p38	0.00	0.00	0.00	0.45	0.45	0.45	0.45	0.00	0.00	0.00	0.38	0.39	0.38	0.39
MKP	0.00	0.00	0.00	0.51	0.51	0.51	0.51	0.00	0.00	0.00	0.53	0.53	0.53	0.53
VEGF/VEGFR	0.00	0.00	0.00	1.00	1.00	1.00	1.00	0.01	0.01	0.01	0.97	0.97	0.97	0.97
EGF/EGFR	0.00	0.00	0.00	0.98	0.98	0.98	0.98	0.00	0.00	0.00	0.90	0.91	0.90	0.91
IGF/IGFR	0.00	0.00	0.00	0.98	0.98	0.98	0.98	0.00	0.00	0.00	0.88	0.88	0.88	0.88
HGF/c-Met	0.00	0.00	0.00	0.95	0.97	0.95	0.97	0.00	0.00	0.00	0.89	0.94	0.89	0.94
Integrin/FAK	0.00	0.00	0.00	1.00	1.00	1.00	1.00	0.00	0.00	0.00	0.93	0.94	0.93	0.94
E-cadherin	1.00	1.00	1.00	0.01	0.01	0.01	0.01	1.00	1.00	1.00	0.05	0.04	0.05	0.05
Zeb1/2	0.00	0.00	0.00	0.88	0.88	0.88	0.88	0.00	0.00	0.00	0.75	0.75	0.75	0.75
β-catenin	0.00	0.00	0.00	0.01	1.00	0.01	1.00	0.00	0.00	0.00	0.13	0.89	0.13	0.90
HIF	0.00	0.00	0.00	0.96	0.96	0.95	0.95	0.01	0.01	0.01	0.91	0.91	0.90	0.90
NFκB	0.00	0.00	0.00	0.30	0.30	0.30	0.30	0.00	0.00	0.00	0.36	0.36	0.36	0.36
iκB	0.00	0.00	0.00	0.00	0.00	0.00	0.00	0.00	0.00	0.00	0.04	0.04	0.04	0.04
TNF-α	0.00	0.00	0.00	0.45	0.45	0.45	0.45	0.00	0.00	0.00	0.41	0.41	0.41	0.41
IL10	0.00	0.00	0.00	0.42	0.42	0.42	0.42	0.00	0.00	0.00	0.37	0.37	0.37	0.37
IL1	0.00	0.00	0.00	0.42	0.42	0.42	0.42	0.00	0.00	0.00	0.35	0.35	0.35	0.35
IL8	0.00	0.00	0.00	0.45	0.45	0.45	0.45	0.00	0.00	0.00	0.51	0.51	0.51	0.51
FasL	0.00	0.00	0.00	0.01	0.01	0.01	0.01	0.00	0.00	0.00	0.04	0.04	0.04	0.04
COX2	0.00	0.00	0.00	0.46	0.46	0.46	0.46	0.00	0.00	0.00	0.51	0.51	0.51	0.51
TGF-β	0.00	0.00	0.00	0.98	0.98	0.98	0.98	0.00	0.00	0.00	0.64	0.64	0.64	0.64
IFNγ	0.00	0.00	0.00	0.20	0.20	0.20	0.20	0.00	0.00	0.00	0.20	0.20	0.20	0.20
STAT3	0.00	0.00	0.00	0.18	0.18	0.18	0.18	0.00	0.00	0.00	0.26	0.27	0.26	0.27
GSK3β	1.00	1.00	1.00	0.00	0.00	0.00	0.00	0.99	0.99	0.99	0.09	0.09	0.09	0.09
Gastrin	0.00	0.00	0.00	0.00	1.00	0.00	1.00	0.00	0.00	0.00	0.02	0.88	0.02	0.88
Sox2	0.88	0.00	0.00	0.95	0.04	0.95	0.04	0.87	0.00	0.00	0.86	0.20	0.86	0.19
Cdx2	0.00	0.00	0.99	0.00	1.00	0.00	1.00	0.00	0.00	0.87	0.00	0.87	0.00	0.88
Shh	0.01	0.00	0.00	0.95	0.00	0.95	0.00	0.08	0.00	0.00	0.86	0.01	0.86	0.01

Supplementary Table S17: Invariant saddle points of working endogenous network under random parameter $n_i(3 \leq n_i \leq 10; i = 1, 2, ..., 215)$ and $a_i = 2^{n_i}$

Random Parameter n_i=[1,10], a=2^{n_i}, i=1,2,…,215.													
**Components**	S_1	S_2	S_3	S_4	S_5	S_6	S_7	S_8	S_9	S_{10}	S_{11}	S_{12}	S_{13}
**Neighbor stable states**	A_1,R_1	R_1,P_1	R_1,R_2	R_2,P_2	P_1,P_2	R_1,R_2,P_1,P_2	A_1,A_2	A_1,A_2	A_2,A_3	A_2,A_3	A_1,D	A_2,D	A_3,D
Rb	1.00	0.17	0.83	0.02	0.01	0.16	1.00	0.68	1.00	1.00	0.68	0.68	1.00
Cyclin D/Cdk4,6	0.03	0.03	0.40	1.00	0.52	0.52	0.00	0.00	0.00	(0.00)	0.00	0.00	0.00
Cyclin E/Cdk2	0.00	0.62	0.00	0.50	1.00	0.60	(0.00)	0.00	0.00	0.00	0.00	(0.00)	(0.00)
Myc	0.10	0.00	0.00	0.00	0.00	0.00	0.00	0.00	0.00	0.00	0.00	0.00	0.00
E2F	0.00	0.53	0.00	0.50	0.98	0.52	0.00	(0.00)	0.00	(0.00)	0.00	(0.00)	(0.00)
p21	0.38	0.07	0.07	0.07	0.07	0.07	(0.00)	0.24	0.57	0.50	0.24	0.24	1.00
p27	0.05	0.00	0.00	0.00	0.00	0.00	1.00	1.00	1.00	1.00	1.00	1.00	1.00
p53	0.10	0.03	0.01	0.02	0.04	0.03	0.00	0.44	0.44	(0.00)	0.44	0.44	0.44
Caspase 3,7	0.12	0.05	0.10	0.10	0.05	0.05	0.00	0.43	0.14	0.00	0.43	0.43	0.01
Cytochrome *c*	0.08	0.01	0.01	0.01	0.01	0.01	(0.00)	0.48	0.48	0.00	0.48	0.48	0.48
Caspase 8,10	0.41	0.49	0.84	0.84	0.49	0.49	0.00	0.20	0.20	(0.00)	0.20	0.20	0.20
XIAP	0.50	0.92	0.92	0.92	0.92	0.92	0.00	0.00	0.00	0.00	0.00	0.00	0.00
Bcl-2	0.12	0.00	0.00	0.00	0.00	0.00	0.00	0.00	0.00	0.00	0.00	0.00	0.00
Bcl-xL	0.66	0.98	0.98	0.98	0.98	0.98	(0.00)	(0.00)	(0.00)	(0.00)	0.00	(0.00)	(0.00)
Bid	0.39	0.49	0.79	0.79	0.49	0.49	0.00	0.11	0.11	0.00	0.11	0.11	0.11
Bad	0.00	0.00	0.00	0.00	0.00	0.00	(0.00)	0.35	0.35	(0.00)	0.35	0.35	0.35
Bax	0.03	0.00	0.00	0.00	0.00	0.00	0.00	0.43	0.43	0.00	0.43	0.43	0.43
Ras	0.95	0.98	0.98	0.98	0.98	0.98	0.00	0.00	0.00	0.00	0.00	0.00	0.00
Akt	0.50	1.00	1.00	1.00	1.00	1.00	0.04	0.03	0.04	0.04	0.03	0.03	0.04
PTEN	0.49	0.00	0.00	0.00	0.00	0.00	1.00	1.00	1.00	1.00	1.00	1.00	1.00
ERK	0.45	0.45	0.45	0.45	0.45	0.45	0.00	0.00	0.00	(0.00)	0.00	0.00	0.00
JNK/p38	0.40	0.45	0.45	0.45	0.45	0.45	0.00	0.00	0.00	0.00	0.00	0.00	0.00
MKP	0.44	0.51	0.51	0.51	0.51	0.51	(0.00)	(0.00)	0.00	0.00	(0.00)	0.00	0.00
VEGF/VEGFR	0.87	1.00	1.00	1.00	1.00	1.00	0.00	0.00	0.00	0.00	0.00	0.00	0.00
EGF/EGFR	0.51	0.98	0.98	0.98	0.98	0.98	(0.00)	(0.00)	(0.00)	0.00	0.00	0.00	0.00
IGF/IGFR	0.49	0.98	0.98	0.98	0.98	0.98	0.00	0.00	0.00	0.00	0.00	0.00	0.00
HGF/c-Met	0.61	0.95	0.95	0.97	0.95	0.95	0.00	0.00	0.00	0.00	0.00	0.00	0.00
Integrin/FAK	0.46	1.00	1.00	1.00	1.00	1.00	0.00	(0.00)	(0.00)	(0.00)	0.00	(0.00)	(0.00)
E-cadherin	0.07	0.01	0.01	0.01	0.01	0.01	1.00	0.72	1.00	1.00	0.72	0.72	1.00
Zeb1/2	0.66	0.88	0.88	0.88	0.88	0.88	0.00	(0.00)	0.00	(0.00)	(0.00)	(0.00)	(0.00)
β-catenin	0.01	0.01	0.58	1.00	0.62	0.62	0.00	0.00	(0.00)	0.00	0.00	0.00	0.00
HIF	0.50	0.96	0.96	0.96	0.95	0.96	0.00	0.00	0.00	0.00	0.00	0.00	0.00
NFκB	0.14	0.30	0.30	0.30	0.30	0.30	0.00	(0.00)	0.00	(0.00)	0.00	0.00	(0.00)
iκB	0.11	0.00	0.00	0.00	0.00	0.00	0.00	0.00	0.00	0.00	0.00	0.00	0.00
TNF-α	0.44	0.45	0.70	0.70	0.45	0.45	(0.00)	0.00	0.00	0.00	(0.00)	0.00	0.00
IL10	0.40	0.42	0.38	0.38	0.42	0.42	0.00	0.26	0.26	(0.00)	0.26	0.26	0.26
IL1	0.30	0.42	0.42	0.42	0.42	0.42	0.00	(0.00)	0.00	(0.00)	(0.00)	0.00	(0.00)
IL8	0.30	0.45	0.45	0.45	0.45	0.45	(0.00)	0.00	0.00	0.00	(0.00)	0.00	0.00
FasL	0.00	0.01	0.02	0.02	0.01	0.01	(0.00)	0.38	0.38	(0.00)	0.38	0.38	0.38
COX2	0.27	0.46	0.46	0.46	0.46	0.46	(0.00)	(0.00)	0.00	0.00	(0.00)	0.00	(0.00)
TGF-β	0.61	0.98	0.98	0.98	0.98	0.98	0.00	0.00	0.00	0.00	0.00	0.00	0.00
IFNγ	0.04	0.20	0.51	0.51	0.20	0.20	(0.00)	(0.00)	(0.00)	(0.00)	0.00	(0.00)	0.00
STAT3	0.34	0.18	0.18	0.18	0.18	0.18	0.00	0.10	0.10	0.00	0.10	0.10	0.10
GSK3β	0.50	0.00	0.00	0.00	0.00	0.00	1.00	1.00	1.00	1.00	1.00	1.00	1.00
Gastrin	0.00	0.00	0.88	1.00	0.87	0.87	0.00	(0.00)	0.00	0.00	(0.00)	0.00	0.00
Sox2	0.90	0.95	0.48	0.02	0.47	0.47	0.50	0.50	0.01	0.00	0.88	0.01	0.01
Cdx2	0.00	0.00	0.41	1.00	0.52	0.52	0.00	(0.00)	0.50	0.50	(0.00)	0.00	0.99
Shh	0.48	0.95	0.59	0.00	0.18	0.18	0.01	0.01	0.00	0.00	0.01	0.00	(0.00)

Supplementary Table S18: Consistence between attractors of ordinary diferential equations (ODE) model and Boolean network. Note that there are cyclic attractors in ODE model and Boolean network, the listed are average activity profiles

	Attractors of ODE model							Attractors of Boolean Network						
Endogenous factors	A_1	A_2	A_3	D	R_1	P_1	P_2	A_1	A_2	A_3	D	R_1	P_1	P_2
Rb	1.00	1.00	1.00	0.98	0.95	0.14	0.08	1.00	1.00	1.00	0.67	0.25	0.00	0.00
Cyclin D/Cdk4,6	0.00	0.00	0.00	0.00	0.12	0.12	0.83	0.00	0.00	0.00	0.00	0.50	0.50	0.75
Cyclin E/Cdk2	0.00	0.00	0.00	0.00	0.15	0.84	0.86	0.00	0.00	0.00	0.00	0.25	1.00	1.00
Myc	0.01	0.01	0.00	0.00	0.26	0.26	0.26	0.00	0.00	0.00	0.00	0.25	0.25	0.25
E2F	0.00	0.00	0.00	0.05	0.02	0.82	0.86	0.00	0.00	0.00	0.00	0.00	1.00	1.00
p21	0.00	0.00	0.86	0.87	0.09	0.09	0.09	0.00	0.00	1.00	0.67	0.00	0.00	0.00
p27	0.96	0.95	0.95	0.96	0.08	0.05	0.04	1.00	1.00	1.00	1.00	0.00	0.00	0.00
p53	0.00	0.00	0.00	0.88	0.04	0.08	0.08	0.00	0.00	0.00	0.67	0.00	0.00	0.00
Caspase 3,7	0.00	0.00	0.00	0.12	0.07	0.07	0.07	0.00	0.00	0.00	0.33	0.00	0.00	0.00
Cytochrome c	0.00	0.00	0.00	0.93	0.04	0.05	0.05	0.00	0.00	0.00	0.67	0.00	0.00	0.00
Caspase 8,10	0.00	0.00	0.00	0.42	0.53	0.53	0.53	0.00	0.00	0.00	0.33	0.75	0.75	0.75
XIAP	0.01	0.01	0.01	0.00	0.91	0.91	0.91	0.00	0.00	0.00	0.00	1.00	1.00	1.00
Bcl-2	0.01	0.00	0.00	0.03	0.25	0.25	0.24	0.00	0.00	0.00	0.00	0.50	0.50	0.50
Bcl-xL	0.00	0.00	0.00	0.00	0.79	0.79	0.79	0.00	0.00	0.00	0.00	0.75	0.75	0.75
Bid	0.00	0.00	0.00	0.43	0.60	0.59	0.60	0.00	0.00	0.00	0.33	0.75	0.75	0.75
Bad	0.00	0.00	0.00	0.87	0.00	0.00	0.00	0.00	0.00	0.00	0.67	0.00	0.00	0.00
Bax	0.00	0.00	0.00	0.87	0.02	0.03	0.03	0.00	0.00	0.00	0.67	0.00	0.00	0.00
Ras	0.00	0.00	0.00	0.48	0.97	0.97	0.97	0.00	0.00	0.00	0.33	1.00	1.00	1.00
Akt	0.08	0.08	0.08	0.08	0.97	0.97	0.98	0.00	0.00	0.00	0.00	1.00	1.00	1.00
PTEN	0.99	0.99	0.99	1.00	0.09	0.09	0.06	1.00	1.00	1.00	1.00	0.00	0.00	0.00
ERK	0.00	0.00	0.00	0.06	0.38	0.38	0.38	0.00	0.00	0.00	0.00	0.50	0.50	0.50
JNK/p38	0.00	0.00	0.00	0.04	0.38	0.38	0.39	0.00	0.00	0.00	0.00	0.50	0.50	0.50
MKP	0.00	0.00	0.00	0.00	0.53	0.53	0.53	0.00	0.00	0.00	0.00	0.50	0.50	0.50
VEGF/VEGFR	0.01	0.01	0.01	0.45	0.97	0.97	0.97	0.00	0.00	0.00	0.33	1.00	1.00	1.00
EGF/EGFR	0.00	0.00	0.00	0.00	0.90	0.90	0.91	0.00	0.00	0.00	0.00	1.00	1.00	1.00
IGF/IGFR	0.00	0.00	0.00	0.00	0.88	0.88	0.88	0.00	0.00	0.00	0.00	1.00	1.00	1.00
HGF/c-Met	0.00	0.00	0.00	0.00	0.89	0.89	0.94	0.00	0.00	0.00	0.00	1.00	1.00	1.00
Integrin/FAK	0.00	0.00	0.00	0.00	0.93	0.93	0.94	0.00	0.00	0.00	0.00	1.00	1.00	1.00
E-cadherin	1.00	1.00	1.00	0.98	0.05	0.05	0.05	1.00	1.00	1.00	0.67	0.00	0.00	0.00
Zeb1/2	0.00	0.00	0.00	0.00	0.75	0.75	0.75	0.00	0.00	0.00	0.00	0.75	0.75	0.75
β-catenin	0.00	0.00	0.00	0.00	0.13	0.13	0.90	0.00	0.00	0.00	0.00	0.50	0.50	0.75
HIF	0.01	0.01	0.01	0.00	0.91	0.90	0.90	0.00	0.00	0.00	0.00	1.00	1.00	1.00
NFκB	0.00	0.00	0.00	0.00	0.36	0.36	0.36	0.00	0.00	0.00	0.00	0.50	0.50	0.50
iκB	0.00	0.00	0.00	0.00	0.04	0.04	0.04	0.00	0.00	0.00	0.00	0.00	0.00	0.00
TNF-α	0.00	0.00	0.00	0.00	0.41	0.41	0.41	0.00	0.00	0.00	0.00	0.50	0.50	0.50
IL10	0.00	0.00	0.00	0.42	0.37	0.37	0.37	0.00	0.00	0.00	0.33	0.25	0.25	0.25
IL1	0.00	0.00	0.00	0.00	0.35	0.35	0.35	0.00	0.00	0.00	0.00	0.50	0.50	0.50
IL8	0.00	0.00	0.00	0.00	0.51	0.51	0.51	0.00	0.00	0.00	0.00	0.75	0.75	0.75
Fas	0.00	0.00	0.00	0.42	0.04	0.04	0.04	0.00	0.00	0.00	0.33	0.00	0.00	0.00
COX2	0.00	0.00	0.00	0.00	0.51	0.51	0.51	0.00	0.00	0.00	0.00	0.75	0.75	0.75
TGF-β	0.00	0.00	0.00	0.00	0.64	0.64	0.64	0.00	0.00	0.00	0.00	0.50	0.50	0.50
IFN-γ	0.00	0.00	0.00	0.00	0.20	0.20	0.20	0.00	0.00	0.00	0.00	0.50	0.50	0.50
STAT3	0.00	0.00	0.00	0.43	0.26	0.26	0.27	0.00	0.00	0.00	0.33	0.50	0.50	0.50
GSK3β	0.99	0.99	0.99	0.99	0.09	0.09	0.09	1.00	1.00	1.00	1.00	0.00	0.00	0.00
Gastrin	0.00	0.00	0.00	0.00	0.02	0.02	0.88	0.00	0.00	0.00	0.00	0.50	0.50	0.75
Sox2	0.87	0.00	0.00	0.89	0.86	0.86	0.19	1.00	0.00	0.00	1.00	0.50	0.50	0.25
Cdx2	0.00	0.00	0.87	0.00	0.00	0.00	0.88	0.00	0.00	1.00	0.00	0.00	0.00	0.50
Shh	0.08	0.00	0.00	0.08	0.86	0.86	0.01	0.00	0.00	0.00	0.00	0.50	0.50	0.00

Supplementary Table S19: Comparing model predictions and relative expression change of downstream nodes of signaling pathways. The gene expression of downstream nodes of some signaling pathways, including Ras/MAPK, VEGF, NF-κB and Wnt/β-catenin signaling were checked in high-throughput data. The comparison indicated that 67% (GSE 19826) and 66.7% (GSE 22183) of the relative gene expression changes of downstream nodes predicted by the modeling results were consistent with high-throughput data.

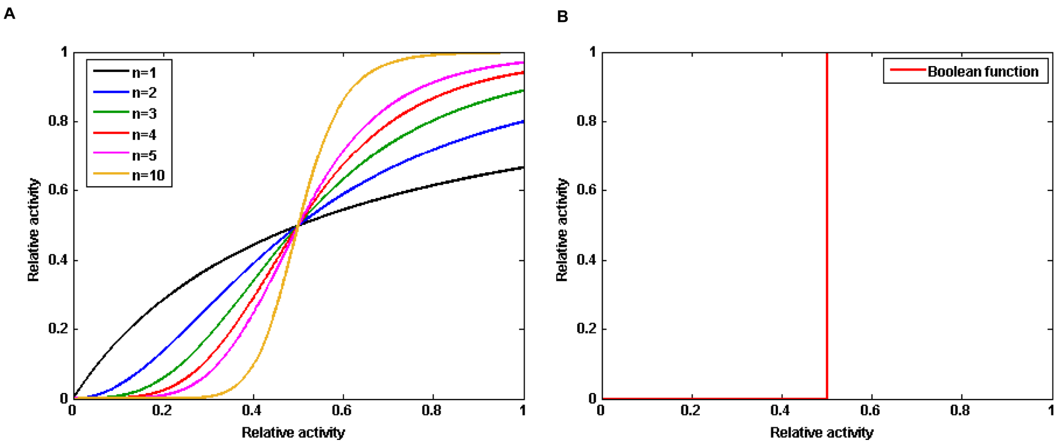

Supplementary Figure S1: Hill functions and Boolean functions. Hill functions with Hill coefficients n = 1, 2, 3, 4, 5, 10 and a = 2^n as continuous relaxation of a Boolean step function. **A.** Hill functions; **B.** Boolean functions.

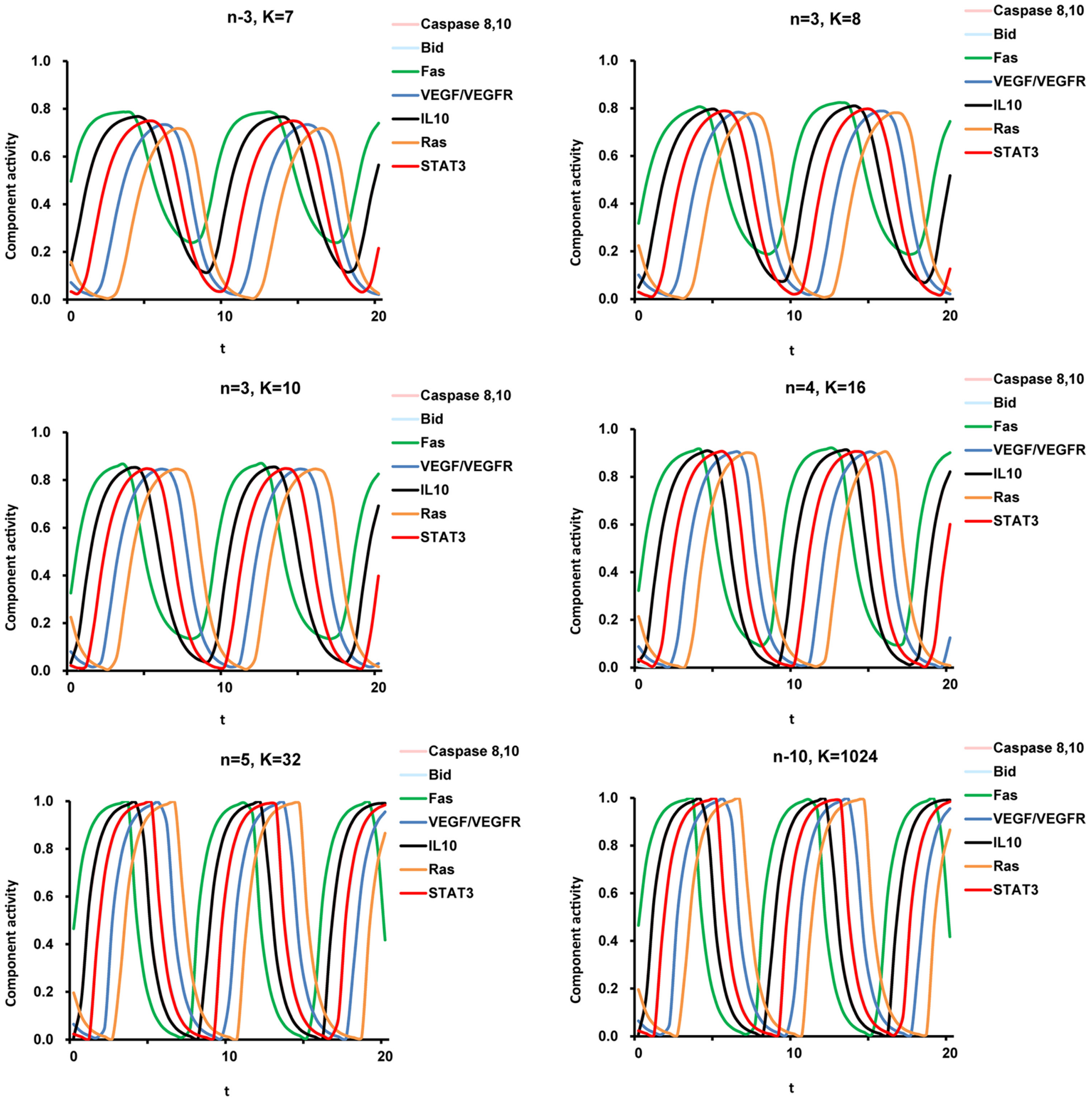

Supplementary Figure S2: Calculated dynamical trajectories of cell death attractor under scanning parameters. Dynamical trajectories of Caspase 8/10, Bid, Fas, VEGF/VEGFR, IL10, Ras and STAT3.

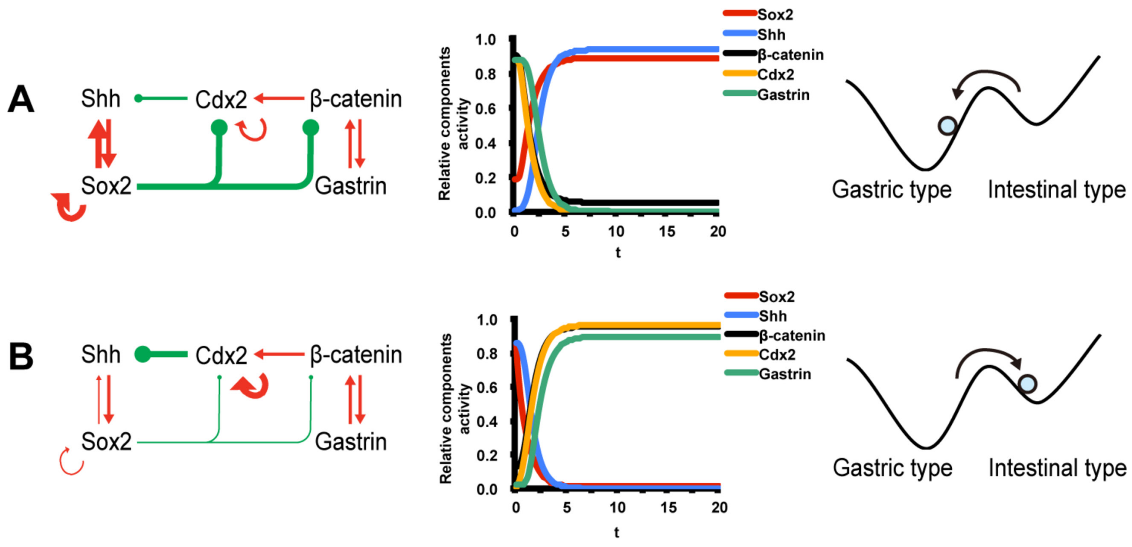

Supplementary Figure S3: Transition between gastric- and intestinal-type attractors. The working model indicated that intestinal-type attractor can be induced to gastric-type attractor by consistently activating Sox2 (Supplementary Figure S2A), whereas gastric-type attractor can be induced to intestinal-type attractor by activating Cdx2 and inhibiting Sox2 simultaneously (Supplementary Figure S2B).

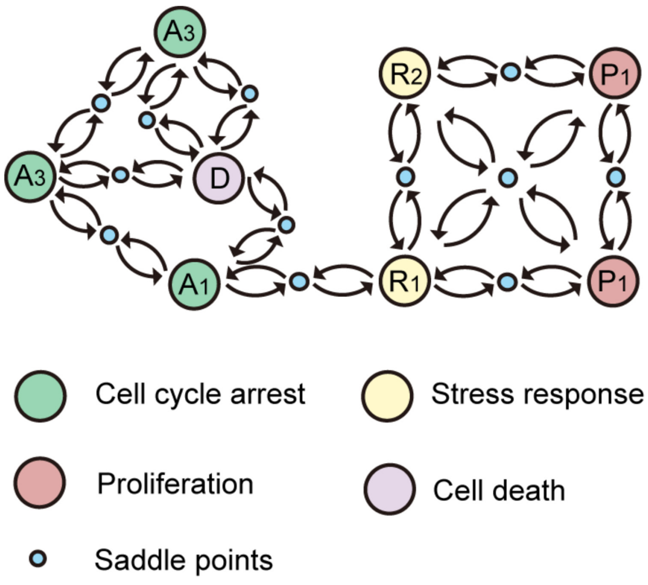

Supplementary Figure S4: Schematic illustration of the functional landscape for the core endogenous molecular network of gastric epithelium. Large circles represent attractors indicating 8 cellular phenotypes. Blue circles represent saddle points, which are unstable equilibrium states. Arrows denote that a transition can occur between two attractors. A_1, A_2 and A_3, cell cycle arrest; D, cell death; R_1 and R_2, stress response; P_1 and P_2, proliferation.

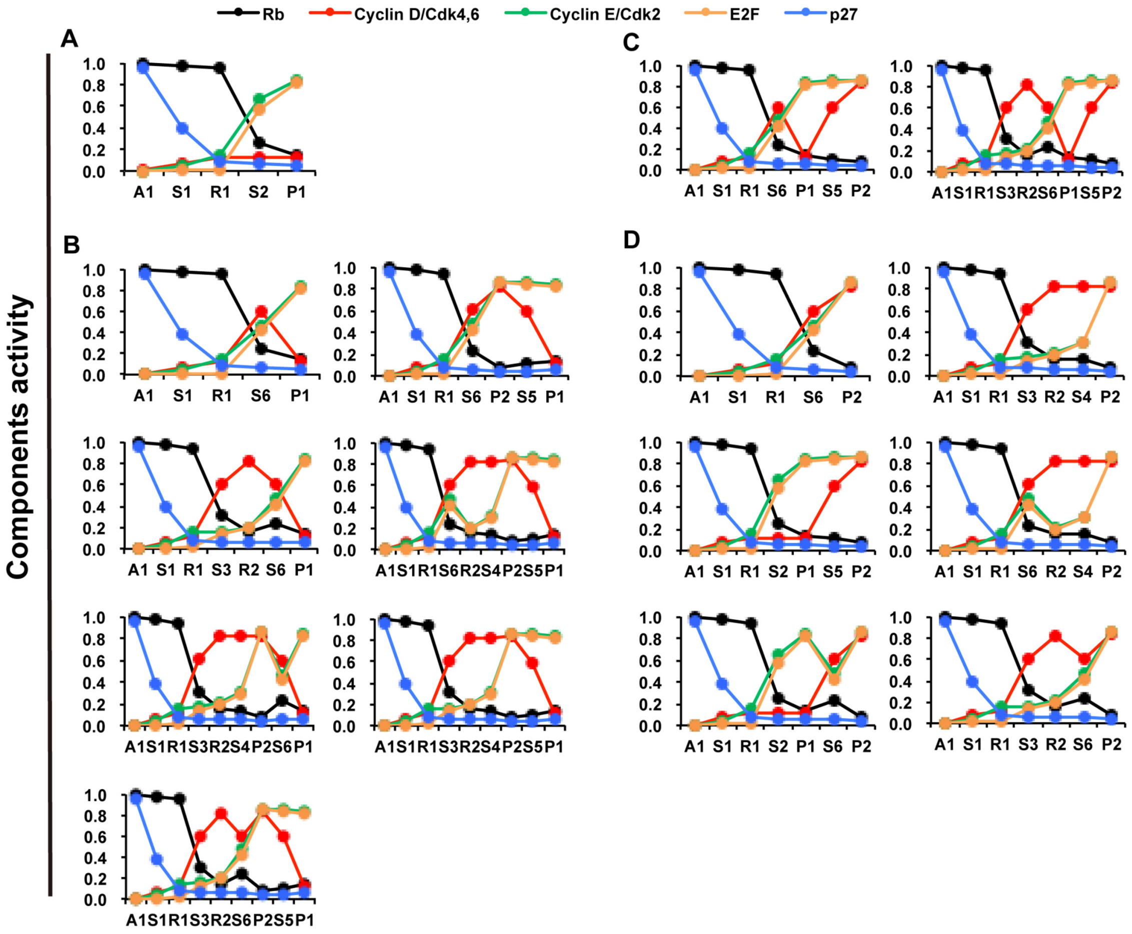

Supplementary Figure S5: Dynamics of cell cycle components in the routes of GC progression. A–D. shows activity dynamics of cell cycle components in 16 possible inherent evolutional routes from normal gastric epithelial attractor A_1 to GC attractors P_1 and P_2. The dynamic process of certain critical regulators exhibit 4 major patterns of GC progression: **A.**-(.A_1, A_2 and A_3, cell cycle arrest; D, cell death; R_1 and R_2, stress response; P_1 and P_2, proliferation; S1–S14, saddle points.

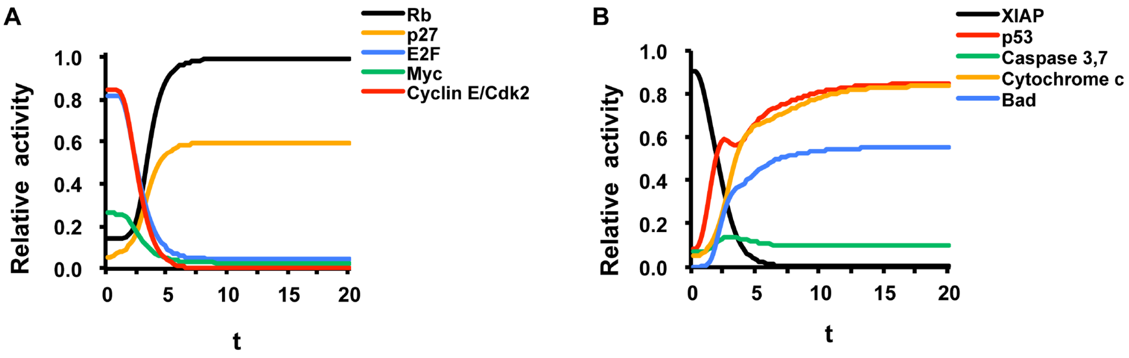

Supplementary Figure S6: Dynamics of cell cycle and apoptosis components activities under consistently inhibiting PI3K/Akt activity. A. When PI3K/Akt activity was manually inhibited in GC attractor, the activities of cell cycle components E2F, Myc and CyclinE/Cdk2 were inhibited, while the activities of cell cycle arrest related components Rb and p27 were activated. GC attractor transformed to cell cycle arrest like attractor by consistently inhibiting PI3K/Akt activity. **B.** When PI3K/Akt activity was manually inhibited in GC attractor, the activities of anti-apoptotic components XIAP was inhibited, while the activities of pro-apoptotic related components p53, Cytochrome *c* and Bad were activated. GC attractor transformed to apoptosis like attractor by consistently Inhibiting PI3K/Akt activity.
